# P–Ru-Complexes with a Chelate-Bridge-Switch: A Comparison of 2-Picolyl and 2-Pyridyloxy Moieties as Bridging Ligands

**DOI:** 10.3390/molecules27092778

**Published:** 2022-04-27

**Authors:** Lisa Ehrlich, Robert Gericke, Erica Brendler, Jörg Wagler

**Affiliations:** 1 Institut für Anorganische Chemie, TU Bergakademie Freiberg, D-09596 Freiberg, Germany; ehrlich@ipfdd.de (L.E.); gericker.chemie@gmail.com (R.G.); 2 Institute of Resource Ecology, Helmholtz-Zentrum Dresden-Rossendorf eV, D-01328 Dresden, Germany; 3 Institut für Analytische Chemie, TU Bergakademie Freiberg, D-09596 Freiberg, Germany; erica.brendler@chemie.tu-freiberg.de

**Keywords:** atoms-in-molecules, hemilabile, phosphane, ruthenium, X-ray diffraction

## Abstract

Starting from [Ru(pyO)_2_(nbd)] **1** and a N,P,N-tridentate ligand (**2a**: PhP(pic)_2_, **2b**: PhP(pyO)_2_) (nbd = 2,5-norbornadiene, pic = 2-picolyl = 2-pyridylmethyl, pyO = 2-pyridyloxy = pyridine-2-olate), the compounds [PhP(μ-pic)_2_(μ-pyO)Ru(κ^2^-pyO)] (**3a**) and [PhP(μ-pyO)_3_Ru(κ^2^-pyO)] (**3b**), respectively, were prepared. Reaction of compounds **3** with CO and CN*t*Bu afforded the opening of the Ru(κ^2^-pyO) chelate motif with the formation of compounds [PhP(μ-pic)_2_(μ-pyO)Ru(κ-*O*-pyO)(CO)] (**4a**), [PhP(μ-pic)_2_(μ-pyO)_2_Ru(CN*t*Bu)] (**5a**), [PhP(μ-pyO)_4_Ru(CO)] (**4b**) and [PhP(μ-pyO)_4_Ru(CN*t*Bu)] (**5b**). In dichloromethane solution, **4a** underwent a reaction with the solvent, i.e., substitution of the dangling pyO ligand by chloride with the formation of [PhP(μ-pic)_2_(μ-pyO)Ru(Cl)(CO)] (**6a**). The new complexes **3a**, **4a**, **5a**, **5b** and **6a** were characterized by single-crystal X-ray diffraction analyses and multi-nuclear (^1^H, ^13^C, ^31^P) NMR spectroscopy. The different coordination behaviors of related pairs of molecules (i.e., pairs of **3**, **4** and **5**), which depend on the nature of the P–Ru-bridging ligand moieties (μ-pic vs. μ-pyO), were also studied via computational analyses using QTAIM (quantum theory of atoms in molecules) and NBO (natural bond orbital) approaches, as well as the NCI (non-covalent interactions descriptor) for weak intramolecular interactions.

## 1. Introduction

**Scheme 1:**
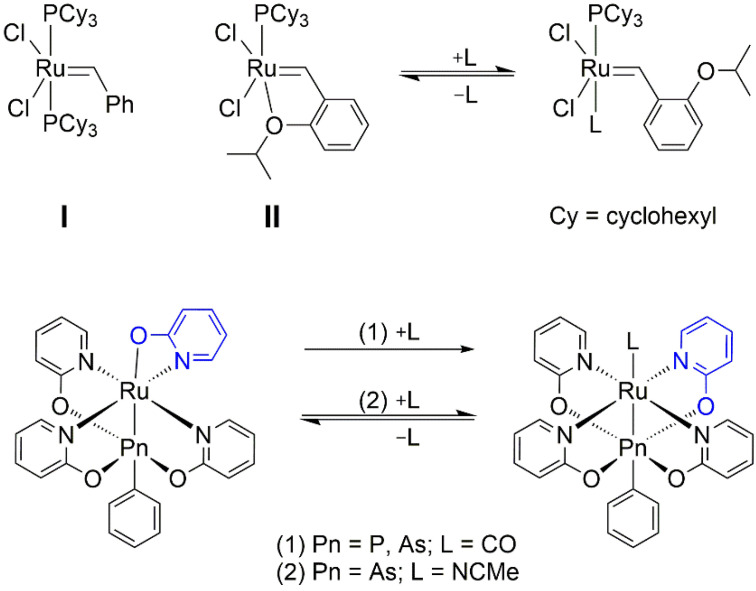
Top: First-generation Grubbs catalyst (**I**) and an analogous first-generation Grubbs–Hoveyda catalyst (**II**) with a hemilabile ligand. L may represent a substrate (olefin) undergoing transformation in the Ru coordination sphere. Bottom: Hemilabile features of a Ru-bound κ^2^-pyO ligand, with switching of the Ru-bound O atom toward the adjacent pnictogen (Pn) ligand atom.

Hemilabile ligands are crucial in various kinds of homogeneous catalysis [1,2]. As an example of a relevant Ruthenium-based system, the introduction of a hemilabile site allowed for the development of the well-known Grubbs catalysts (**I**, Scheme 1), leading to the Grubbs–Hoveyda catalysts (**II**) [3]. The labile site of the hemilabile ligand stabilizes the metal complex in the absence of alternative electron pair donors, but it may give rise to a vacant coordination site for substrate binding “on demand”. The labile ligator function remains a dangling group in close proximity. Recently, we reported on Ru complexes with 2-pyridyloxy (pyO) ligands, in which a chelating pyO group may exert hemilability by means of a coordinative switch between two centers. Rather than simply opening the chelate (with the formation of a monodentate pyO ligand with a dangling second donor site), the κ^2^-pyO motif is converted into a bridging μ-pyO motif within a paddle-wheel-like complex, then buttressing the connection to another ligand site in the Ru coordination sphere (Scheme 1) [4,5]. Starting from the initial [PhP(μ-pyO)_3_Ru(κ^2^-pyO)] system, we have shown that this switch also works for the related As–Ru system, and CO (for P–Ru and As–Ru) and NCMe (for As–Ru) were able to trigger this switch. In the related complex [PhSb(μ-pyO)_4_Ru(NCMe)], the rather strong binding of pyO to the Sb atom did not allow for the release of NCMe with the formation of a corresponding [PhSb(μ-pyO)_3_Ru(κ^2^-pyO)] chelate complex.

As only one pyO ligand is involved in switching, the aim of the current study was the exploration of the role of the remaining buttresses across the P–Ru core. In previous studies, we had explored systems with other (O,N)-bidentate ligands as well (anions of *N*-methylbenzamide and phthalimidine), which preferred the bridging position in Pn-Ru-complexes (Pn = pnictogen) and showed no tendency to form chelates at Ru [6,7,8]. However, for the current study with systems of well-defined combinations of bridging ligands, we wanted to retain the pyridine motif as the Ru-binding site, and we also wanted to avoid ligand scrambling, thereby using a bridging moiety tightly bound to phosphorus. Therefore, 2-picolyl (pic) bridges were introduced in this molecular system, and reactions and molecular structures were compared for pairs of corresponding pic-vs.-pyO-bridged compounds. In a similar manner as pyO had been used as a bridging ligand in dinuclear complex systems of transition metals and heavier main group elements such as (RuAs) [5,9], (CuSi), (PdSi) [10], (IrSi) [11], (CoSi) [12] and (RhBi) [13], picolyl bridges have also been successfully used as bridging moieties, e.g., for (CoSi) [14], (FeP) [15], (PdSe) [16] and (PdTe) [17] systems.

## 2. Results and Discussion

### 2.1. Syntheses

**Scheme 2:**
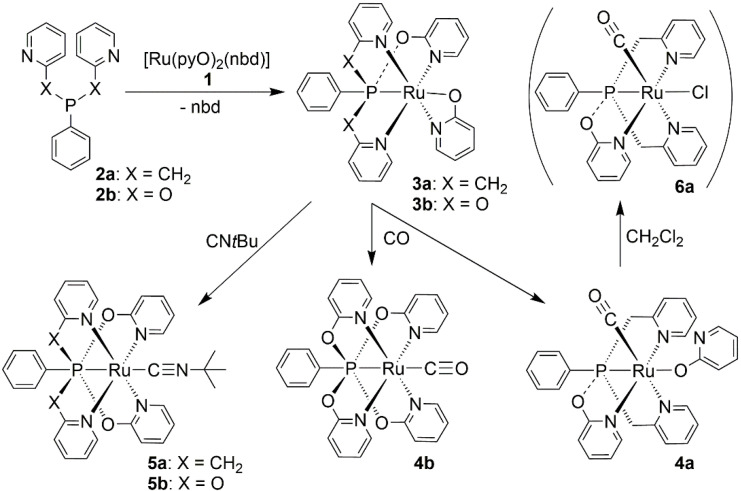
Syntheses of the compounds under investigation.

The starting materials, i.e., [Ru(pyO)_2_(nbd)] **1** [4], phosphanes **2a** (i.e., phenylbis(2-pyridylmethyl)phosphane) [15] and **2b** (i.e., phenylbis(2-pyridyloxy)phosphane) [8], and complex **3b** ([PhP(μ-pyO)_3_Ru(κ^2^-pyO)]) [4], have been reported in the literature and were prepared following the protocols reported previously. As **3b** was accessible in a straightforward manner from **1** and **2b**, our synthesis of **3a** ([PhP(μ-pic)_2_(μ-pyO)Ru(κ^2^-pyO)]) followed the same route (Scheme 2). Whereas **3b** was crystallized by vapor diffusion of diethyl ether into a dichloromethane (DCM) solution of **3b**, crystallization of compound **3a** was successful upon vapor diffusion of *n*-pentane into a DCM solution of **3a** (isolated yield 68%). Additionally, synthesis of compound **4a** ([PhP(μ-pic)_2_(μ-pyO)Ru(κ-*O*-pyO)(CO)]) was carried out following the protocol reported for the synthesis of compound **4b** ([PhP(μ-pyO)_4_Ru(CO)]) [4], i.e., a dispersion of **3a** in toluene was exposed to CO atmosphere at 60 °C for 3 days (isolated yield < 35%, product still contained starting material **3a**). The reaction of **3a** (in DCM) or **3b** (in toluene) with CN*t*Bu afforded crystals of isonitrile complexes **5a** ([PhP(μ-pic)_2_(μ-pyO)_2_Ru(CN*t*Bu)], upon vapor diffusion of *n*-pentane, a few crystals only) or **5b** ([PhP(μ-pyO)_4_Ru(CN*t*Bu)], upon cooling of the toluene solution, isolated yield 53%), respectively. In an attempt to recrystallize a small amount of **4a** from DCM (because **4a** was contaminated by **3a**, see NMR Section 2.3, and because the crystal structure of the toluene solvate of **4a** suffered from heavy disorders, see Section 2.2), a few crystals of **6a** ([PhP(μ-pic)_2_(μ-pyO)Ru(Cl)(CO)]) were obtained.

### 2.2. Single-Crystal X-ray Diffraction

**Figure 1:**
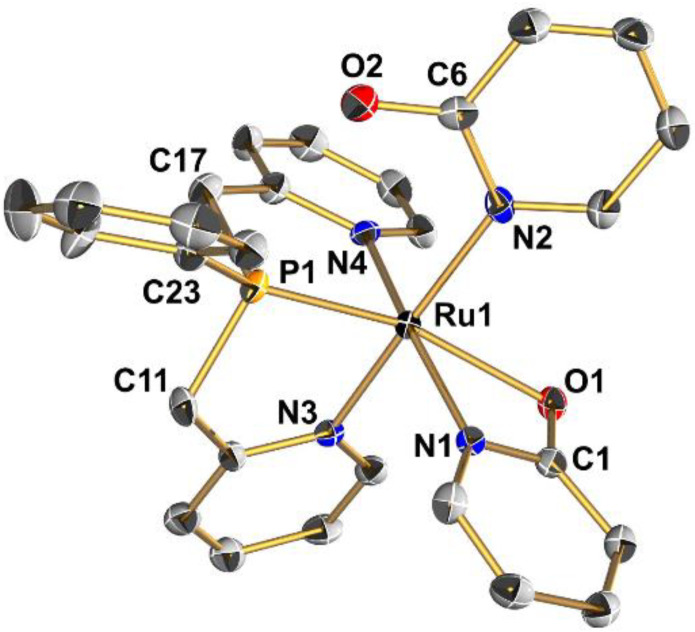
Molecular structure of **3a** in the crystal (thermal displacement ellipsoids drawn at the 50% probability level, selected atoms labeled, H-atoms are omitted for clarity). The asymmetric unit consists of two molecules of **3a** in very similar conformation; thus, only one of them is shown as a representative example. Selected interatomic distances (Å) and angles (deg.) of **3a**: Ru1–P1 2.1887(11), Ru1–O1 2.283(4), Ru1–N1 2.076(6), Ru1–N2 2.124(7), Ru1–N3 2.082(6), Ru1–N4 2.070(6), P1–C23 1.822(4), P1–C11 1.857(7), P1–C17 1.839(7), P1···O2 2.792(5), P1-Ru1-O1 165.90(11), Ru1-P1-C23 136.49(14), C11-P1-C17 105.7(3), O2-P1-C11 175.9(2).

Compound **3a** crystallized in the orthorhombic space group *Pna*2_1_ with two independent (but conformationally very similar) molecules in the asymmetric unit (Figure 1). The molecular configuration of **3a** is the same as in the known compound **3b** [4], i.e., the Ru atom is located in a distorted octahedral coordination sphere with a RuN_4_ equatorial plane and *trans*-situation of P- and O-donor sites. In both compounds, one pyO ligand chelates at Ru, and the Ru–P bond is bridged by three buttresses ((pic)_2_(pyO) in **3a**, (pyO)_3_ in **3b**).

**Figure 2:**
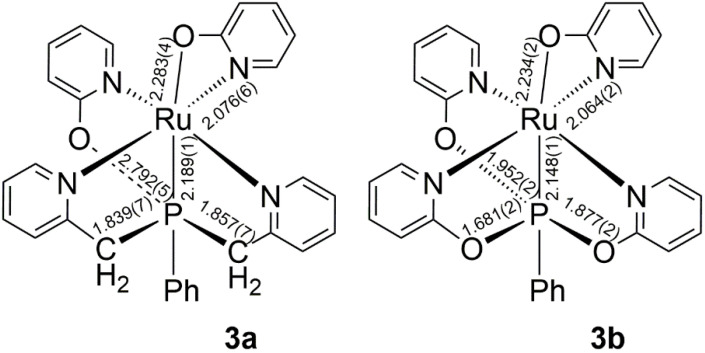
Comparison of corresponding interatomic separations (Å) in the molecular structures of **3a** (the molecule shown in Figure 1) and **3b** [4].

Two noteworthy differences between **3a** and **3b** are found in the Ru-P-C(phenyl) angles (136.5(2)° in **3a**, 147.3(1)° in **3b**) and in the P–O separations (Figure 2). The pic substituted P atom of **3a** is less Lewis acidic than the pyO-substituted counterpart in **3b**. The bridging pyO moiety in **3a** establishes a rather weak P···O contact (2.792(5) Å), whereas in **3b** the longest P–O separation (1.952(2) Å) is much shorter. Thus, the less pronounced widening of the Ru-P-C(phenyl) angle in **3a** can be attributed to this limited P···O interaction. Nonetheless, in **3a** the P–CH_2_ *trans*-bond to this weak P···O coordination is slightly elongated. Interestingly, both the Ru–P and Ru–O bonds of **3a** are longer than their corresponding bonds in **3b**. We attribute this to the weaker π-acceptor phosphane in **3a**, which may weaken both bonds at the same time by causing lower Ru→P π-back-bonding contributions and lower resulting O→Ru π-donation.

**Figure 3:**
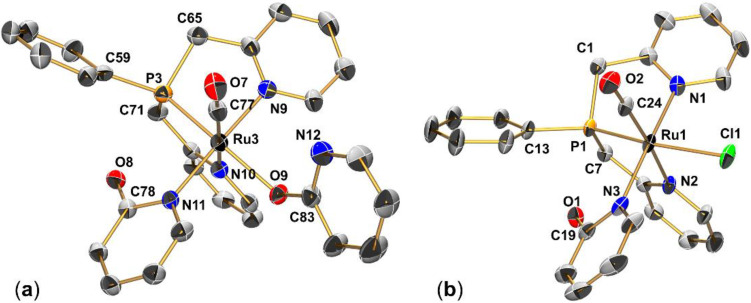
(**a**) Molecular structure of **4a** in the crystal structure of **4a**·(toluene) (thermal displacement ellipsoids drawn at the 30% probability level, selected atoms labeled, H-atoms and solvent molecules are omitted for clarity). The asymmetric unit contains four molecules of **4a** in very similar conformation; thus, only one of them is shown as a representative example. Selected interatomic distances (Å) and angles (deg.) of **4a**: Ru3–P3 2.2237(14), Ru3–O9 2.143(3), Ru3–N9 2.119(5), Ru3–N10 2.190(4), Ru3–N11 2.110(4), Ru3–C77 1.842(6), P3–C59 1.802(6), P3–C65 1.859(6), P3–C71 1.825(5), P3···O8 2.701(4), O7–C77 1.144(6), P3-Ru3-O9 167.55(10), Ru3-P3-C59 131.97(18), C65-P3-C71 105.4(3), O8-P3-C65 176.9(2). (**b**) Molecular structure of **6a** in the crystal structure of **6a**·1.5(CH_2_Cl_2_) (thermal displacement ellipsoids drawn at the 30% probability level, selected atoms labeled, H-atoms and solvent molecules are omitted for clarity), selected interatomic distances (Å) and angles (deg.) of **6a**: Ru1–Cl1 2.4868(7), Ru1–P1 2.2146(7), Ru1–N1 2.134(3), Ru1–N2 2.175(2), Ru1–N3 2.095(3), Ru1–C24 1.837(3), P1–C1 1.836(3), P1–C7 1.825(3), P1–C13 1.804(3), P1···O1 2.637(2), O2–C24 1.147(3), P1-Ru1-Cl1 170.70(3), Ru1-P1-C13 132.34(11), C1-P1-C7 104.23(14), O1-P1-C1 178.29(11).

**Figure 4:**
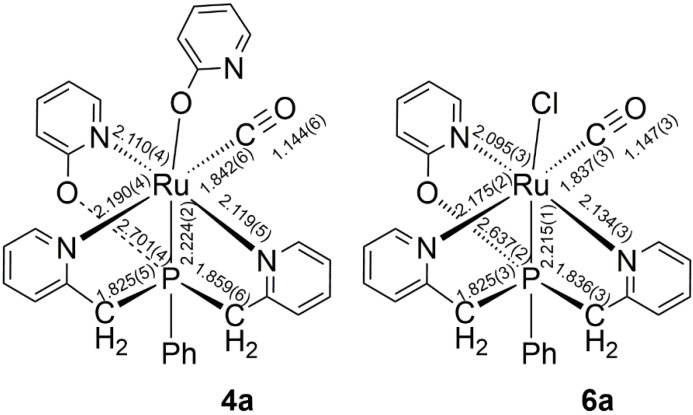
Comparison of corresponding interatomic separations (Å) in the molecular structures of **4a** and **6a** (data for the molecules shown in Figure 3).

Compound **4a** crystallized from the mother solution as a toluene solvate in the monoclinic space group *P*2_1_/*c* (Figure 3a). The molecular configuration of **4a** exhibits two striking differences to the presumed analog **4b** [4]. Whereas the latter resembles a paddle-wheel-shaped molecule with *trans* situation of P- and CO-ligands at Ru, with all pyO moieties bound to Ru through Ru–N bonds, compound **4a** exhibits *cis* arrangement of P- and CO-ligands at Ru with an only three-fold buttressed Ru–P bond. Furthermore, the monodentate pyO in **4a** is Ru–O-bound with a dangling N donor site. This configuration about the Ru–P core is essentially retained in compound **6a**, which is crystallized from dichloromethane solution as a DCM solvate in the monoclinic space group *P*2_1_/*n* (Figure 3b). Even though the molecular configuration of **4a** was unexpected at first sight (with respect to the known paddle–wheel shape of **4b**), [Ru(dppe)(CO)(NCMe)_3_][OTf]_2_ [18] is an example of another octahedral Ru(II) complex in which the CO ligand occupies a *cis*-P,*trans*-N position, even though sterics would allow for the alternative *cis*-N,*trans*-P arrangement. As for the Ru-O-bound monodentate pyO moiety, a paddle-wheel complex with a Ru≡Ru axis, which has been reported by Powers et al., also bears a Ru-O-bound pyO derivative [19]. Because of the configurational analogies of **4a** and **6a**, Figure 4 provides a direct comparison of these two molecular structures. Their Ru-P-C(phenyl) angles (132.0(2)° in **4a** and 132.3(1)° in **6a**) are essentially identical, and replacement of pyO by Cl (i.e., replacing of one π-donor ligand by another π-donor) did not alter the Ru–P bond length in a noteworthy manner. Additionally, in both compounds the dangling pyO oxygen atom coordinates the P atom from a remote position with slightly different separations of the P···O contacts (2.701(4) in **4a** vs. 2.637(2) Å in **6a**), with also only a small difference between the molecular shapes of **4a** and **6a** and similar to the corresponding P···O interatomic distance in **3a**. Also related to **3a** is the slight P–CH_2_ bond elongation *trans* to this P···O contact. In the Ru coordination sphere, the corresponding Ru–N and Ru–C bonds also exhibit similar lengths. In this regard, the Ru–N *trans-*bond to the CO ligand is significantly longer than the other two Ru–N bonds of the mutually *trans*-situated pyridine moieties. We attribute this structural feature to weakened Ru→N π-back-bonding *trans* to the CO ligand, which itself causes strong Ru→C π-back-bonding. The latter is indicated by the C≡O stretching vibration at 1931 cm^−1^, which gives rises to a strong band in the IR spectrum of compound **4a**. This is just at slightly higher wave numbers than the C≡O stretch found for compound **4b** (which is at 1921 cm^−1^) and indicative of strong Ru→C π-back-bonding. Additionally, this structural feature of a long Ru–N(pyridine) bond *trans* to a CO ligand can be found in other octahedral Ru(II) complexes, e.g., in the cationic complex [Ru(phen)(bpy)(CO)Cl]^+^ with *cis*-arranged monodentate ligands [20].

**Figure 5:**
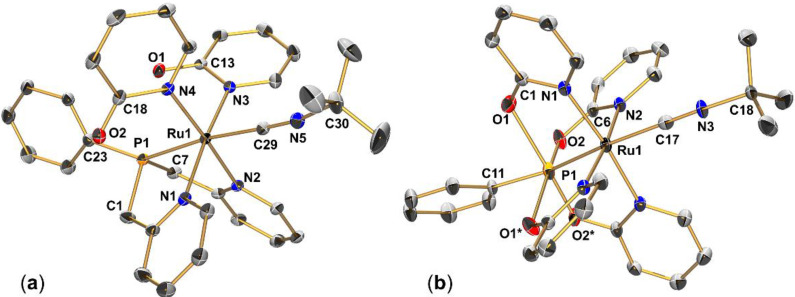
(**a**) Molecular structure of **5a** in the crystal structure of **5a**·1.5(CH_2_Cl_2_) (thermal displacement ellipsoids drawn at the 30% probability level, selected atoms labeled, H-atoms and solvent molecules are omitted for clarity), selected interatomic distances (Å) and angles (deg.) of **5a**: Ru1–P1 2.3080(4), Ru1–N1 2.1087(14), Ru1–N2 2.1074(14), Ru1–N3 2.1359(14), Ru1–N4 2.1006(14), Ru1–C29 1.9569(17), P1–C23 1.8243(18), P1–C1 1.8440(18), P1–C7 1.8342(18), P1···O1 2.8135(14), P1···O2 2.7699(14), N5–C29 1.162(2), P1-Ru1-C29 173.01(5), Ru1-P1-C23 143.85(6), C1-P1-C7 102.96(7), O1-P1-C1 174.21(8), O2-P1-C7 174.72(7). (**b**) Molecular structure of **5b** in the crystal (thermal displacement ellipsoids drawn at the 30% probability level, selected atoms labeled, H-atoms are omitted for clarity). Some atoms of the molecule (e.g., C11, P1, Ru1, C17, N3, C18) are located on a crystallographically imposed bisecting plane. Therefore, the asymmetric unit consists of one half of the molecule. Symmetry-equivalent atomic labels are asterisked. Selected interatomic distances (Å) and angles (deg.) of **5b**: Ru1–P1 2.2700(14), Ru1–N1 2.094(3), Ru1–N2 2.119(3), Ru1–C17 1.995(6), P1–C11 1.821(5), P1–O1 2.309(3), P1–O2 1.713(3), N3–C17 1.156(7), P1-Ru1-C17 170.65(15), Ru1-P1-C11 158.11(17), O2-P1-O2* 91.4(2), O1-P1-O2* 170.48(14).

Compound **5a** crystallized as a DCM solvate in the triclinic space group *P*$\bar{1}$ (Figure 5a) and the analogous isonitrile complex **5b** crystallized in the orthorhombic space group *Pnma* (Figure 5b). In this set, the molecules adopt related configurations with *trans*-situated P- and isonitrile-ligands at the RuN_4_ core. The most striking difference is associated with the different P-O-binding of the pyO bridging ligands. Even though the pyO oxygen atoms approach the P atom in a very direct manner (resulting in O-P-C angles in **5a** and O-P-O angles in **5b** wider than 170°), in **5a** two rather long P···O contacts (2.814(2) and 2.770(2) Å) leave room for a smaller Ru-P-C(phenyl) angle (143.9(1)°), whereas in **5b** the stronger binding of the pyO oxygen atoms (P–O separations of 1.713(3) and 2.309(3) Å) causes noticeable widening of the Ru-P-C(phenyl) angle (158.1(2)°), thereby approaching a distorted octahedral coordination sphere of the P atom in **5b**. In accordance with the pair **3a**/**3b**, compound **5a** exhibits a longer Ru–P bond than compound **5b** (2.308(1) vs. 2.270(2) Å), which again can be attributed to the weaker π-acceptor phosphane in **5a**. The *trans*-disposed Ru–C bond, however, is slightly shorter in **5a** (1.957(2) vs. 1.995(6) Å). As isonitriles are ligands with significant π-acceptor features, the Ru1–C29 bond in **5a** is likely to respond to the weaker competing π-acceptor phosphane with enhanced Ru→C π-back-bonding. This explanation is supported by the slightly longer Ru–C bond (2.009(6) Å) in complex [Ru_2_(CO)_5_(*t*BuNC)(bpcd)] (bpcd = 4,5-bis(diphenylphosphino)-4-cyclopenten-1,3-dione) [21], which also features a *t*BuNC-Ru-P *trans*-arrangement and additional strong π-acceptor ligands. Furthermore, it is backed by IR spectroscopic data (bands of the CN stretch) for complexes **5a** and **5b** (see Appendix B).

### 2.3. Solution and Solid State NMR Characterization

**Figure 6:**
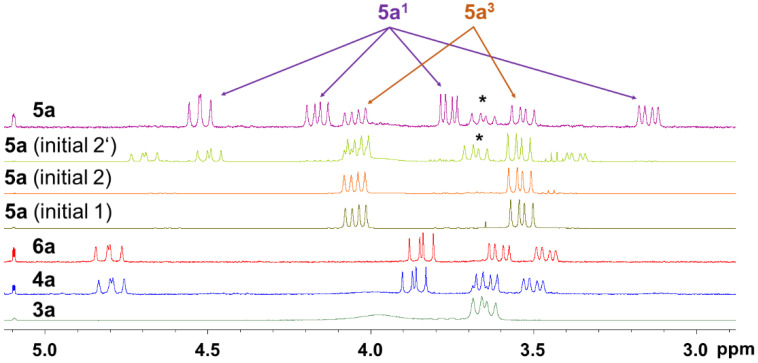
Section of the CH_2_ signals of the ^1^H NMR spectra of (from bottom to top) **3a**, **4a**, **6a** and **5a** in CD_2_Cl_2_ (the signal at 5.1 ppm is a ^1^*J*(^13^C^1^H) satellite of CDHCl_2_ solvent signal, the asterisks indicate signals of **3a**).

In the solid state, compound **3a** bears a set of two chemically non-equivalent pyO-ligands (a chelating and a bridging group), and resulting therefrom two non-equivalent pic-moieties. In dichloromethane solution, the two types of pyO ligands undergo rather rapid exchange (with respect to the NMR time scale), as both the ^1^H and ^13^C NMR spectra of **3b** exhibit one set of signals for pyO- and one for pic-ligands (for ^1^H, ^13^C and ^31^P NMR spectra of **3a** and of compounds **4a**, **5a**, **5b** and **6a**, see Figures S1–S19 in the Supplementary Materials). Especially in the ^1^H spectrum, the former signals are noticeably broadened and do not exhibit coupling patterns (related exchange of pyO-ligands was found in **3b** [4]). As to the pic-ligands, only their CH_2_ groups suffer severe signal broadening both in ^1^H and ^13^C spectra. With respect to the phosphorus coordination sphere, the average coordination number in solution is close to the situation in the solid state, the ^31^P NMR shift in CD_2_Cl_2_ solution (80.8 ppm) is just slightly shifted downfield relative to the values found for the two crystallographically independent P sites in solid **3a** (76.3 and 73.8 ppm). With respect to the ^31^P NMR shift of the free phosphane PhP(pic)_2_ (δ^31^P = −13.7 ppm in CDCl_3_), the corresponding NMR signal of **3a** is shifted downfield by about 90 ppm. The products arising from **3a** (i.e., **4a**, **5a** and **6a**) may exhibit different configurations, which will be indexed with superscript-1 and superscript-3 in accord with the isomers under investigation by computational analyses (vide infra). In the crystal structures (Figure 3 and Figure 5), we encountered **4a^1^**, **5a^3^** and **6a^1^**. In solution ^1^H NMR spectra, the pic-CH_2_ groups are a convenient probe for assigning configurations 1 and 3, as in the former the molecules bear four chemically non-equivalent CH_2_ protons, while in the latter the molecules bear two symmetry-related CH_2_ groups with diastereotopic protons. Figure 6 shows the section of CH_2_ signals of the ^1^H NMR spectra of compounds **3a**, **4a**, **5a** and **6a**.

**Figure 7:**
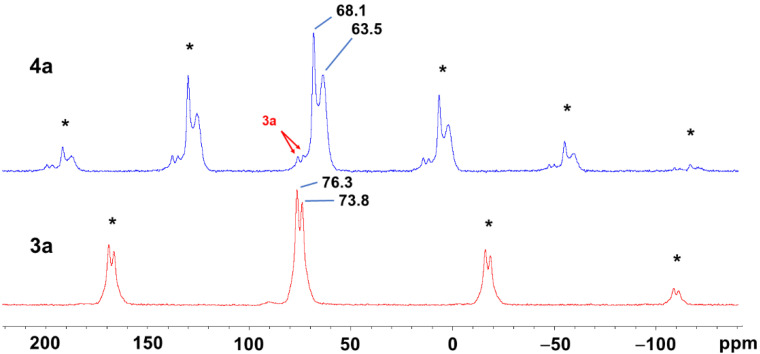
^31^P CP/MAS NMR spectra of **3a** (bottom) and **4a** (top) recorded at spinning frequencies of 15 kHz and 10 kHz, respectively. (The chemical shifts are given for the isotropic signals. The asterisked signals are spinning sidebands).

Compound **6a** (devoid of a second pyO group) must exhibit a configuration with four chemically independent CH_2_ protons. Its ^1^H spectrum clearly shows the four signals, which are “dd” patterned by ^2^*J*(^31^P^1^H) and ^2^*J*(^1^H^1^H) coupling for each proton (Figure 6, red). Apart from the signals of small amounts of **3a**, the spectrum of **4a** exhibits essentially the same signal pattern as **6a**, showing retention of configuration **4a^1^** in the solution (Figure 6, blue). The ^31^P NMR signal of **4a** in the CD_2_Cl_2_ solution (δ^31^P = 68.6 ppm) is slightly shifted upfield with respect to the signal of **3a**. In the solid state, ^31^P NMR signals of **4a** were found at 63.5 and 68.1 ppm (Figure 7). Again, the set of crystallographically independent molecules gives rise to more than just one signal. Furthermore, the ^31^P CP/MAS NMR spectrum of **4a** indicates the presence of **3a** in the solid product. Hence, the signals of **3a** encountered in solution spectra of **4a** arose from impurities in the solid product rather than from decomposition of **4a** with release of CO.

Upon addition of some drops of CN*t*Bu to a CD_2_Cl_2_ solution of **3a**, which corresponds to large excess of the isonitrile, spectra **5a** (initial 1) and **5a** (initial 2) were obtained (sample **5a** (initial 1) contained somewhat larger excess of CN*t*Bu, while in sample **5a** (initial 2) there was only a 15% excess according to integral traces of Ru-bound and free CN*t*Bu), cf. Figure 6 (olive, orange). The appearance of only two “dd”-patterned CH_2_ proton signals indicates the formation of configurational isomer **5a^3^** under these conditions, which is in accordance with the structure of **5a** found in the solid state. Within 20 h, the latter sample had undergone some decomposition (pale green spectrum **5a** (initial 2′)). Large amounts of **3a** formed (thus, **5a^3^** must have released CN*t*Bu), and CH_2_ signals of a new complex appeared. (Unfortunately, we were not able to isolate this new compound by layering the NMR sample solution with *n*-pentane at this stage.) Upon dissolution of some crystals of the isolated **5a** in CD_2_Cl_2_, however, a ^1^H NMR spectrum was obtained, which indicated the presence of both isomers **5a^3^** and presumably **5a^1^** (the coupling patterns of the four CH_2_ signals are very well in accordance with those of **4a** and **6a**), as well as significant amounts of **3a** (Figure 6, purple). Again, the latter can be explained by dissociation of **5a** in solution with formation of **3a** and CN*t*Bu in a dynamic equilibrium as the (corresponding to the proton intensities of **3a**) relative intensity of 9 H atoms was observed for the signal of free CN*t*Bu in the same spectrum. As compound **5a** (isomer **5a^3^**) forms in the first instance upon adding CN*t*Bu to **3a**, but also undergoes dissociation (with formation of **3a** and CN*t*Bu) as well as decomposition in solution (most likely driven by free CN*t*Bu), the solution NMR data reported in the experimental section are based on spectra of **5a** generated in situ. The identity of the decomposition products (apart from **3a**) has not been established yet. This initial formation of **5a^3^** and rather quick decomposition observed for this compound in DCM solution (which should include formation of **5a^1^**) serves as an explanation as to why we obtained crystalline **5a** (mixture of isomers) in very poor yield, and timely workup of the synthesis mixture was required to isolate **5a** at all.

The related compound **5b**, in CD_2_Cl_2_ solution, produced one set of ^1^H and ^13^C signals of the pyO moieties, indicating both retention of the paddle-wheel isomer **5b^3^** in solution and either the transition toward a more symmetrical arrangement of ligands (with essentially equal P–O bond lengths) or rapid exchange of the pairs of short and long *trans*-situated P–O bonds. Additionally, the ^31^P NMR shift of **5b** (28.0 ppm), which is noticeably shifted upfield with respect to the starting material **3b** (135.0 ppm) [4] and much closer to the shift of **4b** (−9.9 ppm) [4], indicates retention of the higher coordination number of the P atom of **5b** in solution. Furthermore, the ^1^*J*(P-C(Phenyl)) coupling (225 Hz for **5b** in CD_2_Cl_2_ solution, 277 Hz for **4b** [4]) indicates related coordination spheres about the P atoms of these two compounds. In contrast to **5a**, the ^1^H NMR spectrum of **5b** did not hint at the liberation of CN*t*Bu in solution. (Traces of **3b**, the presence of which is apparent in the ^31^P NMR spectrum with a signal at 136.1 ppm, must have been contaminations in the solid product).

### 2.4. Computational Analyses

#### 2.4.1. Relative Stability of Configurational Isomers

**Figure 8:**
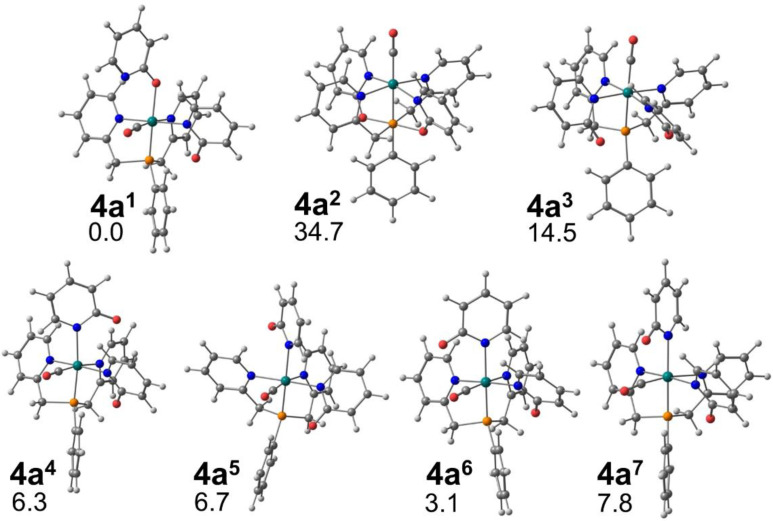
Seven different isomers of compound **4a** and their relative Gibbs free energy values in kcal mol^−1^ at 293.15 K.

The question as to why compound **4a** formed the isomer with *cis*-disposed P- and CO-ligands was addressed with the aid of computational analyses. For that purpose, the molecular structures of seven different isomers of **4a** were optimized with consideration of the effects of solvent (COSMO model for toluene), dispersion and relativistic effects. Figure 8 shows the molecular structures of the isomers. (The atomic coordinates and total energies of isomers under investigation in this chapter are listed in the Supplementary Materials, see Figures S20–S32 and Tables S1–S13. Isomer **4a^1^** corresponds to the isomer found in the crystal, cf. Figure 3a. Isomers **4a^2^** and **4a^3^** resemble paddle-wheel-shaped isomers (P-*trans*-CO arrangement) with mutually *trans*- or *cis*-disposed pic-CH_2_-groups, respectively. Another set of isomers is related to isomer **4a^1^** but with Ru-N-bound monodentate pyO ligand (*trans* to the phosphane moiety.) The hindered rotation about its Ru–N bond gave rise to four different local minima **4a^4^**–**4a^7^**. According to this analysis, the crystallographically encountered isomer **4a^1^** represents the favored isomer, whereas the paddle-wheel-shaped isomers **4a^2^** and **4a^3^** are about 35 and 15 kcal mol^−1^ less stable, respectively. The *trans*-arrangement of the CH_2_-P-CH_2_ motif (in **4a^2^**) exerts a particularly destabilizing effect. Isomers **4a^4^**–**4a^7^** (with relative energies ranging between 3 and 8 kcal mol^−1^) are noticeably more stable than the paddle-wheel forms.

As CO complex **4b** and isonitrile complexes **5a** and **5b** crystallized as isomers with configurations related to paddle-wheels (related to **4a^3^** in particular), Gibbs free energy values were analyzed for those isomers (denoted with superscript-3) in comparison with their respective isomer, which corresponds to the *cis*-P-Ru-CO arrangement and Ru-O-bound pyO in **4a^1^** (isomers denoted with superscript-1). Surprisingly, **3b^1^**, **5a^1^** and **5b^1^** were found to be the thermodynamically favored isomers. However, their paddle-wheel-shaped alternatives were only 4.3 kcal mol^−1^ (**4b^3^**) and 6.2 kcal mol^−1^ (**5b^3^**) less stable for (μ-pyO)_4_-bridged complexes. The pic-ligands, however, destabilize the paddle-wheel arrangement for isonitrile complex **5a** too (**5a^3^**: +12.9 kcal mol^−1^).

Even though solvent effects, dispersion effects and relativistic effects have been considered in the above analysis, we cannot rule out computational experimental errors which could amount to error bars of some kcal mol^−1^ for the relative energies and isomer 3 to be slightly more stable than isomer 1 in some cases. However, in the context of the observations made in solution (cf. NMR spectroscopy Section 2.3), we attribute the formation of isomers **4b^3^**, **5a^3^** and **5b^3^** to kinetic effects (preferred pyO-chelate opening with dissociation of the Ru–O bond and formation of the complex with *trans*-P-Ru-C arrangement, and if thermodynamically favorable and kinetically feasible, conversion into a different isomer). Rearrangement into the thermodynamically more stable isomer 1 would involve some steps such as changes of Ru–N- vs. Ru–O-coordination modes of a dangling pyO group and site exchange of the CO or isonitrile ligand. For complexes **4b** and **5b**, both steps are kinetically hindered to a greater extent. The O atom of the otherwise “dangling” pyO ligand is incorporated in a more or less tight bond with the P atom, and the unavailability of this O donor site appears to hinder Ru–C dissociation as well (no formation of free CN*t*Bu observed in solution of **5b**).

#### 2.4.2. P···O and C–H···(O,C) Interactions

**Figure 9:**
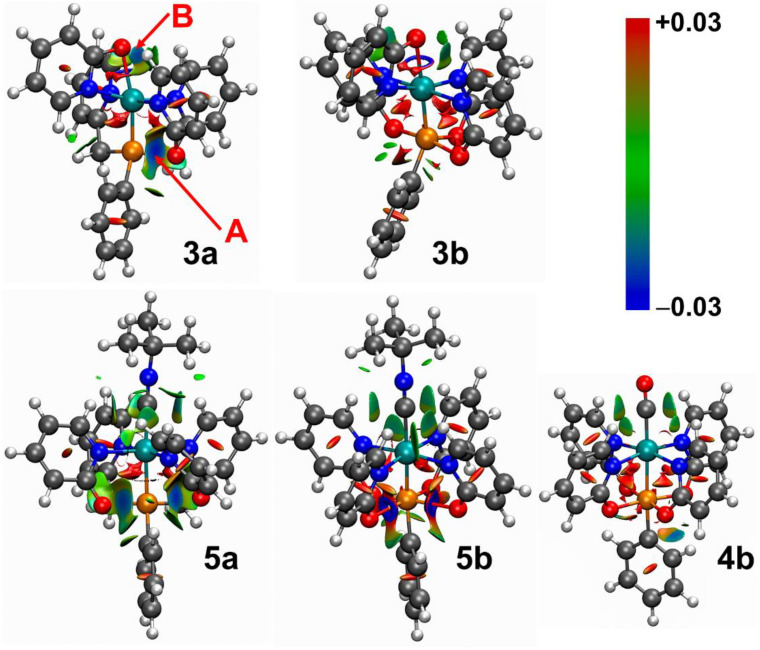
Non-covalent interactions descriptor (NCI) for compounds **3a**, **3b**, **4b**, **5a** and **5b** with color scale (iso-value 0.45; blue zones indicate attractive interaction, red zones indicate repulsive interactions).

Comparison of pairs of related molecular structures (**3a**/**3b** and **5a**/**5b**, see Section 2.2) allowed for the conclusion that pic-moieties at the phosphane ligand (in **3a** and **5a**) destabilize the bridging coordination mode of the pyO moieties in the same molecule. In order to elucidate the effect of pic- vs. pyO substitutions on different facets of bonding, computational analyses in this and the following sections were performed for structurally related molecules, which allowed for comparisons, i.e., the pair of related molecules **3a**/**3b** as well as the more or less paddle-wheel-shaped complexes **4b**, **5a** and **5b**. In the pic-functionalized compounds, bridging pyO groups establish P···O contacts with rather long interatomic separations only, which hint at weak electrostatic attraction. Therefore, we visualized the P-pic- and P-pyO-interplay with the non-covalent interactions descriptor (NCI, Figure 9). In addition to the Ru–N bond, the bridging pyO moieties establish two general types of further attractive interactions, which stabilize them in their bridging position: (A) an attractive P-O-contact and (B) an attractive hydrogen contact between H^6^ of the pyO group and the ligand atom *trans*-disposed to the phosphane. In Figure 9, these interactions are pointed out for **3a** with red arrows. Whereas the P-O-contacts in pyO-bridged complexes **3b** and **4b** are dominated by covalent interactions, the longer P-O contacts in **5b** (still exhibiting covalent contributions) are transitioning toward electrostatic interactions. In the pic-bridged complexes **3a** and **5a**, the P···O-contacts are non-covalent in nature, and according to the color scale are of similar intensity as the C–H···O contacts in **3a**. Compounds **4b**, **5a** and **5b** exhibit electrostatic attraction between the pyO-H^6^ and the C atom of the monodentate ligand *trans* to the phosphane (CO or isonitrile, respectively). This attraction, however, is less intense than the corresponding C–H···O interactions in **3a** and **3b**, which can be attributed to the lower electronegativity of C vs. O. Thus, one contribution to the driving force in the formation of isomer **4a^1^** (cf. Figure 8) can be attributed to the retention of the *trans*-P-Ru-O arrangement, which electrostatically stabilizes three bridging ligands via C–H···O interaction at the cost of an only weakly attractive potential P···O interaction (which could have been established in paddle-wheel isomer **4a^3^**).

#### 2.4.3. Topological Analysis with Quantum Theory of Atoms-In-Molecules

For the analysis of certain characteristic bond features, to supplement the insights from Section 2.4.2, a quantum theory of atoms-in-molecules (QTAIM) analysis was performed for compounds **3a**, **3b**, **4a**, **5a** and **5b**. Table 1 lists selected characteristic features for selected bonds at their (3,−1) critical points (i.e., bond critical points, BCPs). In all cases, BCPs were detected between the P atoms and the O atoms of the bridging pyO ligands; therefore, the weak P···O interactions are included in this discussion. The Ru–P bonds are similar to one another in terms of electron density *ρ* at the BCP. A slight decrease in this value is observed from compounds **3** via **4** to **5**, which is in accord with the trend of increasing bond length in this order. The ratio of the modulus of the potential energy density per Lagrangian kinetic energy density is in the range of 1 < |*V*(**r_b_**)|/*G*(**r_b_**) < 2 in all cases and is indicative of an intermediate bond characteristic (i.e., closed-shell covalent bond with additional ionic contribution). The Ru–P bonds do not exhibit any noticeable ellipticity *ε* of the electron density (≤0.1 in all cases). However, the Wiberg bond index (WBI) indicates certain multiple bond characteristics for compounds **3** (WBI ≈ 1.4–1.5), whereas a WBI close to 1 is shown for Ru–P single bonds in compounds **4** and **5**. In compounds **4** and **5**, the Ru–C bonds to CO or isonitrile, respectively, exhibit some multiple bond characteristics, with WBI values ranging between 1.4 and 1.7. As for Ru–P, the Ru–C bonds’ ellipticity of electron density is also < 0.1, indicating the radial symmetry of the bonding contributions. The π-donor *trans* to Ru–P in **3a** and **3b**, as well as the higher multiple bond character of Ru–CO in **4b** over Ru–CN*t*Bu in **5a** and **5b** in combination with the lower WBI of Ru–P in **4b**, indicate competing Ru→L π-acceptor ligand contributions as the origin of this variety of WBI values observed for compounds **3**, **4** and **5**.

The P–O bonds in compounds **3**, **4** and **5** can be divided into three groups. The very long P···O contacts (as found in pic-bridged Ru-P-complexes **3a** and **5a**) exhibit very low electron density values at the BCP (ca. 0.02 au), a ratio |*V*(**r_b_**)|/*G*(**r_b_**) very close to 1 and a WBI close to 0.1, supporting the interpretation of weak donor–acceptor interactions. Furthermore, the electron energy density *H*(**r_b_**) is close to zero, also supporting the absence of covalent bonding. In the context of their low total electron density, minor variations in electron density distribution already cause large effects on *ε*, while the values of *ε* > 0.3 encountered with these P···O contacts should not be interpreted as the results of multiple bonding. The second group of P-O-interactions corresponds to formally covalent P–O single bonds with a WBI close to 1. The short ones of this group (i.e., the apical P–O bond at the square–pyramidal-coordinated P atom in **3b** and the shorter P–O bonds of **5b**) exhibit electron densities in the range 0.14–0.16 au, a WBI slightly above 1 and a ratio |*V*(**r_b_**)|/*G*(**r_b_**) close to 1.5. The latter, as mentioned above, is characteristic of closed-shell covalent bonds with additional ionic contributions. The longer ones of this group, which are part of nearly symmetrical linear O–P–O-arrangements, exhibit WBI values slightly below 1, somewhat lower electron density values (in the range 0.10–0.12 au) and a ratio |*V*(**r_b_**)|/*G*(**r_b_**) closer to 2. Most of these bonds also exhibit noticeably enhanced ellipticity of their electron density at the BCP. The third “class” of P–O bonds encountered with these compounds is the pair of long P–O bonds in **5b**. Their features are intermediate between those of the two former groups: an electron density value of 0.05 au, 1.5 < |*V*(**r_b_**)|/*G*(**r_b_**) < 1 and WBI of 0.36. This intermediate situation of this set of P–O bonds, as detected by this topological analysis, is in accord with the intermediate situation found for the same bonds in the NCI analysis (cf. Figure 9).

#### 2.4.4. NBO-/NLMO-Analyses

**Figure 10:**
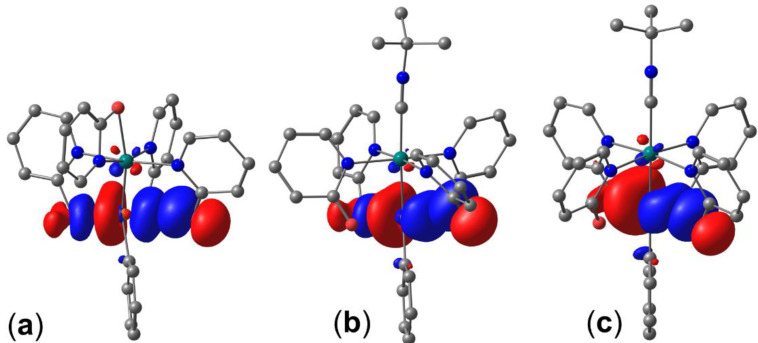
Visualization of NBOs (isosurface 0.05 au) involved in σ-O→P donor–acceptor interactions in (**a**) compound **3a**, (**b**) compound **5a** (for one of the two O→P interactions) and (**c**) compound **5b** (for one of the two O→P interactions). Hydrogen atoms are omitted for clarity (for further O→P interactions in **5a** and **5b**, see Figure S33 in the Supplementary Materials).

For a closer view of π-back-bonding contributions and weak donor–acceptor-interactions between pyO-ligands and P atoms, as well as for insights into the atomic contributions to the Ru–P σ-bond, we analyzed natural bond orbitals (NBOs) and natural localized molecular orbitals (NLMOs) of compounds **3a**, **3b**, **4b**, **5a** and **5b**. Table 2 lists the natural charges (NCs) of these compounds’ Ru- and P-atoms as well as these atoms’ contributions to the Ru–P σ-bond. The NCs of the Ru atoms are only slightly positive, and replacement of the Ru-bound O atom by a monodentate ligand (CO or CN*t*Bu) lowers the Ru atom´s NC by ca. 0.15. Even though an anionic π-donor ligand atom is replaced by a charge-neutral π-acceptor ligand, the different electronegativities (O vs. C) appear to dominate the effect on the NC. In accord, replacing two P-bound pyO moieties by pic moieties (i.e., replacement of P–O by P–C bonds) results in lowering of the P atom´s NC by ca. 0.4. In spite of the variable number of additional O-donor sites in its proximity, the P-atom´s NC is almost identical for the PhP(pyO)_n_Ru-compounds **3b**, **4b** and **5b**. For those compounds, which exhibit σ-O→P donor–acceptor interactions (**3a**, **5a**, **5b**), second-order perturbation theory analysis revealed the relevant orbital interactions (Figure 10) and the energies *E*(σ-O→P) listed in Table 2. The increasing intensity of those interaction energies (ca. 4, 6 and 18 kcal mol^−1^ for **5a**, **3a** and **5b**, respectively) resembles the increasing intensity indicated along this series in the NCI analyses (Figure 9).

The σ-Ru–P bonds, in most cases, can be interpreted as polar covalent bonds with ca. 2/3 phosphorus contributions. Comparison of the σ-Ru–P relevant NLMOs of compounds **3a** and **3b** shows that the exchange of bridging moieties (pic vs. pyO) has only a marginal influence on the Ru–P σ-bond. For compounds **4b** and **5a**, NBO/NLMO analyses initially afforded delocalized Ru–P σ-bonds (noticeable delocalization of the σ-Ru–P NLMO across the P-Ru-C axis, involving significant atomic orbital contributions of the *trans*-disposed ligand, CO or CN*t*Bu). This was not unexpected, as in a previous analysis we had already encountered such a delocalized situation with **4b** [4]. For the sake of comparability, in order to obtain corresponding NLMOs with predominant two-atom contributions in compounds **4b** and **5a** as well, the occupancy threshold of the Lewis structure search was adjusted (from an initial value of 1.65 to 1.57 or 1.51 for **4b** or **5a**, respectively). The NLMOs thus obtained revealed close similarities between **5a** and **5b**, underlining that pic- vs. pyO-exchange does not affect the σ-Ru–P bond significantly. (Graphical representations of the σ-Ru–P NLMOs can be found in the Supporting Information, Figure S34.) The composition of the σ-Ru–P NLMO of compound **4b** hints at a more covalent situation (equal orbital contributions from both atoms involved).

Replacing pyO- with pic-bridges, however, has significant influence on the Ru–P bond with respect to Ru→P π-back-donation. For compound **3b**, second-order perturbation theory analysis revealed a total of 30.4 kcal mol^−1^ Ru→P π-back-bonding energy with two major contributions of 20.2 and 7.8 kcal mol^−1^ into the P–O-based σ-antibonding orbitals and a minor contribution of 2.3 kcal mol^−1^ associated with σ*(P–C(Ph)). In compound **3a**, the energetically small back-bonding contributions into the σ*(P–C) NBOs result in a total of only 11.5 kcal mol^−1^. With respect to **3b**, the weaker Ru→P π-back-donation in **3a** is in accord with the lower WBI and the longer Ru–P bond found for **3a**. Introduction of *trans*-disposed π-acceptor ligands (CO or CN*t*Bu) lowers the Ru-P π-back-donation, as expected. Interestingly, in spite of the stronger π-acceptor CO, π-back-bonding to the phosphorus site is still more efficient in **4b** than in **5b**. We attribute this to the more symmetrical (nearly square-shaped) PO_4_ moiety of **4b**, the σ*-O-P-O orbitals of which serve as π-acceptors. This formal flow of electron density in the Ru→P direction in the π-system appears to be compensated for by enhanced σ-Ru←P donation. The contributions of P and Ru to the NLMO, which is representative of the σ-bond, indicate a shift of the electron pair toward Ru. (Graphical representations of the NBOs involved in π-Ru→C, and where applicable π-Ru→P interactions can be found in the Supporting Information, Figures S35–S37).

## 3. Materials and Methods

### 3.1. General Considerations

Starting materials [Ru(pyO)_2_(nbd)] **1** [4], **2a** [15] and **3b** [4] were prepared following the literature protocols. CN*t*Bu (Sigma-Aldrich, Steinheim, Germany, 98%) was used as received without further purification. CD_2_Cl_2_ (Deutero, Kastellaun, Germany, 99.6%), acetonitrile (Roth, Karlsruhe, Germany, >99.95%) and *n*-pentane (Th.Geyer, Renningen, Germany, >99%) were stored over activated molecular sieves (3 Å) for at least 7 days and used without further purification. Dichloromethane was distilled from calcium hydride, while diethyl ether and toluene were distilled from sodium benzophenone. All reactions were carried out under an atmosphere of dry argon utilizing standard Schlenk techniques. Solution NMR spectra (^1^H, ^13^C, ^31^P) were recorded on Bruker Avance III 500 MHz and Bruker Nanobay 400 MHz spectrometers. ^1^H and ^13^C chemical shifts are referenced to Me_4_Si (0 ppm) or to solvent signals of CHDCl_2_ (^1^H 5.32 ppm) and CD_2_Cl_2_ (^13^C 53.84 ppm) as internal references, ^31^P shifts are reported relative to 85% H_3_PO_4_ (0 ppm). For compound **3a**, ^1^H and ^1^H COSY as well as ^1^H,^13^C HMBC and HSQC spectra were recorded for signal assignment. ^31^P (CP/MAS) NMR spectra were recorded on a Bruker Avance 400 WB spectrometer with 2.5 mm zirconia (ZrO_2_) rotors at an MAS frequency of *υ*_spin_ = 15 kHz (**3a**) or 10 kHz (**4a**·(toluene)). Infrared spectra of **4a**, **5a** and **5b** were recorded on a Nicolet 380 FT-IR instrument in ATR mode. Elemental analyses were performed on an Elementar Vario MICRO cube. For single-crystal X-ray diffraction analyses, crystals were selected under an inert oil and mounted on a glass capillary (which was coated with silicone grease). Diffraction data were collected on a Stoe IPDS-2/2T diffractometer (STOE, Darmstadt, Germany) using Mo Kα-radiation. Data integration and absorption correction were performed with the STOE software programs XArea and XShape, respectively. The structures were solved by direct methods using SHELXS-97 or SHELXT and refined with the full-matrix least-squares methods of *F*^2^ against all reflections with SHELXL-2014/7 or SHELXL-2018/3 [22,23,24,25,26]. All non-hydrogen atoms were anisotropically refined. Hydrogen atoms were isotropically refined in idealized position (riding model). For details on the data collection and refinement (incl. the use of SQUEEZE in the refinement of the structures of **4a** and **6a**), see Appendix A. Graphics of molecular structures were generated with ORTEP-3 [27,28] and POV-Ray 3.7 [29]. CCDC 2162022 (**3a**), 2162023 (**4a**·(toluene)), 2162024 (**5a**·1.5(CH_2_Cl_2_)), 2162025 (**6a**·1.5(CH_2_Cl_2_)) and 2162026 (**5b**) contain the supplementary crystal data for this article. These data can be obtained free of charge from the Cambridge Crystallographic Data Centre via https://www.ccdc.cam.ac.uk/structures/ (accessed on 14 April 2022).

The geometry optimizations were carried out with ORCA 5.0.2 [30] using the restricted PBE0 functional with a relativistically recontracted Karlsruhe basis sets ZORA-def2-TZVPP [31,32] (for H, C, N, O, P) and SARC-ZORA-TZVPP (for Ru) [33], the scalar relativistic ZORA Hamiltonian [34,35], atom-pairwise dispersion correction with the Becke–Johnson damping scheme (D3BJ) [36,37] and COSMO solvation (toluene, *ε* = 2.38, *r*_solv_ = 3.48). VeryTightSCF and slowconv options were applied and the DEFGRID3 was used with a radial integration accuracy of 10 for ruthenium for all calculations. Calculations were started from the molecular structures obtained by single-crystal X-ray diffraction analysis and isomers were created by modifying these structures. Numerical frequency calculations were performed to prove convergence at the local minimum after geometry optimization and to obtain the Gibbs free energy (293.15 K). The calculated C≡N stretching vibrations were taken from the numerical frequency calculations. On the final structures, single-point calculations were performed with a restricted B2T-PLYP functional with relativistically recontracted Karlsruhe basis sets ZORA-def2-TZVPP [31,32] (for H, C, N, O, P) and SARC-ZORA-TZVPP (for Ru) [33] and utilizing the AutoAux generation procedure [38], the scalar relativistic ZORA Hamiltonian [34,35], atom-pairwise dispersion correction with the Becke–Johnson damping scheme (D3BJ) [36,37] and COSMO solvation (toluene).

After optimization of the H-atom positions of the molecular structures obtained by single-crystal X-ray diffraction analyses, NBO and NLMO calculations were performed using ORCA 5.0.2 [30] with the NBO7.0 package [39] using the restricted PBE0 functional with relativistically recontracted Karlsruhe basis sets ZORA-def2-TZVPP [31,32] (for H, C, N, O, P) and SARC-ZORA-TZVPP (for Ru) [33], the scalar relativistic ZORA Hamiltonian [34,35], atom-pairwise dispersion correction with the Becke–Johnson damping scheme (D3BJ) [36,37] and COSMO solvation (toluene). QTAIM (quantum theory of atoms-in-molecules) [40], WBI [41] and NCI [42] calculations were performed with MultiWFN [43] at the same level of theory as used for NBO analysis. NBO/NLMO graphics were generated using Chemcraft [44] and visualization of the NCI results was carried out with VMD [45].

### 3.2. Syntheses and Characterization

Compound **3a** ([PhP(μ-pic)_2_(μ-pyO)Ru(κ^2^-pyO)], C_28_H_25_N_4_O_2_PRu). A Schlenk flask was charged with magnetic stirring bar, [Ru(pyO)_2_(nbd)] (**1**) (0.525 g, 1.50 mmol) and PhP(pic)_2_ (**2a**) (0.440 g, 1.50 mmol), evacuated and set under Ar atmosphere prior to adding acetonitrile (10 mL). The resultant dispersion was heated and stirred under reflux. Within the first ten minutes of heating, the color of the dispersion changed from yellow to orange. Upon 3 h of heating, the mixture was allowed to attain room temperature. The solid product was filtered off, washed with acetonitrile (2 × 3 mL) and dried in vacuum. (Crystals suitable for X-ray diffraction analysis were grown from a dichloromethane solution of this product upon diffusion of diethyl ether via gas phase in the course of one week.) Yield: 0.589 g (1.01 mmol, 68%). Elemental analysis for C_28_H_25_N_4_O_2_PRu (581.56 g·mol^−1^): C, 57.83%; H, 4.33%; N, 9.63%; found C, 57.64%; H, 4.33%; N, 9.32%. ^1^H NMR (CD_2_Cl_2_): *δ* (ppm) 9.01 (d, 2H, pic-6, 5.3 Hz), 7.83 (br, 2H, pyO-6), 7.78 (ddd, 2H, Ph-ortho, 1.5 Hz, 7.6 Hz, 11.7 Hz), 7.42–7.48 (m, 3H, Ph-meta/para), 7.32 (t, 2H, pic-4, 7.6 Hz), 7.24 (d, 2H, pic-3, 7.6 Hz), 7.07 (m, 2H, pyO-4), 6.89 (“t”-shaped dd, 2H, pic-5, 5.3 Hz, 7.6 Hz), 5.95–6.05 (m, 4H, pyO-5,3), 3.96 (br, 2H, CH_2_), 3.65 (dd, 2H, CH_2_, 17.0 Hz, 10.8 Hz); ^13^C{^1^H} NMR (CD_2_Cl_2_): *δ* (ppm) 173.7 (pyO-2), 164.8 (pic-2), 152.6 (pic-6), 150.6 (pyO-6), 137.4 (d, Ph-ipso, 60 Hz), 136.2 (pyO-4), 134.2 (pic-4), 130.6 (d, Ph-ortho, 11 Hz), 130.0 (d, Ph-para, 2 Hz), 128.6 (d, 11 Hz, Ph-meta), 122.1 (d, 11 Hz, pic-3), 121.7 (pic-5), 112.0 (pyO-3), 107.7 (pyO-5), 46.2 (CH_2_); ^31^P{^1^H} NMR (CD_2_Cl_2_): *δ* (ppm) 80.8; ^31^P CP/MAS NMR: *δ*_iso_ (ppm) 73.8, 76.3.

Compound **4a** ([PhP(μ-pic)_2_(μ-pyO)Ru(κ-*O*-pyO)(CO)], C_29_H_25_N_4_O_3_PRu) and compound **6a** ([PhP(μ-pic)_2_(μ-pyO)Ru(Cl)(CO)], C_24_H_21_ClN_4_O_3_PRu). A Schlenk flask (volume ca. 15 mL) charged with magnetic stirring bar, compound **3a** (0.116 g, 0.199 mmol) and toluene (2.5 mL) was cooled in liquid N_2_ prior to evacuating the initial atmosphere and recharging with CO atmosphere in 3 cycles. (The gas volume in the Schlenk flask (>10 mL) corresponds to excess CO (>0.45 mmol).) Then, the contents were allowed to attain room temperature, and the resultant orange dispersion was stirred at room temperature for two days. On the third day, the contents were stirred at 60 °C for 6 h (the contents remained a dispersion) and then stored at room temperature for 3 days. (Some crystals suitable for X-ray diffraction analysis were taken from the crude product.) Thereafter, the contents were separated from the supernatant by decantation, washed with toluene (1.5 mL) and briefly dried in vacuum. Yield: 0.05 g (0.07 mmol, ca. 35%) of **4a**·(toluene). ^31^P NMR spectroscopy of both the solid and CD_2_Cl_2_ solution of this product indicated the presence of some starting material (contains ca. 15% **3a**). Therefore, elemental analysis data are not reported. An attempt at recrystallizing crude **4a**·(toluene) from dichloromethane (with gas phase diffusion of *n*-pentane) afforded some crystals of complex **6a**. (The formation of **6a** may originate from traces of HCl contained in dichloromethane. Nonetheless, even though pyridine itself is not sufficiently nucleophilic to undergo nucleophilic substitution of chloride from DCM under mild conditions [46], metal-bound pyridyl groups have been shown to undergo nucleophilic attack at DCM [47,48]. Thus, the latter path cannot be ruled out at the current stage. However, side-products of the formation of **6a** were not identified.) Analogous synthesis of stirring a dispersion of **3a** (0.095 g, 0.16 mmol) in acetonitrile (2 mL) under an atmosphere of CO afforded a clear solution within one day, and the solution remained clear for one week. Gas-phase diffusion of diethyl ether did not result in crystallization of the target product, and a ^31^P NMR spectrum recorded from this crude solution (products in MeCN/Et_2_O) indicated the presence of both **4a** and starting material **3a** with signals at 68.9 and 80.2 ppm, respectively, at an intensity ratio of 2:1. The limited amount of sample available (especially in case of **6a**) and decomposition/reaction with solvent (in case of **4a**), 1D ^13^C{^1^H} and 2D ^13^C NMR spectroscopy did not allow for detecting all ^13^C signals or assignment of all signals observed. Thus, the ^13^C NMR shifts reported here basically serve as fingerprints of **4a** and **6a**.

NMR data for **4a** (recorded from the crude product of **4a**·(toluene)): ^1^H NMR (CD_2_Cl_2_): *δ* (ppm) 9.71 (dd, 1H, 5.7 Hz, 1.6 Hz), 8.82 (dd, 1H, 5.8 Hz, 1.6 Hz), 8.02–8.06 (m, 2H), 7.75 (m, 2H, Ph-ortho), 7.62 (m, 1H), 7.37–7.56 (m, 7H), 7.30–7.35 (m, 2H), 6.86–6.97 (m, 2H), 6.46 (ddd, 1H, 8.6 Hz, 5.0 Hz, 1.0 Hz), 5.86 (“dt”-like m, 1H, 6.4 Hz, 1.5 Hz, 1.0 Hz), 5.68 (dd, 1H, 8.6 Hz, 1.0 Hz), 4.79 (dd, 1H, CH_2_, 17.4 Hz, 13.8 Hz), 3.87 (dd, 1H, CH_2_, 16.7 Hz, 12.2 Hz), 3.64 (dd, 1H, CH_2_, 17.4 Hz, 8.4 Hz), 3.50 (dd, 1H, CH_2_, 16.7 Hz, 7.2 Hz); ^13^C{^1^H} NMR (CD_2_Cl_2_): *δ* (ppm) 172.5, 169.9, 162.3, 153.0, 152.8, 150.0, 148.4, 137.7, 137.4 (2×), 136.7, 131.2 (d, 11 Hz), 131.0, 128.9 (d, 12 Hz), 123.0, 122.6, 115.2, 114.3, 110.5, 105.9, 41.5, 41.1; ^31^P{^1^H} NMR (CD_2_Cl_2_): *δ* (ppm) 68.6; ^31^P CP/MAS NMR: *δ*_iso_ (ppm) 63.5, 68.1.

NMR data for **6a**: ^1^H NMR (CD_2_Cl_2_): *δ* (ppm) 9.74 (ddd, 1H, pic-6, 5.9 Hz, 1.6 Hz, 0.7 Hz), 9.67 (ddd, 1H, pic’-6, 5.8 Hz, 1.7 Hz, 0.8 Hz), 8.64 (dd, 1H, pyO-6, 6.4 Hz, 2.1 Hz), 7.40–7.70 (m, 8H, pic-5, pic’-5, pic’-3, Ph-ortho/-meta/-para), 7.31 (ddd, 1H, pic-3, 7.9 Hz, 1.2 Hz, 0.7 Hz), 7.17 (“tt”-like m, 1H, pic-4, 6.6 Hz), 7.10 (“tt”-like m, 1H, pic’-4, 7.5 Hz, 6.7 Hz), 6.95 (ddd, 1H, pyO-4, 8.6 Hz, 6.4 Hz, 2.1 Hz), 6.00 (dt, 1H, pyO-5, 2 × 6.4 Hz, 1.5 Hz), 5.64 (dddd, 1H, pyO-3, 8.6 Hz, 1.5 Hz, 2 × 0.5 Hz), 4.80 (dd, 1H, CH_2_, 17.3 Hz, 14.6 Hz), 3.84 (dd, 1H, CH_2_, 16.8 Hz, 12.8 Hz), 3.60 (dd, 1H, CH_2_, 17.3 Hz, 7.3 Hz), 3.46 (dd, 1H, CH_2_, 16.8 Hz, 7.2 Hz); ^13^C{^1^H} NMR (CD_2_Cl_2_): *δ* (ppm) 155.3, 153.9, 150.7, 137.9, 137.1, 131.1, 130.3 (d, 11 Hz), 129.1 (d, 12 Hz), 123.8, 123.4, 122.7, 114.3, 106.1; ^31^P{^1^H} NMR (CD_2_Cl_2_): *δ* (ppm) 66.7.

Compound **5a** ([PhP(μ-pic)_2_(μ-pyO)_2_Ru(CN*t*Bu)], C_33_H_34_N_5_O_2_PRu). To a solution of compound **3a** (0.062 g, 0.107 mmol) in dichloromethane (2 mL), CN*t*Bu (0.011 g, 0.132 mmol) was added, whereupon the initially deep orange solution changed color to lighter orange. Thereafter, the volume of the solution was reduced to 0.5 mL (by condensation of parts of the solvent into a cold trap under reduced pressure), and the flask with the crude solution of **5a** was connected to a second flask with 3 mL of *n*-pentane (for gas phase diffusion). Within one day, some crystals of **5a**·1.5(CH_2_Cl_2_) had formed. One of the crystals was used for single-crystal X-ray diffraction analysis, while the remaining few crystals were separated from the supernatant by decantation and briefly dried in vacuum. The ^1^H NMR spectrum of this crude product (cf. Figure 6) indicated the presence of two isomers of **5a** (**5a^1^** and **5a^3^**), as well as of starting materials **3a** and CN*t*Bu. The latter two are likely to have formed from **5a** in a dissociation reaction. Elemental analysis data correspond very well to the composition of **5a**·(CH_2_Cl_2_): Elemental analysis for C_34_H_36_Cl_2_N_5_O_2_PRu (749.63 g·mol^−1^): C, 54.48%; H, 4.84%; N, 9.34%; found C, 54.55%; H, 5.14%; N, 9.57%. Therefore, for NMR spectroscopic characterization a solution of **5a** (isomer **5a^3^** initially formed) in CD_2_Cl_2_ was prepared in situ and NMR spectra were recorded within few hours. ^1^H signals were assigned by considering shift ranges (of pic from **3a** and pyO from **5b**) and coupling patterns. Caution: Some couplings were not resolved, meaning the coupling constants reported here represent the superposition of two couplings of similar frequency. ^1^H NMR (CD_2_Cl_2_): *δ* (ppm) 9.25 (dd, 2H, pyO-6, 5.9 Hz, 1.1 Hz), 7.97 (m, 2 H, Ph-ortho), 7.50 (dd, 2H, pic-6, 6.2 Hz, 2.2 Hz), 7.40–7.45 (m, 3H, Ph-meta/para), 7.36 (tt, 2H, pic-4, 7.6 Hz, 1.1 Hz), 7.17 (d, 2H, pic-3, 7.6 Hz), 6.88 (ddd, 2H, pyO-4, 8.6 Hz, 6.5 Hz, 2.3 Hz), 6.83 (“dt-like” m, 2H, pic-5, 7.5 Hz, 6.3 Hz, 1.3 Hz), 5.81 (dd, 2H, pyO-3, 8.6 Hz, 1.5 Hz), 5.62 (m, 2H, pyO-5, 1.5 Hz), 4.03 (dd, 2H, CH_2_, 16.9 Hz, 8.6 Hz), 3.52 (dd, 2H, CH_2_, 16.9 Hz, 10.5 Hz); ^13^C{^1^H} NMR (CD_2_Cl_2_): *δ* (ppm) 172.5, 164.3 (d, 8 Hz), 157.1, 154.7 (d, 4 Hz), 135.7, 135.1, 133.5 (d, 9 Hz), 129.9 (d, 2 Hz), 128.4 (d, 9 Hz), 122.8 (d, 10 Hz), 121.6, 114.8, 105.1, 57.6 (*C*Me_3_), 42.2 (d, CH_2_, 23 Hz), 31.2 (CH_3_) (The signals of Ph-ipso-*C* and *C*NC*t*Bu have not been detected.); ^31^P{^1^H} NMR (CD_2_Cl_2_): *δ* (ppm) 40.1.

Compound **5b** ([PhP(μ-pyO)_4_Ru(CN*t*Bu)], C_31_H_30_N_5_O_4_PRu). To a dispersion of compound **3b** (0.113 g, 0.193 mmol) in toluene (7 mL), which was stirred at room temperature, CN*t*Bu (0.0184 g, 0.22 mmol) was added. Thereafter, the dispersion was stirred at 90 °C for 2.5 h and then stored at room temperature overnight, whereupon the yellow solid was filtered off, washed with toluene (2 mL) and dried in vacuum. Yield: 0.068 g (0.102 mmol, 53%). (Some crystals suitable for X-ray diffraction analysis formed upon gas phase diffusion of *n*-pentane into the combined filtrate and washings.) Elemental analysis for C_31_H_30_N_5_O_4_PRu (668.64 g·mol^−1^): C, 55.77%; H, 4.56%; N, 10.46%; found C, 55.67%; H, 4.52%; N, 10.47%. ^1^H NMR (CD_2_Cl_2_): *δ* (ppm) 8.61 (dd, 4H, pyO-6, 6.0 Hz, 2.0 Hz), 8.45 (m, 2H, Ph-ortho), 7.35-7.45 (m, 3H, Ph-meta/para), 7.23 (ddd, 4H, pyO-4, 8.4 Hz, 6.9 Hz, 2.0 Hz), 6.51 (dd, 4H, pyO-3, 8.4 Hz, 0.9 Hz), 6.32 (ddd, 4H, pyO-5, 6.9 Hz, 6.0 Hz, 1.4 Hz), 1.90 (s, 9H, CH_3_); ^13^C{^1^H} NMR (CD_2_Cl_2_): *δ* (ppm) 165.7 (d, pyO-2, 5.4 Hz), 152.4 (d, Ph-ipso, 225 Hz), 152.3 (d, pyO-6, 3.9 Hz), 138.0 (pyO-4), 134.3 (d, Ph-ortho, 14 Hz), 127.8 (d, Ph-para, 4 Hz), 126.1 (d, Ph-meta, 17 Hz), 113.1 (pyO-5), 111.1 (d, pyO-3, 3 Hz), 57.7 (*C*Me_3_), 31.7 (CH_3_); ^31^P{^1^H} NMR (CD_2_Cl_2_): *δ* (ppm) 28.0.

## 4. Conclusions

In this study, we have shown that 2-picolyl (pic) moieties may be employed as bridging entities between P and Ru atoms in complexes, in which a hemilabile Ru-bound ligand (in our case a chelating 2-pyridyloxy group, pyO) can undergo chelate opening and take advantage of stabilization of the dangling ligator function by binding to the adjacent P atom. Whereas the pic groups (in the starting material PhP(pic)_2_ and in the Ru complexes resulting therefrom) imply the advantage of a more robust building block with respect to lowered hydrolytic sensitivity compared with related pyO-based systems (PhP(pyO)_2_ and in the Ru complexes resulting therefrom), they lower the Lewis acidity of the P atom. This affects both the nature of the P–Ru bond (which features significantly lowered Ru→P π-back-bonding contributions) and the tendency for binding of the dangling ligand arm to the P atom. Thus, in PhP(pic)_2_-based systems (**3a**, **4a**, **5a**), the latter is noticeably less pronounced than in the related PhP(pyO)_2_-based Ru complexes (**3b**, **4b**, **5b**). This fosters reactions back toward formation of the Ru(κ^2^-pyO)-chelate (with release of monodentate ligands in equilibrium, such as Ru-bound isonitrile) or even Ru(κ^2^-pyO)-chelate opening with formation of Ru(κ-*O*-pyO)-complexes, which feature a dangling pyO nitrogen atom. The dangling N atom of the latter may cause unwanted side reactions (e.g., reaction of **4a** and dichloromethane or traces of HCl contained therein with formation of **6a**).

**Scheme 3:**
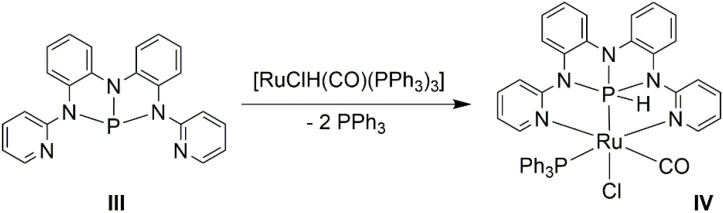
Hydride migration to a phosphane ligand [46].

In summary, further exploration of related kinds of coordinative switches within Ru-P-systems may benefit from electronegative substituents at the P atom. In general, the subject matter of ligand migration from Ru to an adjacent P atom is worth exploring further. In a recent study, Tanushi and Radosevich showed the migration of an Ru-bound hydride to a special phosphane ligand **III** (which also bears pyridine anchors as Ru-binding site) with the formation of complex **IV** (Scheme 3) [49]. This hints at the greater potential of such systems for stabilizing monodentate ligands with an Ru-bound phosphane P atom.

From an academic point of view, the herein presented compound **5b** represents a rare example of a monometallic phosphane complex with hexacoordination of both the transition metal and the phosphorus atom.

## Figures and Tables

**Table 1:** Selected features of (3, −1) critical points in compounds **3a**, **3b**, **4a**, **5a** and **5b.** Note: electron density *ρ*(**r_b_**) in au, Laplacian of the electron density ∇^2^*ρ*(**r_b_**) in au, Lagrangian kinetic energy density *G*(**r_b_**) in au, potential energy density *V*(**r_b_**) in au, ratios |*V*(**r_b_**)|/*G*(**r_b_**) and *G*(**r_b_**)/*ρ*(**r_b_**) in au, electron energy density *H*(**r_b_**) in au, ellipticity of the electron density *ε* and Wiberg Bond Index (WBI).

Compd	Bond ^1^	*ρ*(r_b_)	∇^2^*ρ*(r_b_)	*G*(r_b_)	*V*(r_b_)	|*V*(r_b_)|/*G*(r_b_)	*H*(r_b_)	*G*(r_b_)/*ρ*(r_b_)	*ε*	WBI
**3a**	Ru–P	0.1264	0.1395	0.0968	−0.1588	1.640	−0.0620	0.766	0.099	1.407
	P···O	0.0233	0.0588	0.0148	−0.0149	1.005	−0.0001	0.635	0.344	0.126
**3b**	Ru–P	0.1393	0.1368	0.1086	−0.1830	1.685	−0.0744	0.780	0.099	1.491
	P–O ^2^	0.1558	0.4284	0.2222	−0.3372	1.518	−0.1151	1.426	0.045	1.167
	P–O	0.1088	0.0343	0.0911	−0.1735	1.906	−0.0825	0.837	0.248	0.854
	P–O	0.0960	−0.0240	0.0614	−0.1287	2.098	−0.0674	0.639	0.244	0.734
**4b**	Ru–P	0.1137	0.0844	0.0697	−0.1183	1.697	−0.0486	0.613	0.011	0.945
	Ru–C	0.1417	0.5397	0.1980	−0.2610	1.318	−0.0630	1.397	0.042	1.716
	P–O	0.1179	0.0878	0.1124	−0.2029	1.805	−0.0905	0.953	0.350	0.918
	P–O	0.1160	0.0671	0.1061	−0.1955	1.842	−0.0894	0.915	0.345	0.900
	P–O	0.1094	0.0093	0.0860	−0.1697	1.973	−0.0837	0.786	0.383	0.841
	P–O	0.1052	−0.0170	0.0748	−0.1539	2.057	−0.0791	0.711	0.412	0.813
**5a**	Ru–P	0.1018	0.1566	0.0792	−0.1192	1.506	−0.0400	0.777	0.040	1.034
	Ru–C	0.1331	0.4778	0.1741	−0.2287	1.314	−0.0546	1.308	0.061	1.424
	P···O	0.0218	0.0556	0.018	−0.0136	0.989	−0.0002	0.632	0.277	0.109
	P···O	0.0197	0.0528	0.0127	−0.0122	0.959	−0.0005	0.645	0.419	0.093
**5b**	Ru–P	0.1130	0.1581	0.0876	−0.1357	1.549	−0.0481	0.776	0.006	1.107
	Ru–C	0.1216	0.4633	0.1603	−0.2048	1.278	−0.0445	1.319	0.053	1.351
	P–O ^3,4^	0.1441	0.3535	0.1943	−0.3002	1.545	−0.1059	1.348	0.110	1.110
	P···O ^4^	0.0503	0.0652	0.0280	−0.0397	1.418	−0.0117	0.557	0.229	0.359

^1^ P–O bonds (from top to bottom for each compound) are in the order of increasing interatomic separation. ^2^ This P–O bond occupies an apical position in the square-pyramidal P coordination sphere and is noticeably shorter than the other two P–O bonds in the same molecule (1.68 vs. 1.88 and 1.95 Å) [4]. ^3^ This set of P–O bonds in **5b** is noticeably shorter than the P–O bonds in compound **4b** (1.71 vs. 1.83–1.90 Å) [4]. ^4^ Because of the symmetry of the molecule (cf. Figure 5b), it features two pairs of chemically equivalent P-O-interactions. Redundant data have been omitted from this table.

**Table 2:** Natural charges (NCs) of Ru- and P-atoms and contributions to the NLMO of the Ru–P σ-bonds of compounds **3a**, **3b**, **4b**, **5a** and **5b**, as well as energy levels of selected intramolecular donor–acceptor interactions (obtained from second-order perturbation theory) in kcal mol^−1^ (∑*E*(π-Ru→P) = sum of π-back-bonding contributions into relevant σ-antibonding P–O- or P–C-based orbitals (∑*E*(π-Ru→C) = sum of π-back-bonding contributions into relevant π-antibonding C–O- or C–N-based orbitals of the CO or CN*t*Bu ligand).

	3a	3b ^1^	4b ^1^	5a	5b
NC(Ru)	0.229	0.181	0.032	0.080	0.036
NC(P)	1.335	1.748	1.730	1.263	1.741
NLMO σ-Ru–P	36.7% Ru	36.2% Ru	45.9% Ru	36.6% Ru	35.1% Ru
	61.0% P	61.5% P	52.0% P	60.9% P	62.0% P
*E*(σ-O→P)	5.6	-	-	3.4, 3.7	17.8, 17.8
∑*E*(π-Ru→P)	11.5	30.4	13.4	4.5	7.3
∑*E*(π-Ru→C)	-	-	53.3	40.0	36.1

^1^ Even though NLMO analyses had been performed for **3b** and **4b** previously [4], we repeated the calculations with the method–basis set combination used in the current paper for the sake of comparability.

## Data Availability

Not applicable.

## Supplementary Materials

The following Supporting Information can be downloaded at: https://www.mdpi.com/article/10.3390/molecules27092778/s1. Crystallographic data for the compounds reported in this paper (in CIF format) and a document containing graphics of the ^1^H, ^13^C and ^31^P NMR spectra of compounds **3a**, **4a**, **5a**, **5b** and **6a**; data sets (consisting of molecular graphic, atomic coordinates and total energies) of the optimized molecular structures of **4a^1^**, **4a^2^**, **4a^3^**, **4a^4^**, **4a^5^**, **4a^6^**, **4a^7^**, **5a^1^**, **5a^3^**, **5b^1^**, **5b^3^**, **4b^1^** and **4b^3^**; graphics of selected NBOs and NLMOs of compounds **3a**, **3b**, **4b**, **5a** and **5b**.

## Sample Availability

The compounds reported in this paper were prepared in small quantities only. Thus, no samples are available from the authors.

## Appendix A

**Table A1:** Crystallographic data from data collection and refinement processes for **3a**, **4a**·(toluene), **5a**·1.5(CH_2_Cl_2_)_,_ **5b** and **6a**·1.5(CH_2_Cl_2_).

Parameter	3a ^1^	4a·(toluene) ^2^	5a·1.5(CH_2_Cl_2_) ^3^	5b	6a·1.5(CH_2_Cl_2_) ^4^
Formula	C_28_H_25_N_4_O_2_PRu	C_36_H_33_N_4_O_3_PRu	C_34.5_H_37_Cl_3_N_5_O_2_PRu	C_31_H_30_N_5_O_4_PRu	C_25.5_H_24_Cl_4_N_3_O_2_PRu
*M*_r_	581.56	701.70	792.08	668.64	678.32
*T*(K)	130(2)	180(2)	180(2)	150(2)	200(2)
*λ*(Å)	0.71073	0.71073	0.71073	0.71073	0.71073
Crystal system	orthorhombic	monoclinic	triclinic	orthorhombic	monoclinic
Space group	*Pna*2_1_	*P*2_1_/*c*	*P* $\bar{1}$	*Pnma*	*P*2_1_/*n*
*a*(Å)	13.6790(5)	32.2445(9)	9.4331(2)	21.4034(9)	13.2082(5)
*b*(Å)	22.5495(7)	26.2658(8)	14.6220(4)	15.1338(7)	12.7594(3)
*c*(Å)	15.7935(5)	15.8729(4)	15.1733(4)	8.9456(3)	17.3007(6)
*α*(°)	90	90	66.690(2)	90	90
*β*(°)	90	103.220(2)	75.171(2)	90	97.635(3)
*γ*(°)	90	90	71.763(2)	90	90
*V*(Å^3^)	4871.6(3)	13086.9(6)	1804.44(9)	2897.6(2)	2889.82(16)
*Z*	8	16	2	4	4
*ρ*_calc_ (g·cm^−1^)	1.59	1.43	1.46	1.53	1.56
*μ*_MoKα_ (mm^−1^)	0.7	0.6	0.7	0.6	1.0
*F(000)*	2368	5760	810	1368	1364
*θ*_max_ (°), *R*_int_	28.0, 0.0771	25.0, 0.0923	28.0, 0.0277	25.0, 0.0906	27.0, 0.0381
Completeness	99.9%	99.9%	99.9%	100%	100%
Reflns collected	77123	119673	39878	14525	41367
Reflns unique	11761	13011	8731	2656	6315
Restraints	1	262	71	0	0
Parameters	650	1499	497	209	316
GoF	1.018	1.018	1.069	1.078	1.081
*R*1, *wR*2 [*I* > 2*σ*(*I*)]	0.0342, 0.0645	0.0515, 0.1101	0.0285, 0.0713	0.0406, 0.0824	0.0384, 0.0832
*R*1, *wR*2 (all data)	0.0568, 0.0705	0.1011, 0.1246	0.0315, 0.0729	0.0679, 0.0905	0.0468, 0.0865
Largest peak/hole (e·Å^−3^)	0.62, −0.62	0.74, −0.48	0.49, −0.73	0.44, −0.67	0.86, −0.66

^1^ The structure of compound **3a** was refined as an inversion twin. Without taking the twin into account, the absolute structure parameter χ_Flack_ is 0.30(3). ^2^ The asymmetric unit comprises four toluene molecules, which suffer heavy disorder. Therefore, the solvent was not refined but treated with SQUEEZE as implemented in PLATON [50,51,52]. This procedure detected, per unit cell, a solvent-accessible volume of 3090 Å^3^ and contributions of 840 electrons therein (close to the 800 electrons for the 16 toluene molecules per unit cell, which were omitted from refinement). ^3^ The asymmetric unit comprises 1.5 CH_2_Cl_2_ molecules. One molecule is disordered over three positions and was refined with site occupancies of 0.440(3), 0.234(3) and 0.326(3). The other solvent site is near a crystallographically imposed center of inversion (0.5 molecules per asymmetric unit). In addition to the symmetry-related disorder in this position, this half molecule was refined in two individual orientations with site occupancy ratio 0.767(5):0.233(5). ^4^ The asymmetric unit comprises 1.5 CH_2_Cl_2_ molecules. One molecule is well ordered and was refined. The other solvent site is near a crystallographically imposed center of inversion (thus 0.5 molecules per asymmetric unit), and this half molecule suffers heavy disorder. Therefore, this part of the solvent was not refined but treated with SQUEEZE as implemented in PLATON [50,51,52]. This procedure detected, per unit cell, solvent accessible volume of 355 Å^3^ and contributions of 83 electrons therein (well in accord with 84 electrons for the two CH_2_Cl_2_ molecules per unit cell, which have been omitted from refinement).

## Appendix B

In the IR spectra, compound **5a** exhibits two strong bands characteristic of C≡N stretching vibrations at 2091 and 2052 cm^−1^. In the same region, compound **5b** exhibits only one band, at 2087 cm^−1^. This hints at the presence of two isomers in this solid product of **5a** and one isomer of **5b** (which is in accord with ^1^H NMR data). Thus, we attribute the bands at 2052 and 2087 cm^−1^ to the isomers **5a^3^** and **5b^3^**, respectively, and the band at 2091 cm^−1^ to isomer **5a^1^**. This assignment is based on the C≡N of **5a^1^** resonating at somewhat higher wave numbers than **5b^3^** (the trend found for the C≡O stretch of complexes **4a^1^** and **4b^3^**), while the lower wave number of the C≡N stretch of **5a^3^** would be in accord with the stronger π-back-bonding, which is indicated by the shorter Ru–C bond in **5a** (vs. **5b**). Furthermore, this assignment is supported by computational analyses, which predict a C≡N stretch at enhanced wave numbers (+32 cm^−1^) for **5a^1^** with respect to **5a^3^** (cf. Section 2.4, optimized molecular structures of selected isomers). In general, the charge-neutral Ru(II) compounds **5** exhibit pronounced π-back-bonding to the CN*t*Bu ligand. For comparison, Ru(II)-compound [Ru(tp)Cl(PPh_3_)(CNtBu)] (tp = tris(pyrazol-1-yl)borate), which also bears good donor ligands at Ru(II), still exhibits slightly weaker back-bonding, indicated by a C≡N stretching vibration at 2117 cm^−1^ [53].
