# Supplementary material for: P–Ru-Complexes with a Chelate-Bridge-Switch: A Comparison of 2-Picolyl and 2-Pyridyloxy Moieties as Bridging Ligands
Source: Molecules. 2022 Apr 27;27(9):2778. doi: 10.3390/molecules27092778 (PMC9103559; doi:10.3390/molecules27092778)

checkCIF/PLATON report

Structure factors have been supplied for datablock(s) compound_3a, compound_4a, compound_5a, compound_5b, compound_6a

THIS REPORT IS FOR GUIDANCE ONLY. IF USED AS PART OF A REVIEW PROCEDURE FOR PUBLICATION, IT SHOULD NOT REPLACE THE EXPERTISE OF AN EXPERIENCED CRYSTALLOGRAPHIC REFEREE.

No syntax errors found. CIF dictionary Interpreting this report

Datablock: compound_3a

```
Bond precision:   C-C = 0.0095 A          Wavelength=0.71073

Cell:              a=13.6790(5)      b=22.5495(7)      c=15.7935(5)
                   alpha=90          beta=90           gamma=90
Temperature:       130 K

                 Calculated                   Reported
Volume           4871.6(3)                    4871.6(3)
Space group      P n a 21                     P n a 21
Hall group       P 2c -2n                     P 2c -2n
Moiety formula   C28 H25 N4 O2 P Ru           C28 H25 N4 O2 P Ru
Sum formula      C28 H25 N4 O2 P Ru           C28 H25 N4 O2 P Ru
Mr               581.56                       581.56
Dx,g cm-3        1.586                        1.586
Z                8                            8
Mu (mm-1)        0.744                        0.744
F000             2368.0                       2368.0
F000'            2359.45
h,k,lmax         18,29,20                     18,29,20
Nref             11767[ 6094]                 11761
Tmin,Tmax        0.901,0.956                  0.894,0.948
Tmin'            0.901

Correction method= # Reported T Limits: Tmin=0.894 Tmax=0.948
AbsCorr = INTEGRATION

Data completeness= 1.93/1.00       Theta(max)= 27.999

                                                  wR2(reflections)=
R(reflections)= 0.0342( 9028)                     0.0705( 11761)

S = 1.018                  Npar= 650
```

The following ALERTS were generated. Each ALERT has the format
test-name_ALERT_alert-type_alert-level.
Click on the hyperlinks for more details of the test.

Alert level C

```
PLAT342_ALERT_3_C Low Bond Precision on  C-C Bonds ...............    0.00946 Ang.
PLAT910_ALERT_3_C Missing # of FCF Reflection(s) Below Theta(Min).          5 Note
```

Alert level G

```
PLAT111_ALERT_2_G ADDSYM Detects New (Pseudo) Centre of Symmetry .         83 %Fit
PLAT113_ALERT_2_G ADDSYM Suggests Possible Pseudo/New  Space Group       Pnma Check
PLAT961_ALERT_5_G Dataset Contains no Negative Intensities .......     Please Check
PLAT967_ALERT_5_G Note: Two-Theta Cutoff Value in Embedded .res ..       56.0 Degree
PLAT978_ALERT_2_G Number C-C Bonds with Positive Residual Density.          1 Info
```

0 **ALERT level A** = Most likely a serious problem - resolve or explain
0 **ALERT level B** = A potentially serious problem, consider carefully
2 **ALERT level C** = Check. Ensure it is not caused by an omission or oversight
5 **ALERT level G** = General information/check it is not something unexpected

0 ALERT type 1 CIF construction/syntax error, inconsistent or missing data
3 ALERT type 2 Indicator that the structure model may be wrong or deficient
2 ALERT type 3 Indicator that the structure quality may be low
0 ALERT type 4 Improvement, methodology, query or suggestion
2 ALERT type 5 Informative message, check

Datablock: compound_4a

Bond precision:	C-C = 0.0094 A	Wavelength=0.71073	
Cell:	a=32.2445(9)	b=26.2658(8)	c=15.8729(4)
	alpha=90	beta=103.220(2)	gamma=90
Temperature:	180 K		

	Calculated	Reported
Volume	13086.9(6)	13086.9(6)
Space group	P 21/c	P 21/c
Hall group	-P 2ybc	-P 2ybc
Moiety formula	C29 H25 N4 O3 P Ru [+ solvent]	C29 H25 N4 O3 P Ru, C7 H8
Sum formula	C29 H25 N4 O3 P Ru [+ solvent]	C36 H33 N4 O3 P Ru
Mr	609.57	701.70
Dx,g cm-3	1.237	1.425
Z	16	16
Mu (mm-1)	0.559	0.570
F000	4960.0	5760.0
F000'	4943.09	
h,k,lmax	38,31,18	38,31,18
Nref	23064	23043
Tmin,Tmax	0.892,0.977	0.899,0.981
Tmin'	0.892	

Correction method= # Reported T Limits: Tmin=0.899 Tmax=0.981
AbsCorr = INTEGRATION

Data completeness= 0.999 Theta(max)= 25.000

R(reflections)= 0.0515(13011) wR2(reflections)= 0.1246(23043)

S = 1.018 Npar= 1499

The following ALERTS were generated. Each ALERT has the format
test-name_ALERT_alert-type_alert-level.
Click on the hyperlinks for more details of the test.

Alert level B

```
PLAT241_ALERT_2_B High   'MainMol' Ueq as Compared to Neighbors of        C90 Check
PLAT910_ALERT_3_B Missing # of FCF Reflection(s) Below Theta(Min).         21 Note
```

Alert level C

```
PLAT213_ALERT_2_C Atom C30A            has ADP max/min Ratio .....        3.5 prolat
PLAT213_ALERT_2_C Atom C31A            has ADP max/min Ratio .....        3.5 prolat
PLAT213_ALERT_2_C Atom C32A            has ADP max/min Ratio .....        3.5 prolat
PLAT213_ALERT_2_C Atom C33A            has ADP max/min Ratio .....        3.5 prolat
PLAT213_ALERT_2_C Atom C34A            has ADP max/min Ratio .....        3.5 prolat
PLAT213_ALERT_2_C Atom C35A            has ADP max/min Ratio .....        3.5 prolat
PLAT220_ALERT_2_C NonSolvent   Resd 1  C   Ueq(max)/Ueq(min) Range        3.7 Ratio
PLAT221_ALERT_2_C Solv./Anion  Resd 4  C   Ueq(max)/Ueq(min) Range        4.1 Ratio
PLAT234_ALERT_4_C Large Hirshfeld Difference Ru2      --N5A      .       0.17 Ang.
```

PLAT234_ALERT_4_C Large Hirshfeld Difference C56 --C57 . 0.16 Ang.
PLAT234_ALERT_4_C Large Hirshfeld Difference C68 --C69 . 0.16 Ang.
PLAT234_ALERT_4_C Large Hirshfeld Difference C91 --C92 . 0.17 Ang.
PLAT234_ALERT_4_C Large Hirshfeld Difference C97 --C98 . 0.17 Ang.
PLAT241_ALERT_2_C High 'MainMol' Ueq as Compared to Neighbors of O6 Check
PLAT241_ALERT_2_C High 'MainMol' Ueq as Compared to Neighbors of C56 Check
PLAT241_ALERT_2_C High 'MainMol' Ueq as Compared to Neighbors of C10 Check
PLAT241_ALERT_2_C High 'MainMol' Ueq as Compared to Neighbors of C89 Check
PLAT241_ALERT_2_C High 'MainMol' Ueq as Compared to Neighbors of C114 Check
PLAT242_ALERT_2_C Low 'MainMol' Ueq as Compared to Neighbors of N8 Check
PLAT242_ALERT_2_C Low 'MainMol' Ueq as Compared to Neighbors of C88 Check
PLAT242_ALERT_2_C Low 'MainMol' Ueq as Compared to Neighbors of C91 Check
PLAT242_ALERT_2_C Low 'MainMol' Ueq as Compared to Neighbors of C112 Check
PLAT242_ALERT_2_C Low 'MainMol' Ueq as Compared to Neighbors of C116 Check
PLAT250_ALERT_2_C Large U3/U1 Ratio for Average U(i,j) Tensor .... 2.1 Note
PLAT329_ALERT_4_C Carbon Atom Hybridisation Unclear for .... C36A Check
PLAT329_ALERT_4_C Carbon Atom Hybridisation Unclear for .... C42A Check
PLAT342_ALERT_3_C Low Bond Precision on C-C Bonds .... 0.0094 Ang.
PLAT906_ALERT_3_C Large K Value in the Analysis of Variance ...... 6.039 Check
PLAT906_ALERT_3_C Large K Value in the Analysis of Variance ...... 2.344 Check
PLAT934_ALERT_3_C Number of (Iobs-Icalc)/Sigma(W) > 10 Outliers .. 1 Check

---

Alert level G

FORMU01_ALERT_2_G There is a discrepancy between the atom counts in the
_chemical_formula_sum and the formula from the _atom_site* data.
Atom count from _chemical_formula_sum:C36 H33 N4 O3 P1 Ru1
Atom count from the _atom_site data: C29 H25 N4 O3 P1 Ru1
CELLZ01_ALERT_1_G Difference between formula and atom_site contents detected.
CELLZ01_ALERT_1_G ALERT: Large difference may be due to a
symmetry error - see SYMMG tests
From the CIF: _cell_formula_units_Z 16
From the CIF: _chemical_formula_sum C36 H33 N4 O3 P Ru
TEST: Compare cell contents of formula and atom_site data

atom	Z*formula	cif sites	diff
C	576.00	464.00	112.00
H	528.00	400.00	128.00
N	64.00	64.00	0.00
O	48.00	48.00	0.00
P	16.00	16.00	0.00
Ru	16.00	16.00	0.00

PLAT002_ALERT_2_G Number of Distance or Angle Restraints on AtSite 36 Note
PLAT041_ALERT_1_G Calc. and Reported SumFormula Strings Differ Please Check
PLAT066_ALERT_1_G Predicted and Reported Tmin&Tmax Range Identical ? Check
PLAT168_ALERT_4_G The CIF-Embedded .res File Contains EXYZ Records 3 Report
PLAT171_ALERT_4_G The CIF-Embedded .res File Contains EADP Records 6 Report
PLAT174_ALERT_4_G The CIF-Embedded .res File Contains FLAT Records 4 Report
PLAT175_ALERT_4_G The CIF-Embedded .res File Contains SAME Records 2 Report
PLAT176_ALERT_4_G The CIF-Embedded .res File Contains SADI Records 17 Report
PLAT187_ALERT_4_G The CIF-Embedded .res File Contains RIGU Records 5 Report
PLAT232_ALERT_2_G Hirshfeld Test Diff (M-X) Ru2 --P2A . 5.9 s.u.
PLAT232_ALERT_2_G Hirshfeld Test Diff (M-X) Ru2 --P2C . 5.9 s.u.
PLAT232_ALERT_2_G Hirshfeld Test Diff (M-X) Ru1 --C19 . 5.3 s.u.
PLAT301_ALERT_3_G Main Residue Disorder ....(Resd 1) 39% Note
PLAT302_ALERT_4_G Anion/Solvent/Minor-Residue Disorder (Resd 2) 18% Note
PLAT432_ALERT_2_G Short Inter X...Y Contact O1 ..C38A . 2.93 Ang.

```
                                                    1-x,1-y,1-z  =      3_666 Check
PLAT432_ALERT_2_G Short Inter X...Y Contact  O7       ..C96      .       2.98 Ang.
                                                          x,y,z  =      1_555 Check
PLAT606_ALERT_4_G Solvent Accessible VOID(S) in Structure ........          ! Info
PLAT793_ALERT_4_G Model has Chirality at P2B         (Centro SPGR)          R Verify
PLAT860_ALERT_3_G Number of Least-Squares Restraints .............        262 Note
PLAT869_ALERT_4_G ALERTS Related to the Use of SQUEEZE  Suppressed          ! Info
PLAT908_ALERT_2_G Max. Perc. Data with I > 2*s(I) per Res.Shell .      73.69% Note
PLAT909_ALERT_3_G Percentage of I>2sig(I) Data at Theta(Max) Still        34% Note
PLAT913_ALERT_3_G Missing # of Very Strong Reflections in FCF ....          1 Note
PLAT961_ALERT_5_G Dataset Contains no Negative Intensities .......     Please Check
PLAT967_ALERT_5_G Note: Two-Theta Cutoff Value in Embedded .res ..       50.0 Degree
PLAT978_ALERT_2_G Number C-C Bonds with Positive Residual Density.          0 Info
```

---

0 **ALERT level A** = Most likely a serious problem - resolve or explain
2 **ALERT level B** = A potentially serious problem, consider carefully
30 **ALERT level C** = Check. Ensure it is not caused by an omission or oversight
29 **ALERT level G** = General information/check it is not something unexpected

4 ALERT type 1 CIF construction/syntax error, inconsistent or missing data
29 ALERT type 2 Indicator that the structure model may be wrong or deficient
9 ALERT type 3 Indicator that the structure quality may be low
17 ALERT type 4 Improvement, methodology, query or suggestion
2 ALERT type 5 Informative message, check

Datablock: compound_5a

Bond precision: C-C = 0.0032 A   Wavelength=0.71073

Cell:	a=9.4331(2)	b=14.6220(4)	c=15.1733(4)
	alpha=66.690(2)	beta=75.171(2)	gamma=71.763(2)
Temperature:	180 K		

	Calculated	Reported
Volume	1804.44(8)	1804.44(9)
Space group	P -1	P -1
Hall group	-P 1	-P 1
Moiety formula	2(C33 H34 N5 O2 P Ru), 3(C H2 Cl2)	C33 H34 N5 O2 P Ru, 1.5(C H2 Cl2)
Sum formula	C69 H74 Cl6 N10 O4 P2 Ru2	C34.50 H37 Cl3 N5 O2 P Ru
Mr	1584.16	792.08
Dx,g cm-3	1.458	1.458
Z	1	2
Mu (mm-1)	0.739	0.739
F000	810.0	810.0
F000'	808.79	
h,k,lmax	12,19,20	12,19,20
Nref	8737	8731
Tmin,Tmax	0.899,0.929	0.821,0.908
Tmin'	0.772	

Correction method= # Reported T Limits: Tmin=0.821 Tmax=0.908
AbsCorr = INTEGRATION

Data completeness= 0.999 Theta(max)= 28.000

R(reflections)= 0.0285(8169) wR2(reflections)= 0.0729(8731)

S = 1.069 Npar= 497

The following ALERTS were generated. Each ALERT has the format
test-name_ALERT_alert-type_alert-level.
Click on the hyperlinks for more details of the test.

Alert level B

```
PLAT411_ALERT_2_B Short Inter H...H Contact  H7B      ..H7B      .       1.95 Ang.
                                            1-x,1-y,2-z  =      2_667 Check
```

Alert level C

```
PLAT220_ALERT_2_C NonSolvent   Resd 1  C   Ueq(max)/Ueq(min) Range        4.3 Ratio
PLAT222_ALERT_3_C NonSolvent Resd 1  H   Uiso(max)/Uiso(min) Range        4.7 Ratio
PLAT242_ALERT_2_C Low    'MainMol' Ueq as Compared to Neighbors of        C30 Check
PLAT260_ALERT_2_C Large Average Ueq of Residue Including      Cl1      0.110 Check
PLAT260_ALERT_2_C Large Average Ueq of Residue Including     Cl3A      0.197 Check
PLAT911_ALERT_3_C Missing FCF Refl Between Thmin & STh/L=    0.600          6 Report
PLAT913_ALERT_3_C Missing # of Very Strong Reflections in FCF ....          6 Note
```

Alert level G

```
PLAT002_ALERT_2_G Number of Distance or Angle Restraints on AtSite         15 Note
PLAT003_ALERT_2_G Number of Uiso or Uij Restrained non-H Atoms ...          9 Report
PLAT042_ALERT_1_G Calc. and Reported Moiety Formula Strings Differ     Please Check
PLAT045_ALERT_1_G Calculated and Reported Z Differ by a Factor ...         1. Check
PLAT154_ALERT_1_G The s.u.'s on the Cell Angles are Equal ..(Note)      0.002 Degree
PLAT171_ALERT_4_G The CIF-Embedded .res File Contains EADP Records          2 Report
PLAT176_ALERT_4_G The CIF-Embedded .res File Contains SADI Records          4 Report
PLAT177_ALERT_4_G The CIF-Embedded .res File Contains DELU Records          3 Report
PLAT178_ALERT_4_G The CIF-Embedded .res File Contains SIMU Records          3 Report
PLAT232_ALERT_2_G Hirshfeld Test Diff (M-X)  Ru1      --C29      .        7.6 s.u.
PLAT302_ALERT_4_G Anion/Solvent/Minor-Residue Disorder (Resd  2  )       100% Note
PLAT302_ALERT_4_G Anion/Solvent/Minor-Residue Disorder (Resd  3  )       100% Note
PLAT302_ALERT_4_G Anion/Solvent/Minor-Residue Disorder (Resd  4  )       100% Note
PLAT302_ALERT_4_G Anion/Solvent/Minor-Residue Disorder (Resd  5  )       100% Note
PLAT302_ALERT_4_G Anion/Solvent/Minor-Residue Disorder (Resd  6  )       100% Note
PLAT304_ALERT_4_G Non-Integer Number of Atoms in ..... (Resd  2  )       2.20 Check
PLAT304_ALERT_4_G Non-Integer Number of Atoms in ..... (Resd  3  )       1.17 Check
PLAT304_ALERT_4_G Non-Integer Number of Atoms in ..... (Resd  4  )       1.63 Check
PLAT304_ALERT_4_G Non-Integer Number of Atoms in ..... (Resd  5  )       1.92 Check
PLAT304_ALERT_4_G Non-Integer Number of Atoms in ..... (Resd  6  )       0.58 Check
PLAT789_ALERT_4_G Atoms with Negative _atom_site_disorder_group  #         10 Check
PLAT860_ALERT_3_G Number of Least-Squares Restraints ............          71 Note
PLAT941_ALERT_3_G Average HKL Measurement Multiplicity ..........         4.6 Low
PLAT961_ALERT_5_G Dataset Contains no Negative Intensities .......     Please Check
PLAT967_ALERT_5_G Note: Two-Theta Cutoff Value in Embedded .res ..       56.0 Degree
PLAT978_ALERT_2_G Number C-C Bonds with Positive Residual Density.          6 Info
```

---

0 **ALERT level A** = Most likely a serious problem - resolve or explain
1 **ALERT level B** = A potentially serious problem, consider carefully
7 **ALERT level C** = Check. Ensure it is not caused by an omission or oversight
26 **ALERT level G** = General information/check it is not something unexpected

3 ALERT type 1 CIF construction/syntax error, inconsistent or missing data
9 ALERT type 2 Indicator that the structure model may be wrong or deficient
5 ALERT type 3 Indicator that the structure quality may be low
15 ALERT type 4 Improvement, methodology, query or suggestion
2 ALERT type 5 Informative message, check

Datablock: compound_5b

Bond precision: C-C = 0.0071 A   Wavelength=0.71073

Cell:	a=21.4034(9)	b=15.1338(7)	c=8.9456(3)
	alpha=90	beta=90	gamma=90
Temperature:	150 K		

	Calculated	Reported
Volume	2897.6(2)	2897.6(2)
Space group	P n m a	P n m a
Hall group	-P 2ac 2n	-P 2ac 2n
Moiety formula	C31 H30 N5 O4 P Ru	C31 H30 N5 O4 P Ru
Sum formula	C31 H30 N5 O4 P Ru	C31 H30 N5 O4 P Ru
Mr	668.64	668.64
Dx,g cm-3	1.533	1.533
Z	4	4
Mu (mm-1)	0.642	0.642
F000	1368.0	1368.0
F000'	1363.83	
h,k,lmax	25,17,10	25,17,10
Nref	2656	2656
Tmin,Tmax	0.940,0.975	0.946,0.981
Tmin'	0.920	

Correction method= # Reported T Limits: Tmin=0.946 Tmax=0.981
AbsCorr = INTEGRATION

Data completeness= 1.000 Theta(max)= 25.000

R(reflections)= 0.0406(1974) wR2(reflections)= 0.0905(2656)

S = 1.078 Npar= 209

The following ALERTS were generated. Each ALERT has the format
test-name_ALERT_alert-type_alert-level.
Click on the hyperlinks for more details of the test.

Alert level C

```
PLAT242_ALERT_2_C Low    'MainMol' Ueq as Compared to Neighbors of        C18 Check
PLAT906_ALERT_3_C Large K Value in the Analysis of Variance ......      5.862 Check
```

Alert level G

```
PLAT232_ALERT_2_G Hirshfeld Test Diff (M-X)  Ru1      --C17      .        6.0 s.u.
PLAT300_ALERT_4_G Atom Site Occupancy of H20B      Constrained at         0.5 Check
PLAT300_ALERT_4_G Atom Site Occupancy of H20C      Constrained at         0.5 Check
PLAT367_ALERT_2_G Long?  C(sp?)-C(sp?) Bond  C18      - C20      .       1.52 Ang.
PLAT909_ALERT_3_G Percentage of I>2sig(I) Data at Theta(Max) Still        56% Note
PLAT961_ALERT_5_G Dataset Contains no Negative Intensities .......     Please Check
PLAT967_ALERT_5_G Note: Two-Theta Cutoff Value in Embedded .res ..       50.0 Degree
PLAT978_ALERT_2_G Number C-C Bonds with Positive Residual Density.          1 Info
```

---

0 **ALERT level A** = Most likely a serious problem - resolve or explain
0 **ALERT level B** = A potentially serious problem, consider carefully

```
2 ALERT level C = Check. Ensure it is not caused by an omission or oversight
8 ALERT level G = General information/check it is not something unexpected

0 ALERT type 1 CIF construction/syntax error, inconsistent or missing data
4 ALERT type 2 Indicator that the structure model may be wrong or deficient
2 ALERT type 3 Indicator that the structure quality may be low
2 ALERT type 4 Improvement, methodology, query or suggestion
2 ALERT type 5 Informative message, check
```

Datablock: compound_6a

Bond precision: C-C = 0.0056 A Wavelength=0.71073

Cell:	a=13.2082(5)	b=12.7594(3)	c=17.3007(6)
	alpha=90	beta=97.635(3)	gamma=90
Temperature:	200 K		

	Calculated	Reported
Volume	2889.82(16)	2889.82(16)
Space group	P 21/n	P 21/n
Hall group	-P 2yn	-P 2yn
Moiety formula	C24 H21 Cl N3 O2 P Ru, C H2 Cl2 [+ solvent]	C24 H21 Cl N3 O2 P Ru, 1.5(C H2 Cl2)
Sum formula	C25 H23 Cl3 N3 O2 P Ru [+ solvent]	C25.50 H24 Cl4 N3 O2 P Ru
Mr	635.85	678.32
Dx,g cm-3	1.462	1.559
Z	4	4
Mu (mm-1)	0.901	0.996
F000	1280.0	1364.0
F000’	1277.48	
h,k,lmax	16,16,22	16,16,22
Nref	6316	6315
Tmin,Tmax	0.787,0.861	0.800,0.924
Tmin’	0.706	

Correction method= # Reported T Limits: Tmin=0.800 Tmax=0.924
AbsCorr = INTEGRATION

Data completeness= 1.000 Theta(max)= 27.000

R(reflections)= 0.0384(5475) wR2(reflections)= 0.0865(6315)

S = 1.081 Npar= 316

The following ALERTS were generated. Each ALERT has the format
test-name_ALERT_alert-type_alert-level.
Click on the hyperlinks for more details of the test.

Alert level C

```
PLAT244_ALERT_4_C Low    'Solvent' Ueq as Compared to Neighbors of        C25 Check
PLAT906_ALERT_3_C Large K Value in the Analysis of Variance ......      3.976 Check
```

Alert level G

```
FORMU01_ALERT_2_G  There is a discrepancy between the atom counts in the
            _chemical_formula_sum and the formula from the _atom_site* data.
            Atom count from _chemical_formula_sum:C25.5 H24 Cl4 N3 O2 P1 Ru1
            Atom count from the _atom_site data:  C25 H23 Cl3 N3 O2 P1 Ru1
CELLZ01_ALERT_1_G Difference between formula and atom_site contents detected.
CELLZ01_ALERT_1_G ALERT: Large difference may be due to a
            symmetry error - see SYMMG tests
           From the CIF: _cell_formula_units_Z    4
           From the CIF: _chemical_formula_sum  C25.50 H24 Cl4 N3 O2 P Ru
           TEST: Compare cell contents of formula and atom_site data

           atom    Z*formula  cif sites diff
           C        102.00    100.00    2.00
           H         96.00     92.00    4.00
           Cl        16.00     12.00    4.00
           N         12.00     12.00    0.00
           O          8.00      8.00    0.00
           P          4.00      4.00    0.00
           Ru         4.00      4.00    0.00
PLAT041_ALERT_1_G Calc. and Reported SumFormula    Strings  Differ     Please Check
PLAT042_ALERT_1_G Calc. and Reported Moiety Formula Strings Differ     Please Check
PLAT051_ALERT_1_G Mu(calc) and Mu(CIF) Ratio Differs from 1.0 by .       9.53 %
PLAT230_ALERT_2_G Hirshfeld Test Diff for    O2       --C24      .        5.5 s.u.
PLAT232_ALERT_2_G Hirshfeld Test Diff (M-X)  Ru1      --C24      .        8.2 s.u.
PLAT605_ALERT_4_G Largest Solvent Accessible VOID in the Structure        178 A**3
PLAT869_ALERT_4_G ALERTS Related to the Use of SQUEEZE  Suppressed          ! Info
PLAT961_ALERT_5_G Dataset Contains no Negative Intensities .......     Please Check
PLAT965_ALERT_2_G The SHELXL WEIGHT Optimisation has not Converged     Please Check
PLAT967_ALERT_5_G Note: Two-Theta Cutoff Value in Embedded .res ..       54.0 Degree
PLAT978_ALERT_2_G Number C-C Bonds with Positive Residual Density.          1 Info
```

0 **ALERT level A** = Most likely a serious problem - resolve or explain
0 **ALERT level B** = A potentially serious problem, consider carefully
2 **ALERT level C** = Check. Ensure it is not caused by an omission or oversight
14 **ALERT level G** = General information/check it is not something unexpected

5 ALERT type 1 CIF construction/syntax error, inconsistent or missing data
5 ALERT type 2 Indicator that the structure model may be wrong or deficient
1 ALERT type 3 Indicator that the structure quality may be low
3 ALERT type 4 Improvement, methodology, query or suggestion
2 ALERT type 5 Informative message, check

checkCIF publication errors

Alert level A

```
PUBL004_ALERT_1_A  The contact author's name and address are missing,
           _publ_contact_author_name and _publ_contact_author_address.
PUBL005_ALERT_1_A  _publ_contact_author_email, _publ_contact_author_fax and
           _publ_contact_author_phone are all missing.
           At least one of these should be present.
PUBL006_ALERT_1_A  _publ_requested_journal is missing
           e.g. 'Acta Crystallographica Section C'
PUBL008_ALERT_1_A  _publ_section_title is missing. Title of paper.
PUBL009_ALERT_1_A  _publ_author_name is missing. List of author(s) name(s).
PUBL010_ALERT_1_A  _publ_author_address is missing. Author(s) address(es).
PUBL012_ALERT_1_A  _publ_section_abstract is missing.
           Abstract of paper in English.
```

Alert level G

```
PUBL017_ALERT_1_G The _publ_section_references section is missing or
           empty.
```

---

7 **ALERT level A** = Data missing that is essential or data in wrong format
1 **ALERT level G** = General alerts. Data that may be required is missing

---

Publication of your CIF

You should attempt to resolve as many as possible of the alerts in all categories. Often the minor alerts point to easily fixed oversights, errors and omissions in your CIF or refinement strategy, so attention to these fine details can be worthwhile. In order to resolve some of the more serious problems it may be necessary to carry out additional measurements or structure refinements. However, the nature of your study may justify the reported deviations from journal submission requirements and the more serious of these should be commented upon in the discussion or experimental section of a paper or in the "special_details" fields of the CIF. *checkCIF* was carefully designed to identify outliers and unusual parameters, but every test has its limitations and alerts that are not important in a particular case may appear. Conversely, the absence of alerts does not guarantee there are no aspects of the results needing attention. It is up to the individual to critically assess their own results and, if necessary, seek expert advice.

If level A alerts remain, which you believe to be justified deviations, and you intend to submit this CIF for publication in a journal, you should additionally insert an explanation in your CIF using the Validation Reply Form (VRF) below. This will allow your explanation to be considered as part of the review process.

Validation response form

Please find below a validation response form (VRF) that can be filled in and pasted into your CIF.

```
# start Validation Reply Form
_vrf_PUBL004_GLOBAL
;
PROBLEM: The contact author's name and address are missing,
RESPONSE: ...
;
_vrf_PUBL005_GLOBAL
;
PROBLEM: _publ_contact_author_email, _publ_contact_author_fax and
RESPONSE: ...
;
_vrf_PUBL006_GLOBAL
;
PROBLEM: _publ_requested_journal is missing
RESPONSE: ...
;
_vrf_PUBL008_GLOBAL
;
PROBLEM: _publ_section_title is missing. Title of paper.
RESPONSE: ...
;
_vrf_PUBL009_GLOBAL
;
PROBLEM: _publ_author_name is missing. List of author(s) name(s).
RESPONSE: ...
;
_vrf_PUBL010_GLOBAL
;
PROBLEM: _publ_author_address is missing. Author(s) address(es).
RESPONSE: ...
;
_vrf_PUBL012_GLOBAL
;
PROBLEM: _publ_section_abstract is missing.
RESPONSE: ...
;
# end Validation Reply Form
```

If you wish to submit your CIF for publication in Acta Crystallographica Section C or E, you should upload your CIF via the web. If you wish to submit your CIF for publication in IUCrData you should upload your CIF via the web. If your CIF is to form part of a submission to another IUCr journal, you will be asked, either during electronic submission or by the Co-editor handling your paper, to upload your CIF via our web site.

PLATON version of 19/02/2022; check.def file version of 19/01/2022

Datablock compound_3a - ellipsoid plot

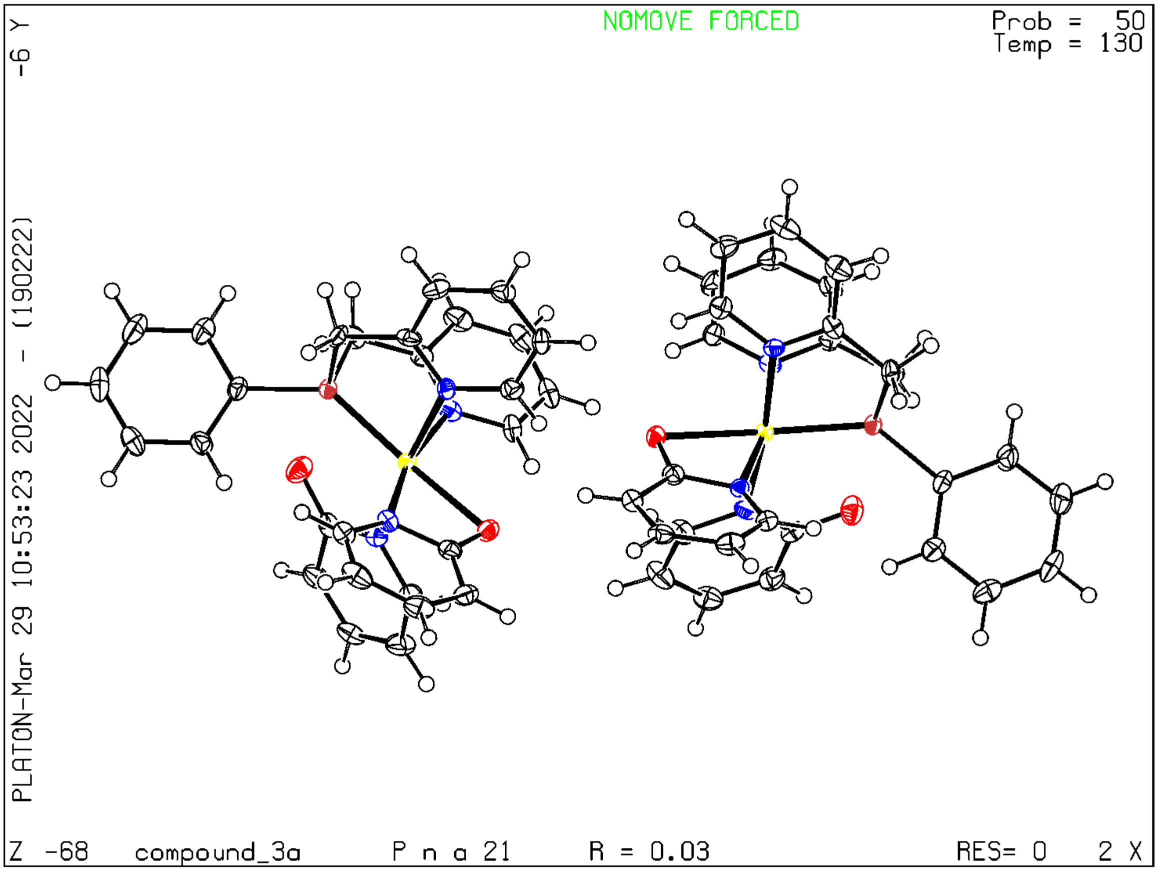

Datablock compound_4a - ellipsoid plot

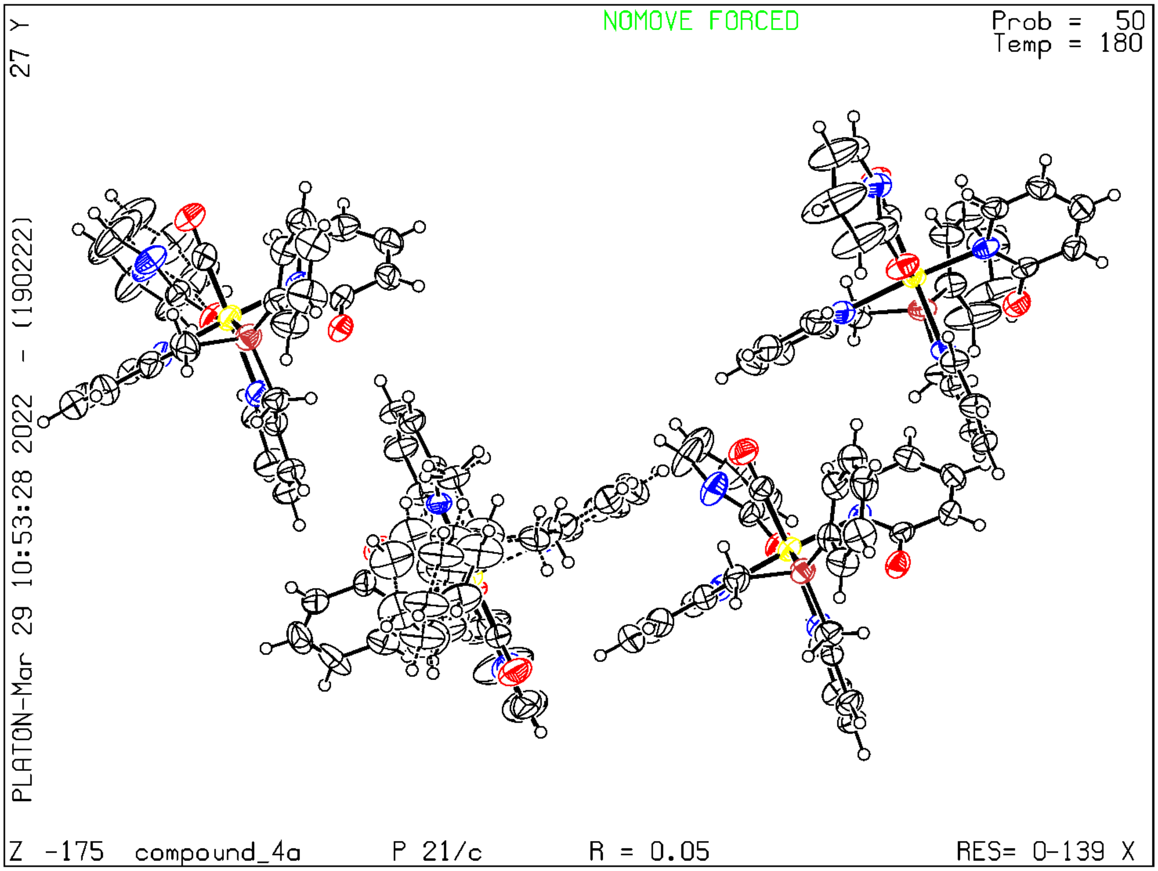

Datablock compound_5a - ellipsoid plot

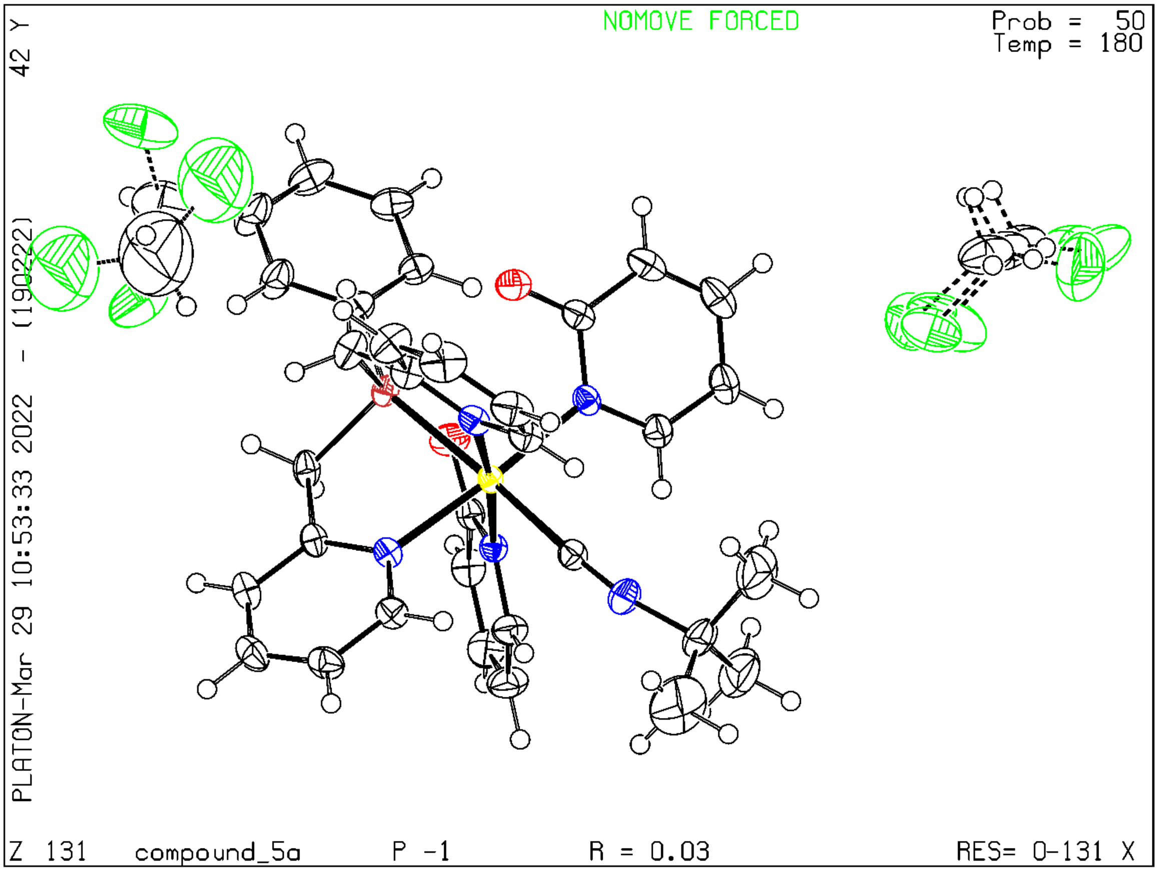

Datablock compound_5b - ellipsoid plot

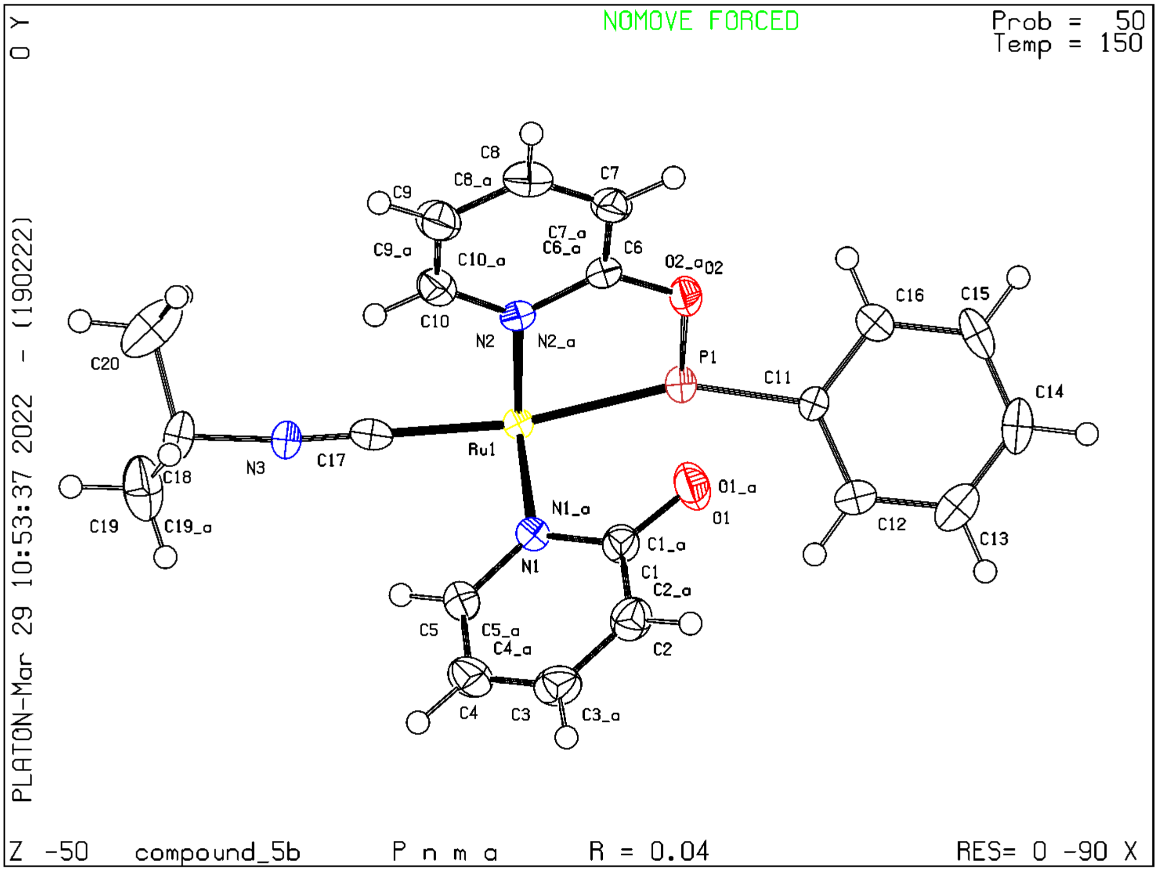

Datablock compound_6a - ellipsoid plot

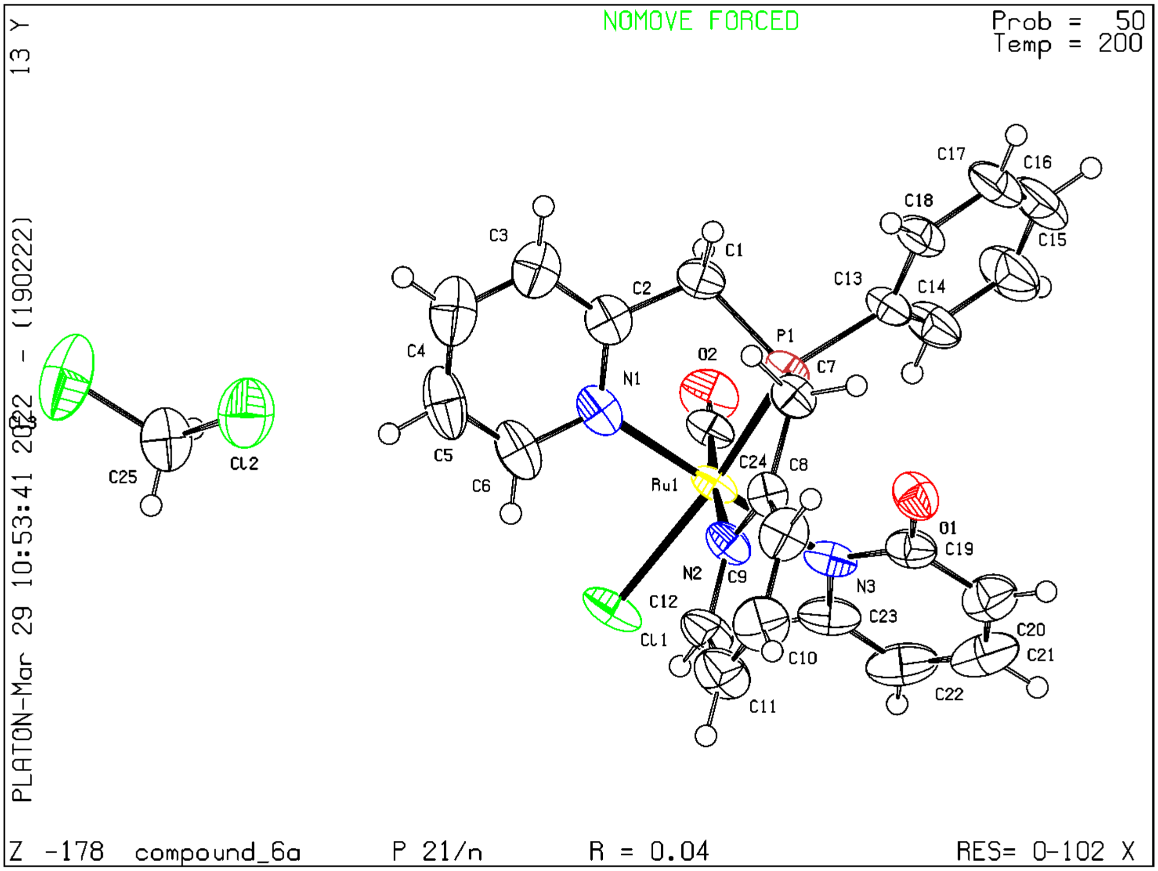

Supplement: Supplementary file 1 [file molecules-27-02778-s001.zip › checkcif_EhrlichGerickeBrendlerWagler_all-in-one.pdf]
