# Supplementary material for: P–Ru-Complexes with a Chelate-Bridge-Switch: A Comparison of 2-Picolyl and 2-Pyridyloxy Moieties as Bridging Ligands
Source: Molecules. 2022 Apr 27;27(9):2778. doi: 10.3390/molecules27092778 (PMC9103559; doi:10.3390/molecules27092778)
Supplement: Supplementary file 1 [file molecules-27-02778-s001.zip › SI_EhrlichGerickeBrendlerWagler_Molecules.pdf]

Supporting information for:

P–Ru-Complexes with a Chelate-Bridge-Switch: A Comparison of 2-Picolyl and 2-Pyridyloxy Moieties as Bridging Ligands

Lisa Ehrlich,[1] Robert Gericke,[1,2] Erica Brendler,[3] and Jörg Wagler[1]

[1] Technische Universität Bergakademie Freiberg, Institut für Anorganische Chemie, Leipziger Straße 29, 09596 Freiberg, Germany

[2] Helmholtz-Zentrum Dresden-Rossendorf e.V., Institute of Resource Ecology, Bautzner Landstraße 400, 01328 Dresden, Germany

[3] Technische Universität Bergakademie Freiberg, Institut für Analytische Chemie, Leipziger Straße 29, 09596 Freiberg, Germany

Contents

NMR spectra (^{1}H, ^{13}C, ^{31}P) of compounds **3a**, **4a**, **5a**, **5b**, **6a**.

Sets of graphical representation, total energies in a.u. (PBE0: final single point energy and final Gibbs free energy; B2T-PLYP: final single point energy) and atomic coordinates of optimized molecular structures of $\mathbf{4a^1}$, $\mathbf{4a^2}$, $\mathbf{4a^3}$, $\mathbf{4a^4}$, $\mathbf{4a^5}$, $\mathbf{4a^6}$, $\mathbf{4a^7}$, $\mathbf{5a^1}$, $\mathbf{5a^3}$, $\mathbf{5b^1}$, $\mathbf{5b^3}$, $\mathbf{4b^1}$, $\mathbf{4b^3}$.

Graphical representations of NLMOs and NBOs

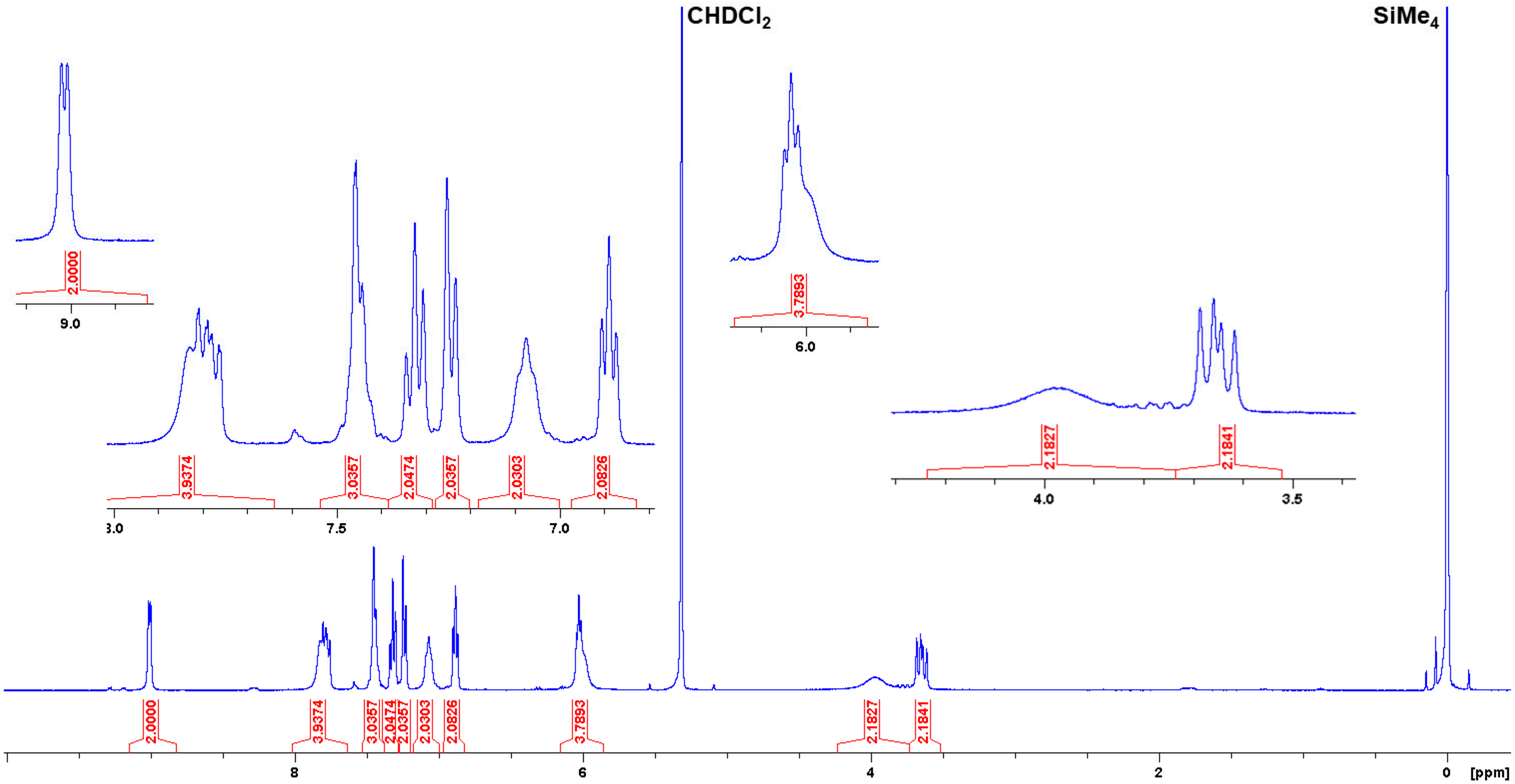

Figure S1. ^{1}H NMR spectrum of **3a** in CD_2Cl_2 (full spectrum and magnified insets of the (groups of) signals).

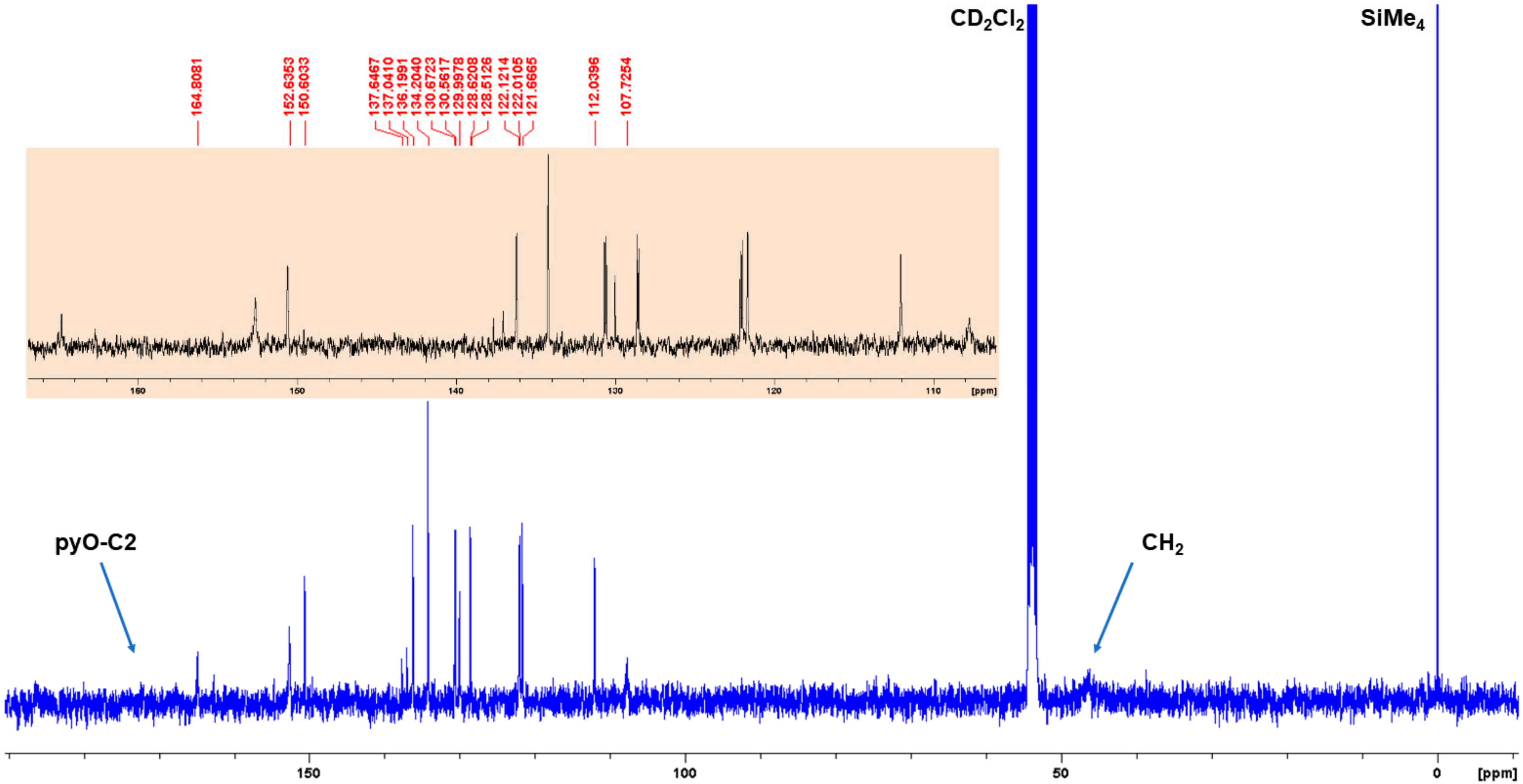

Figure S2. $^{13}C\{^{1}H\}$ NMR spectrum of **3a** in CD_2Cl_2 (full spectrum and magnified inset of the aryl part of signals). The location of the CH_2 and pyO-C2 signals was further confirmed by correlations in the $^{1}H/^{13}C$-HSQC and $^{1}H/^{13}C$-HMBC spectra, respectively.

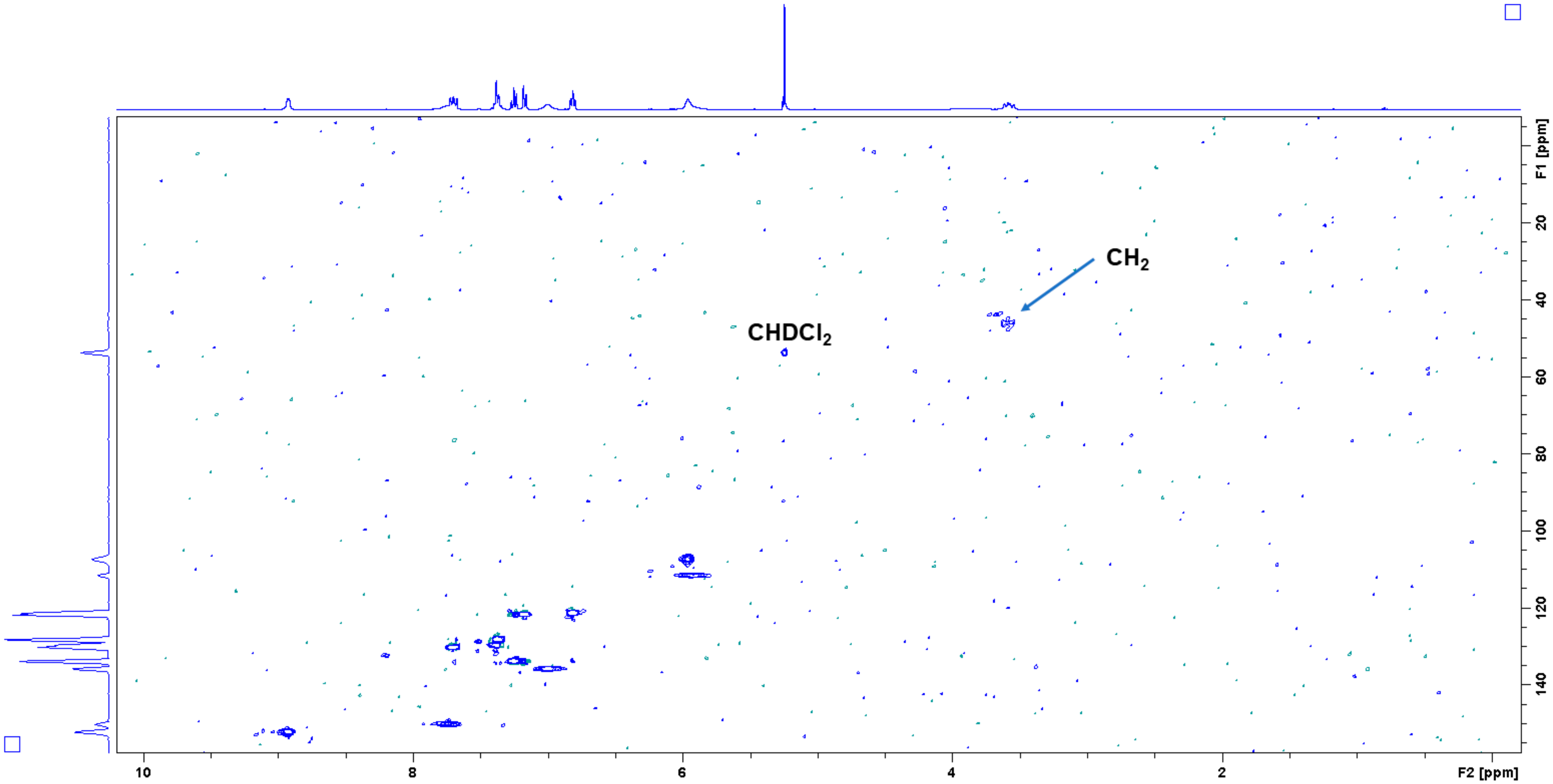

Figure S3. ^{1}H/^{13}C-HSQC NMR spectrum of **3a** in CD_2Cl_2.

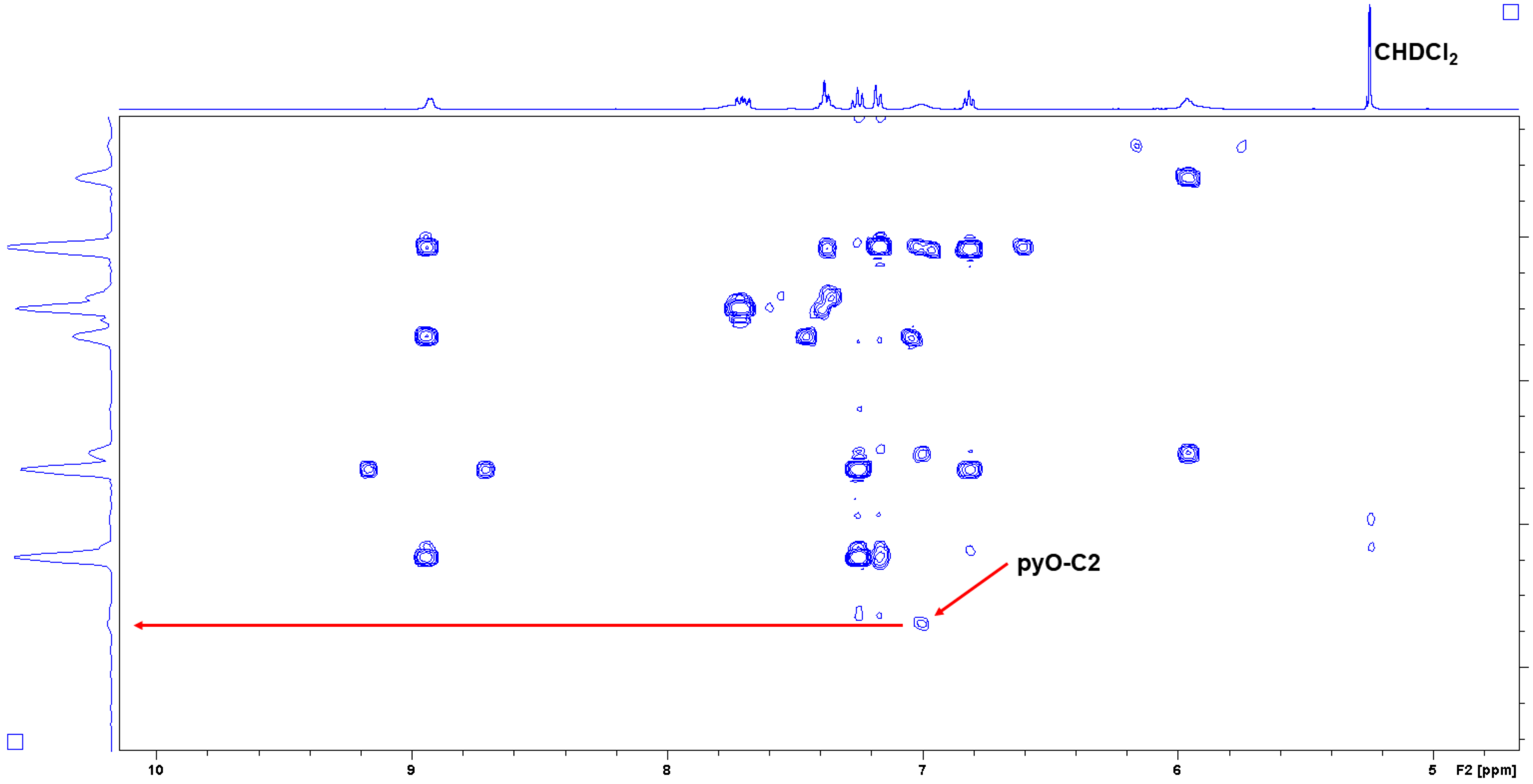

Figure S4. $^{1}H/^{13}C$-HMBC NMR spectrum of **3a** in CD_2Cl_2. (The correlation between ^{13}C(pyO-C2) and ^{1}H(pyO-H4), highlighted with a red arrow, confirmed the ^{13}C NMR shift of the pyO-C2 signal.)

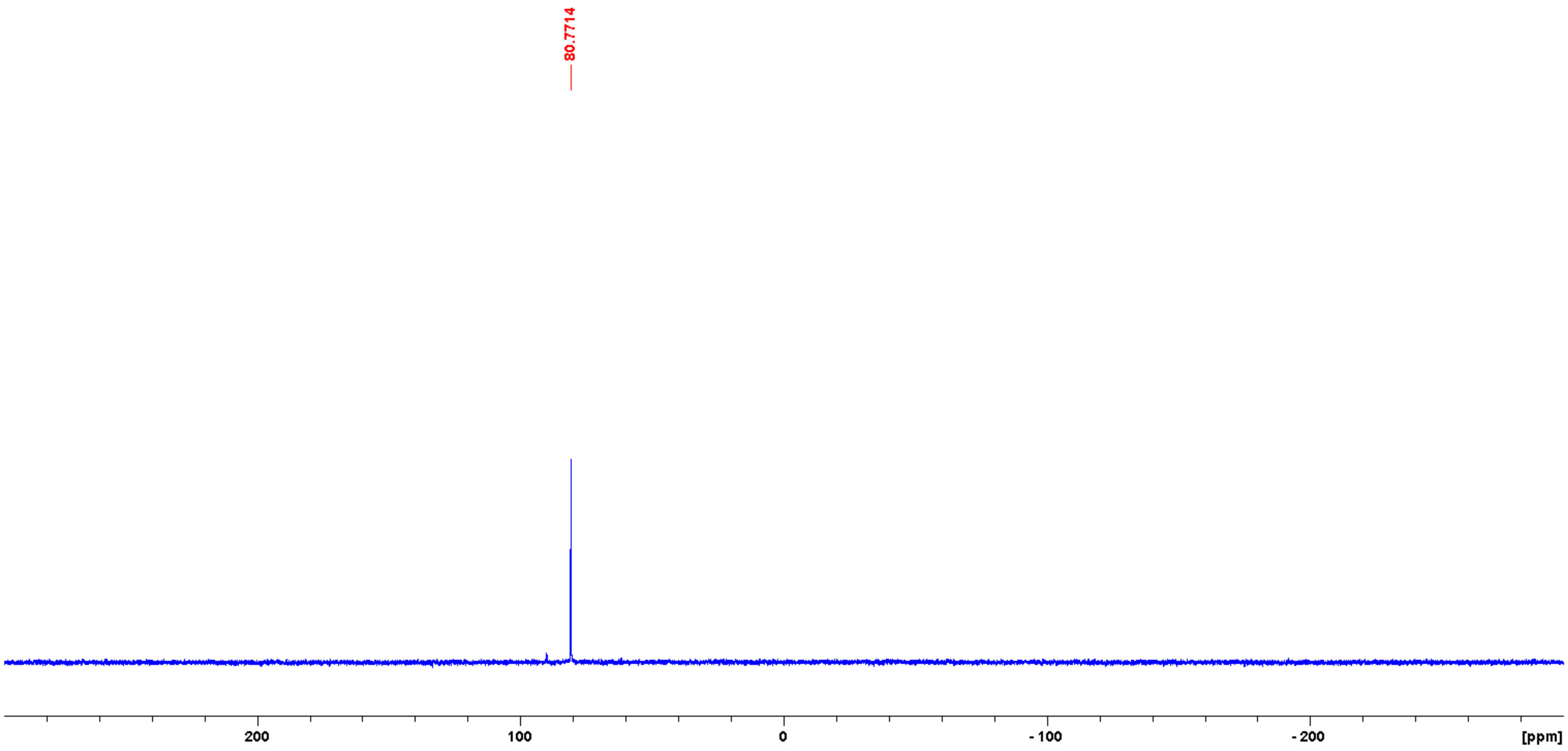

Figure S5. $^{31}P\{^{1}H\}$ NMR spectrum of **3a** in CD_2Cl_2.

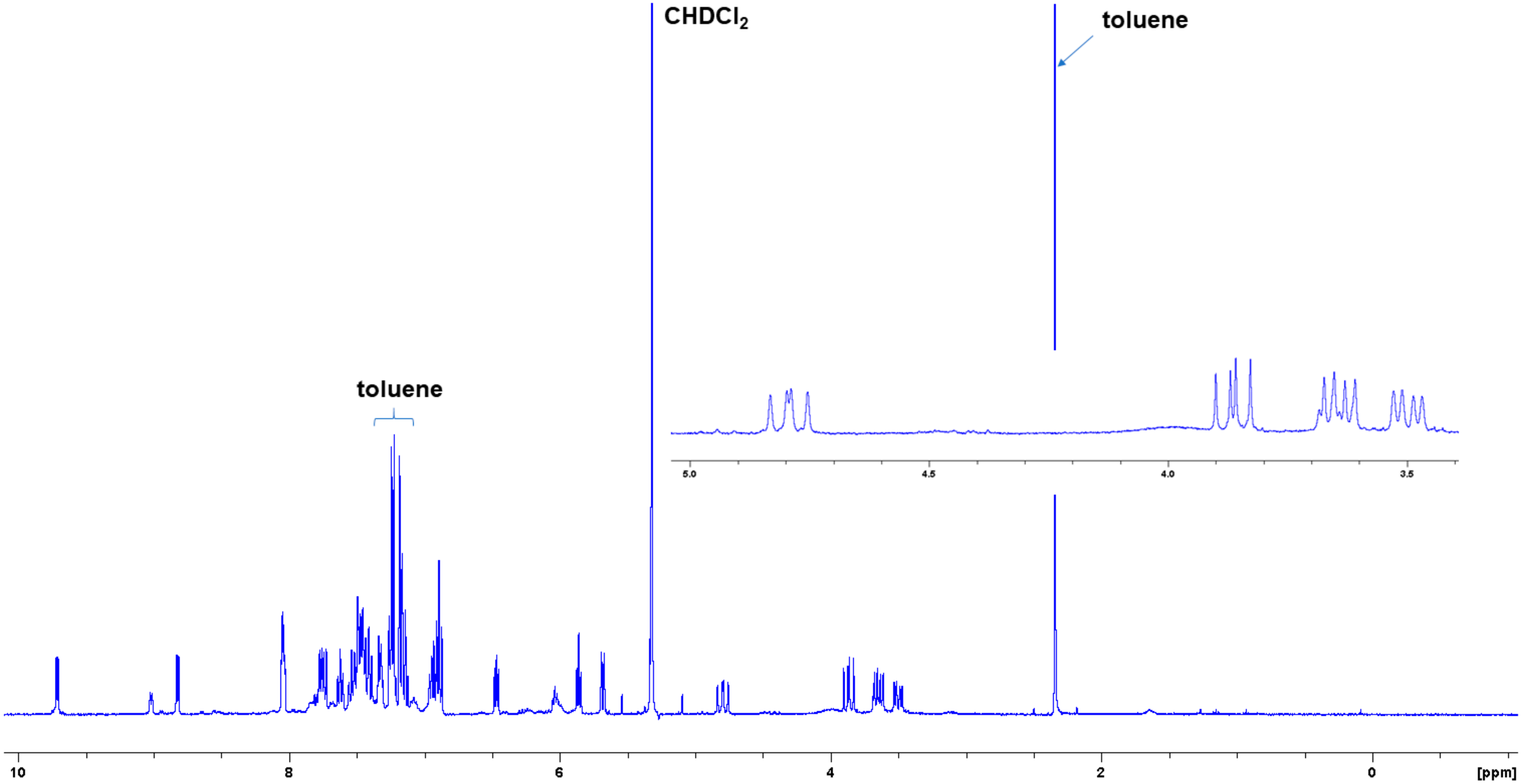

Figure S6. ^{1}H NMR spectrum of (**4a** · toluene) in CD_2Cl_2 (full spectrum and magnified inset of the groups´ signals).

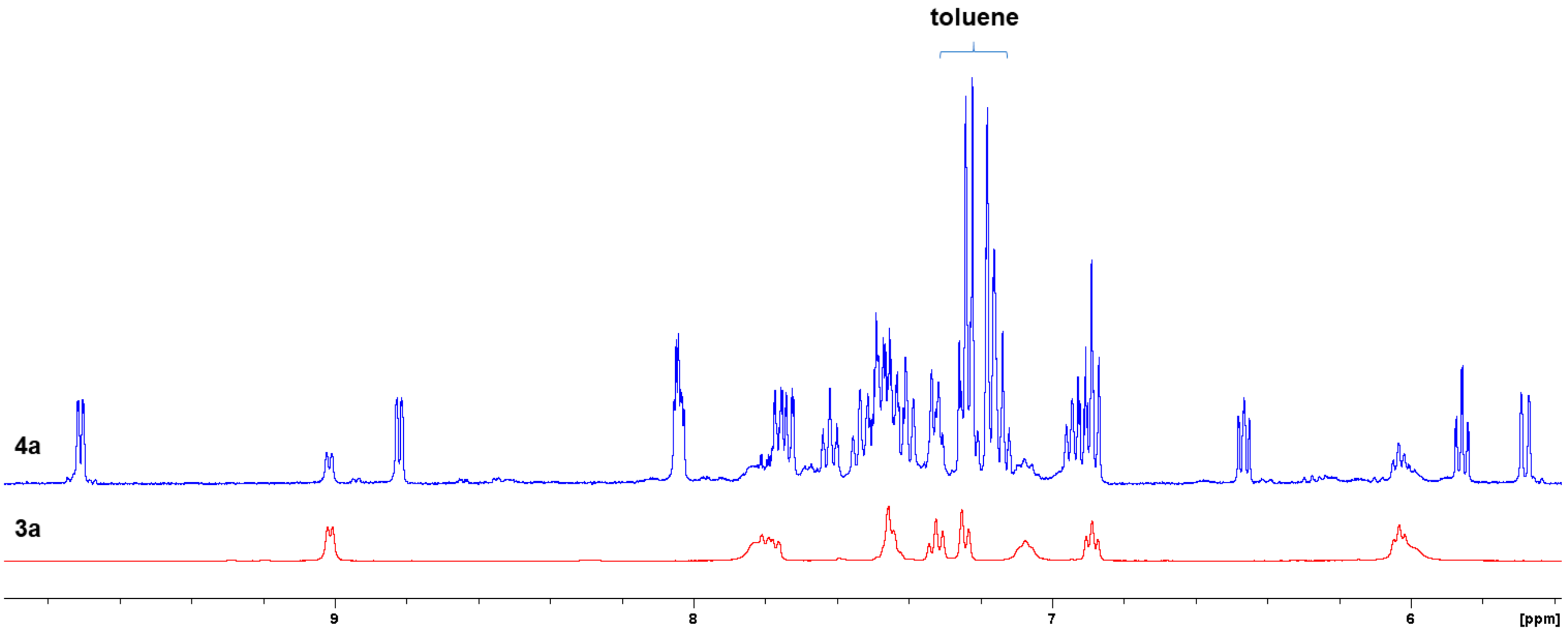

Figure S7. Aryl part of the ^{1}H NMR spectrum of (**4a** · toluene) in CD_2Cl_2 (blue) and overlay of the spectrum of **3a** (red), which is contained in the product as a contamination.

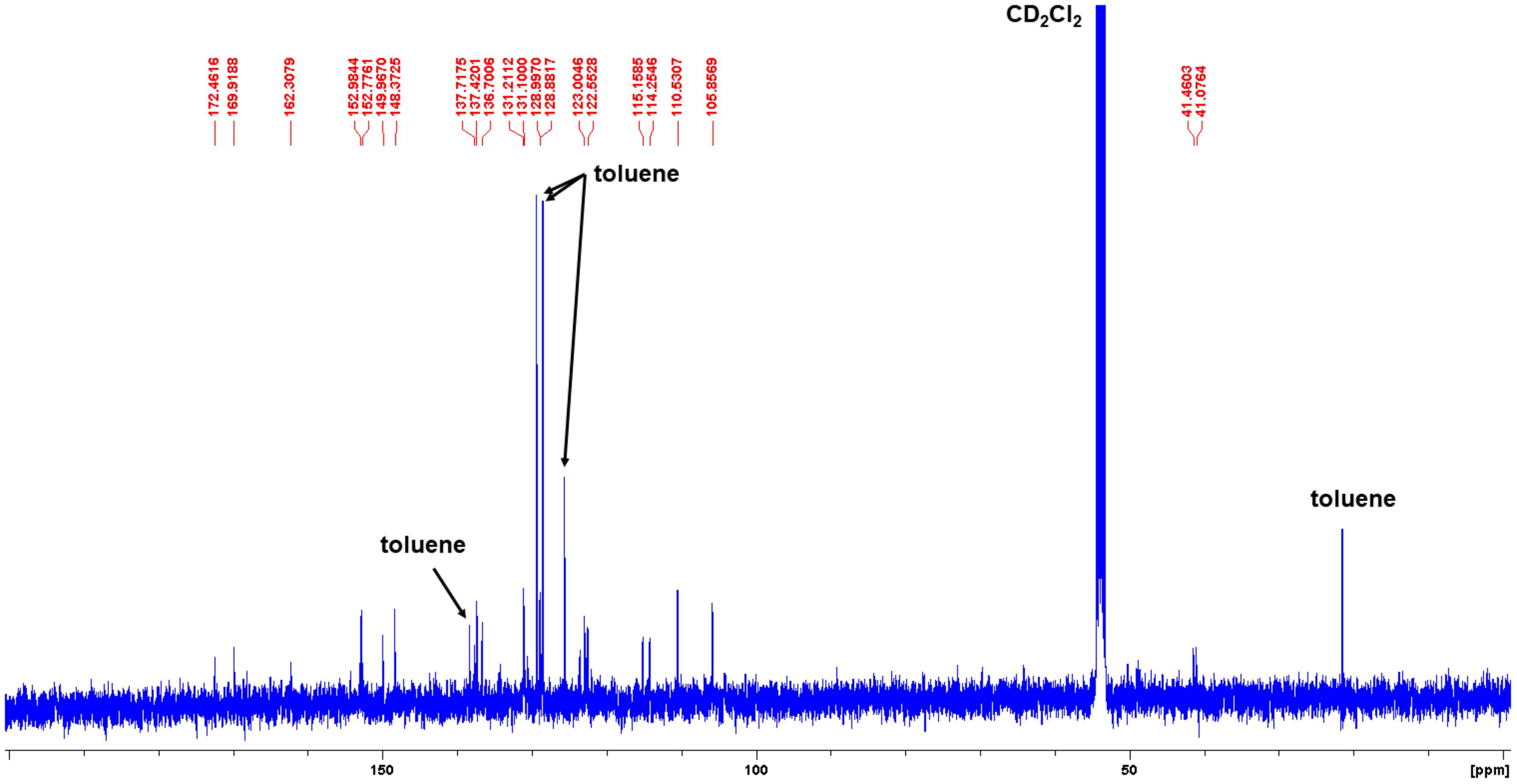

Figure S8. $^{13}C\{^1H\}$ NMR spectrum of (**4a** · toluene) in CD_2Cl_2.

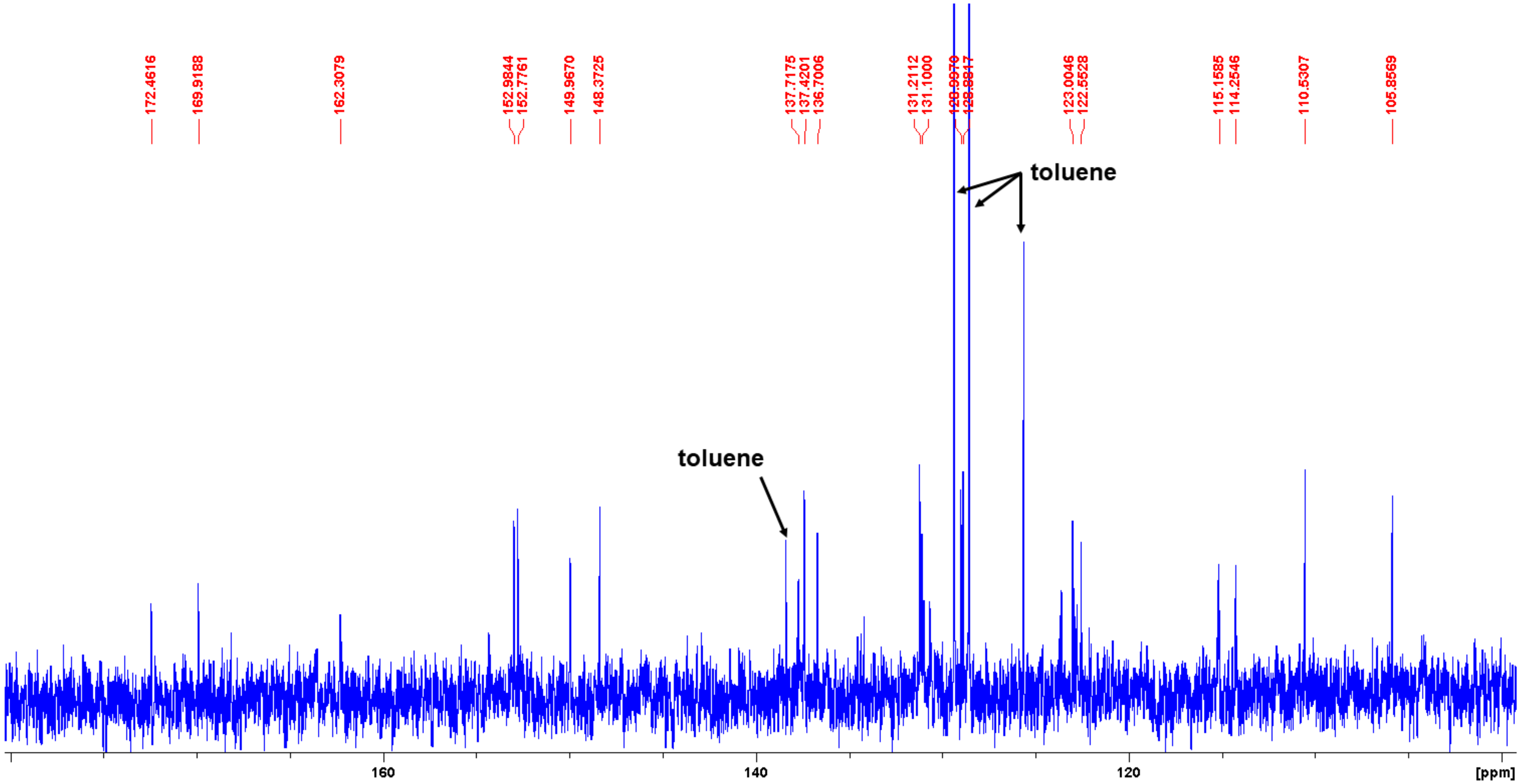

Figure S9. Aryl section of the $^{13}C\{^{1}H\}$ NMR spectrum of (**4a** · toluene) in CD_2Cl_2.

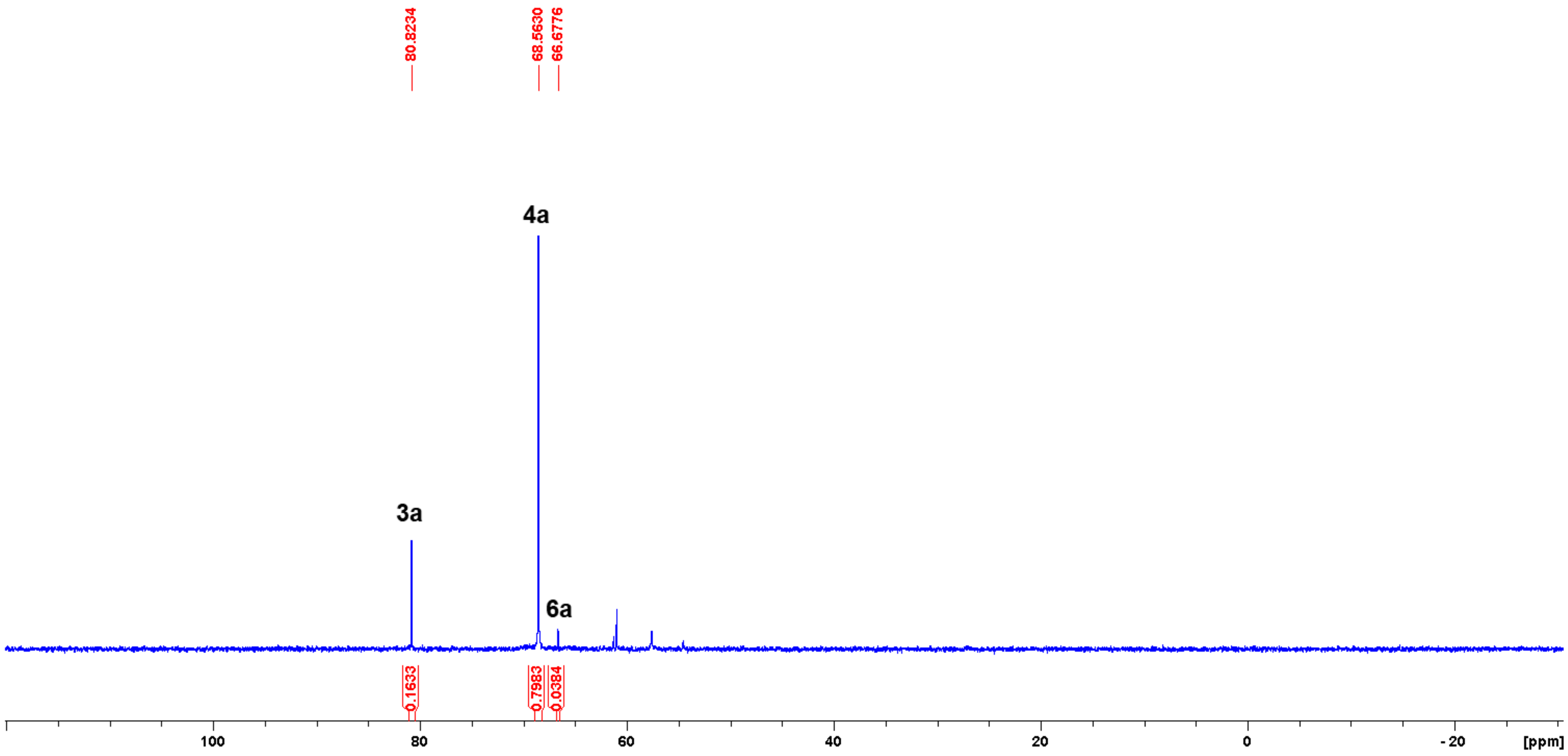

Figure S10. $^{31}P\{^{1}H\}$ NMR spectrum of (**4a** · toluene) in CD_2Cl_2. In addition to the contamination with starting material **3a**, the spectrum contains the signal of decomposition product **6a** (and signals of other still unidentified decomposition products).

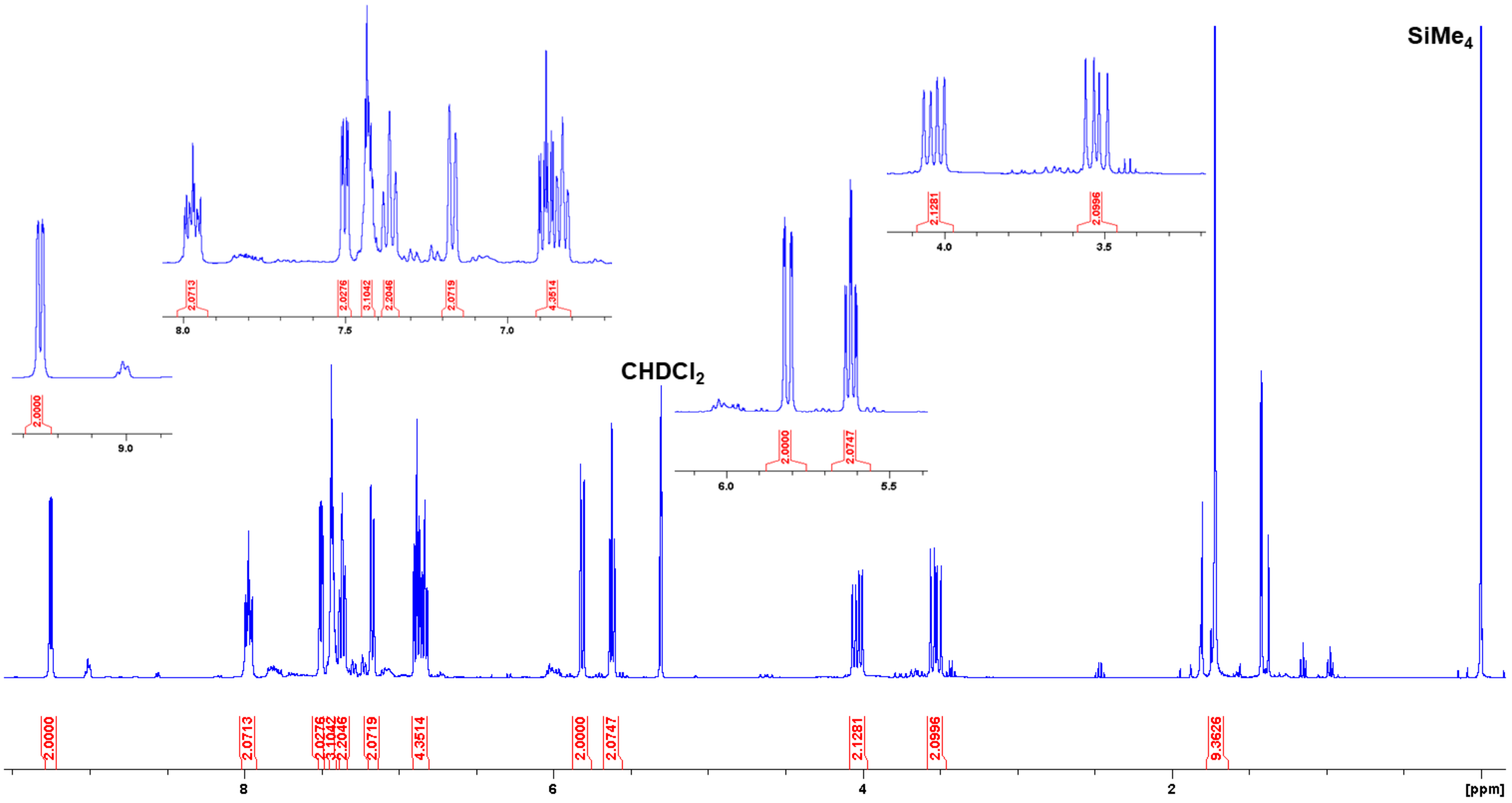

Figure S11. ^{1}H NMR spectrum of **5a** in CD_2Cl_2, sample prepared *in situ* from **3a** and CN*t*Bu (full spectrum and magnified insets of the aryl and CH_2 proton signals).

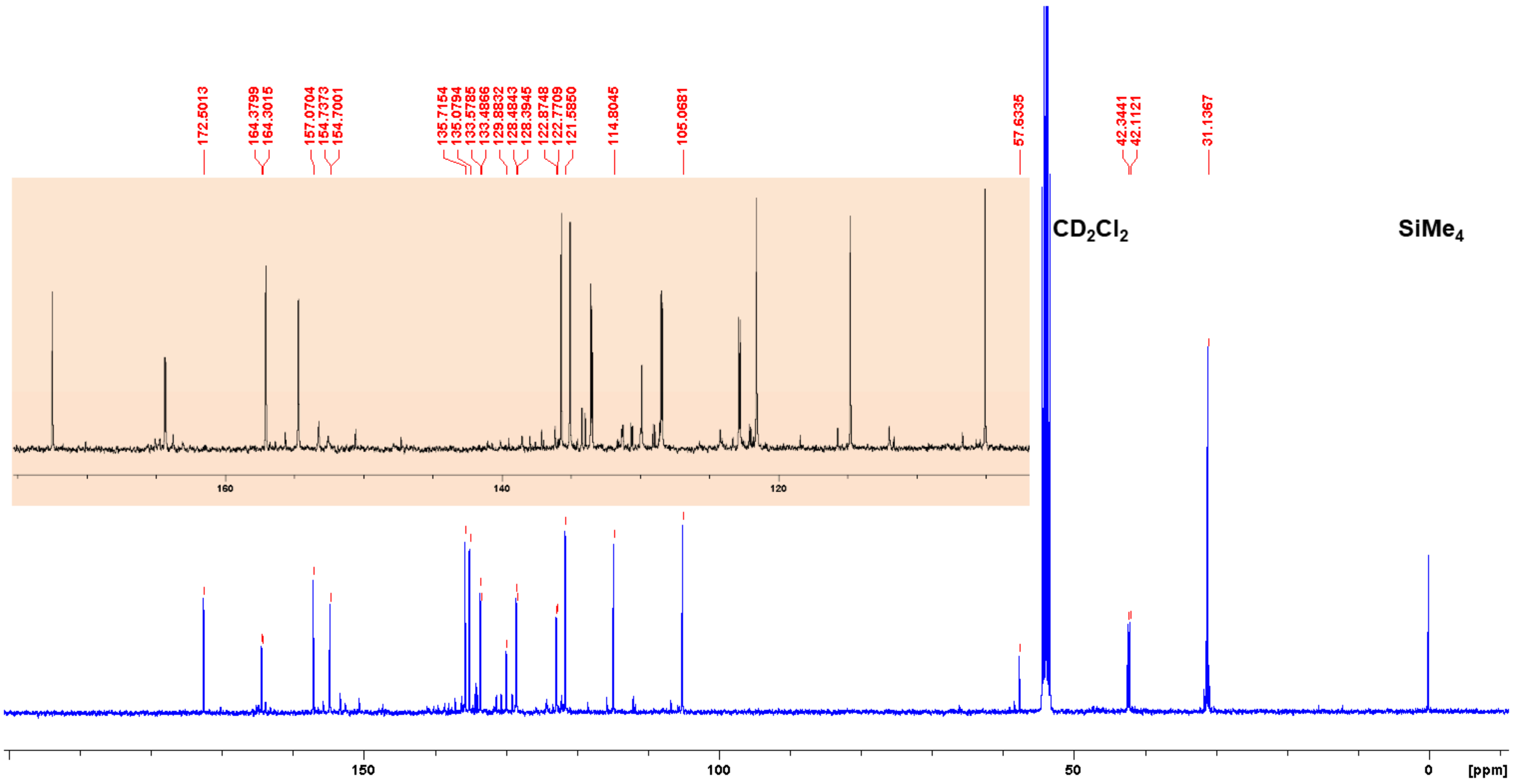

Figure S12. $^{13}C\{^1H\}$ NMR spectrum of **5a** in CD_2Cl_2, sample prepared *in situ* from **3a** and CN*t*Bu (full spectrum and magnified inset of the aryl part of signals). The location of the *C*NCMe3 signal and of the Ph-ipso-*C* signal has not been identified.

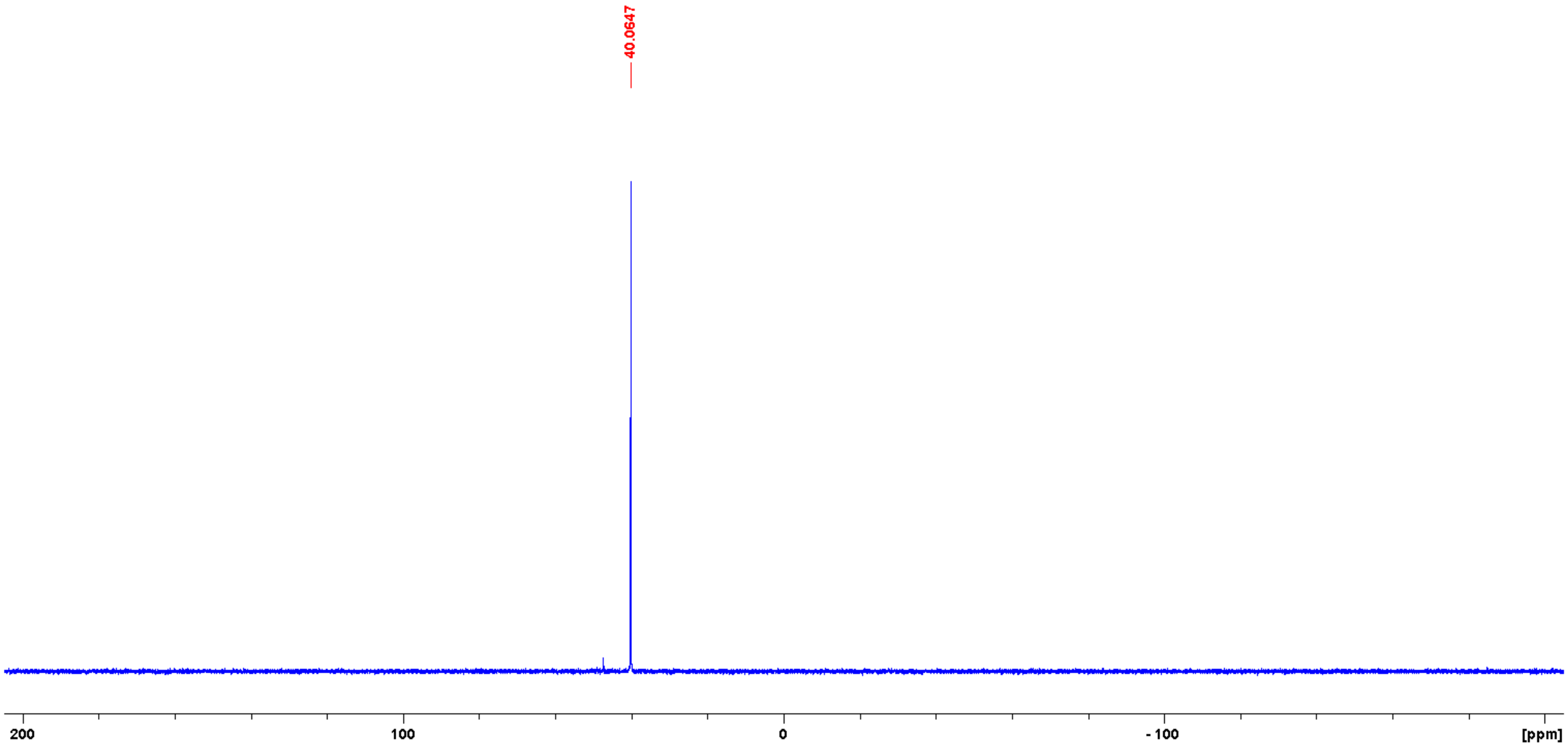

Figure S13. $^{31}P\{^{1}H\}$ NMR spectrum of **5a** in CD_2Cl_2, sample prepared *in situ* from **3a** and CN*t*Bu.

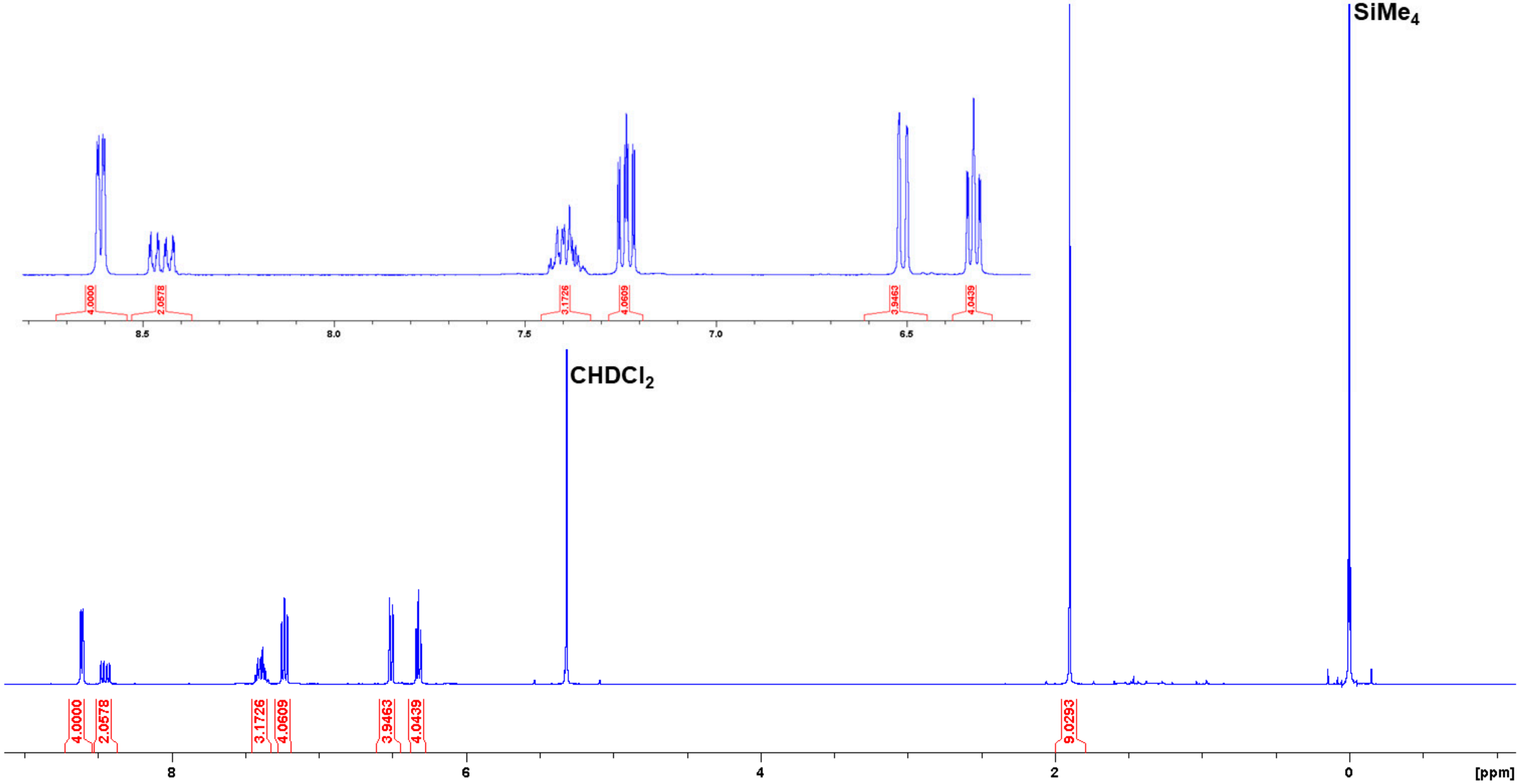

Figure S14. ^{1}H NMR spectrum of **5b** in CD_2Cl_2 (full spectrum and magnified inset of the aryl proton signals).

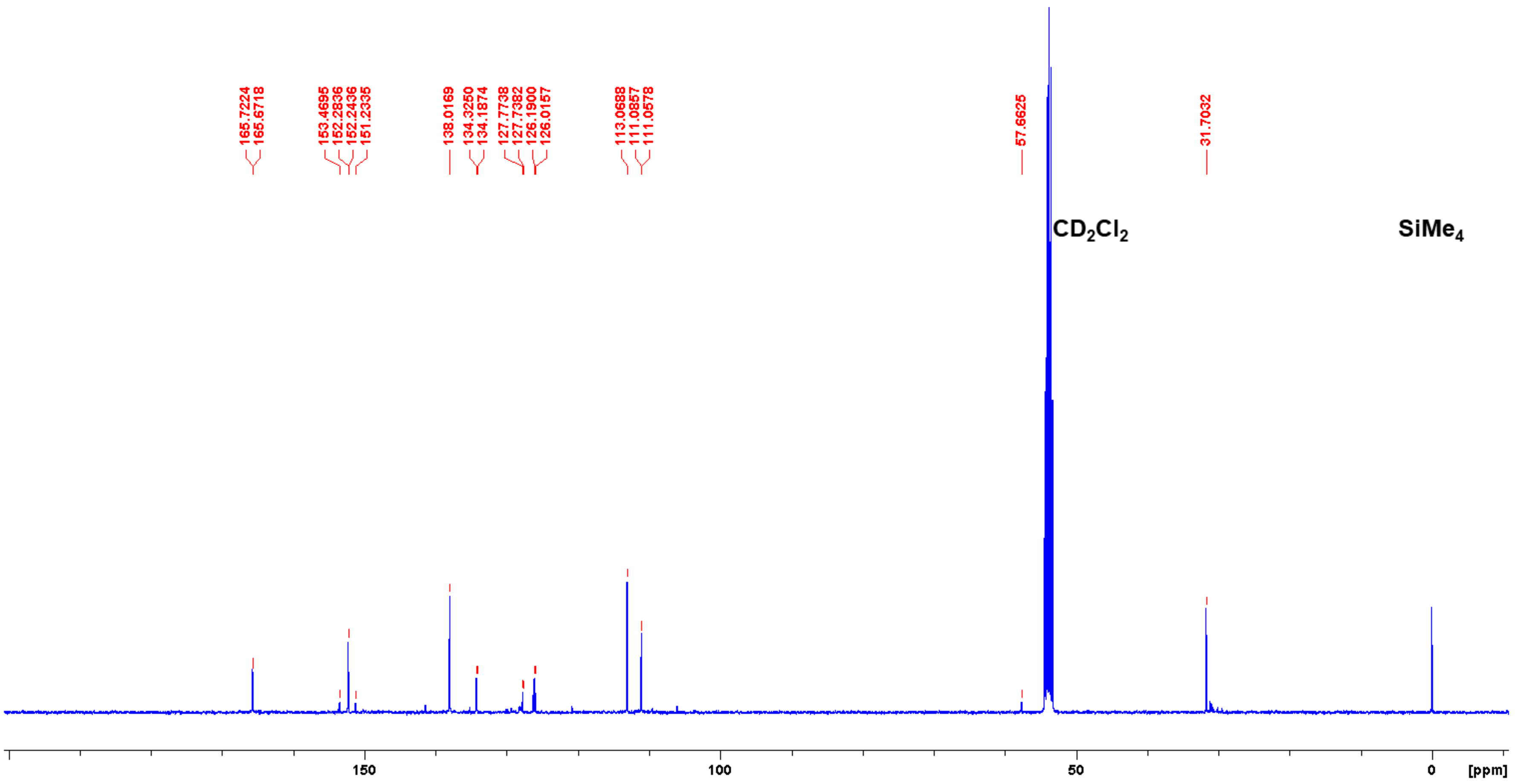

Figure S15. $^{13}C\{^{1}H\}$ NMR spectrum of **5b** in CD_2Cl_2. The location of the *C*NCMe$_3$ signal has not been identified.

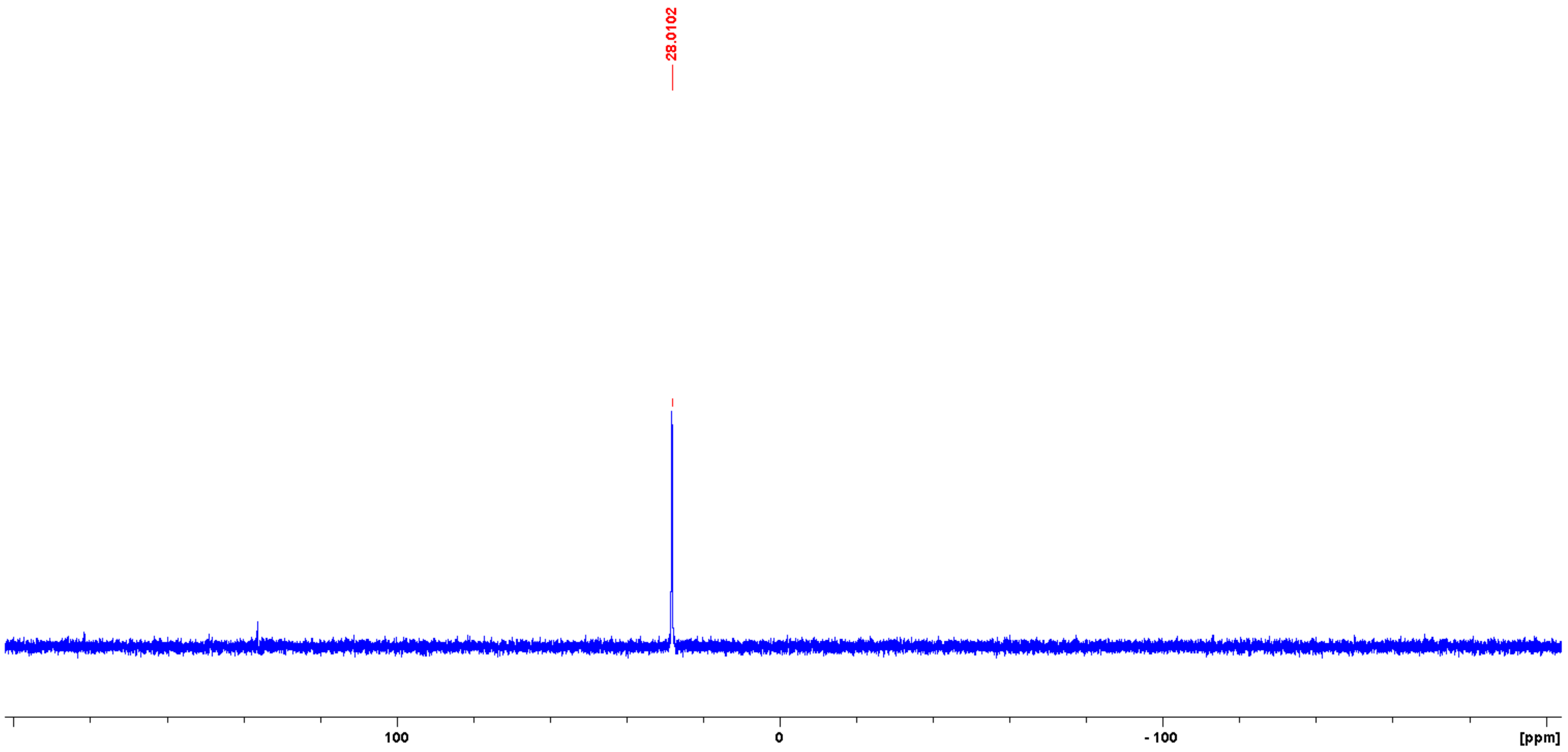

Figure S16. $^{31}P\{^{1}H\}$ NMR spectrum of **5b** in CD_2Cl_2.

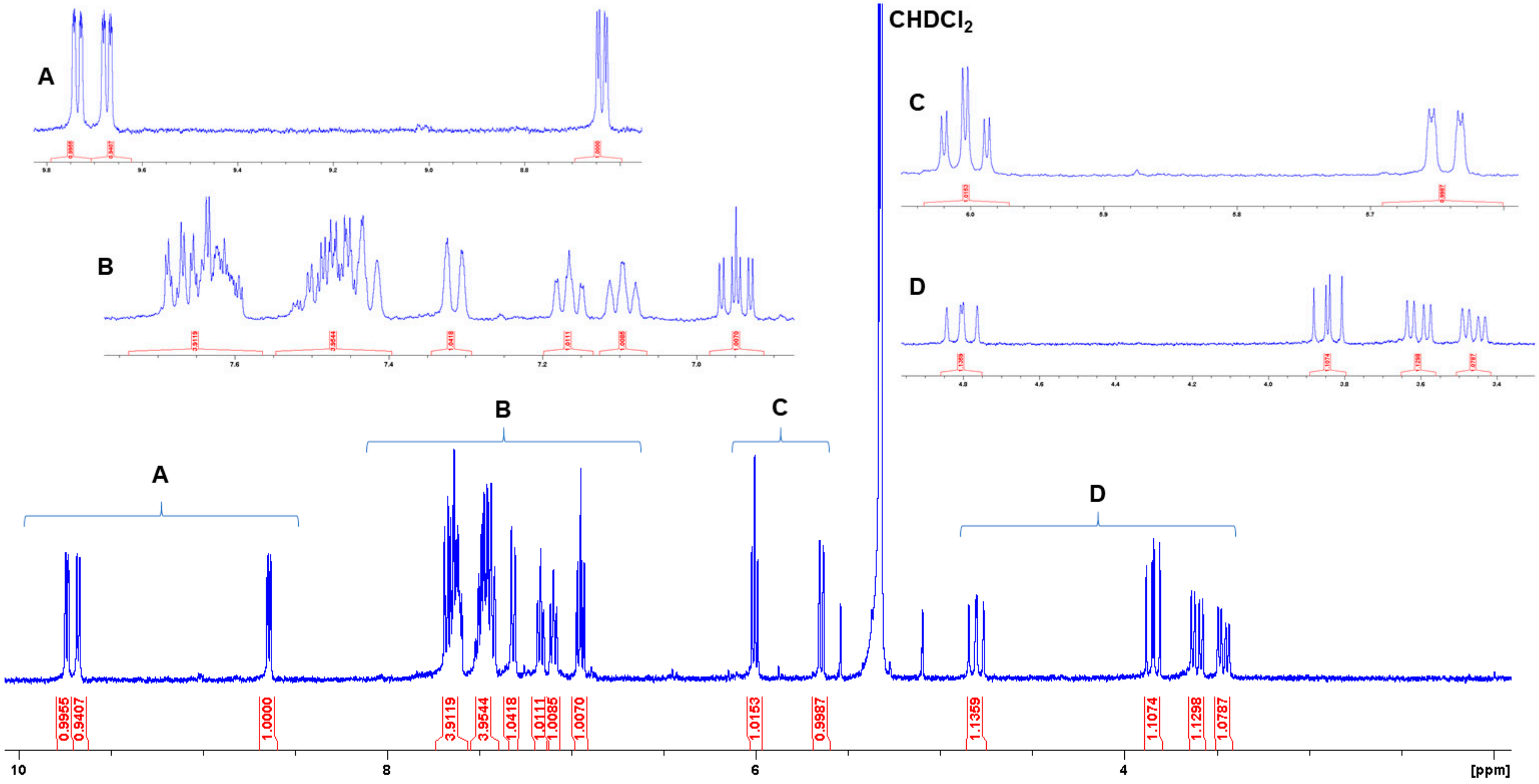

Figure S17. ^{1}H NMR spectrum of **6a** in CD_2Cl_2 (full spectrum and magnified insets of the (groups of) signals).

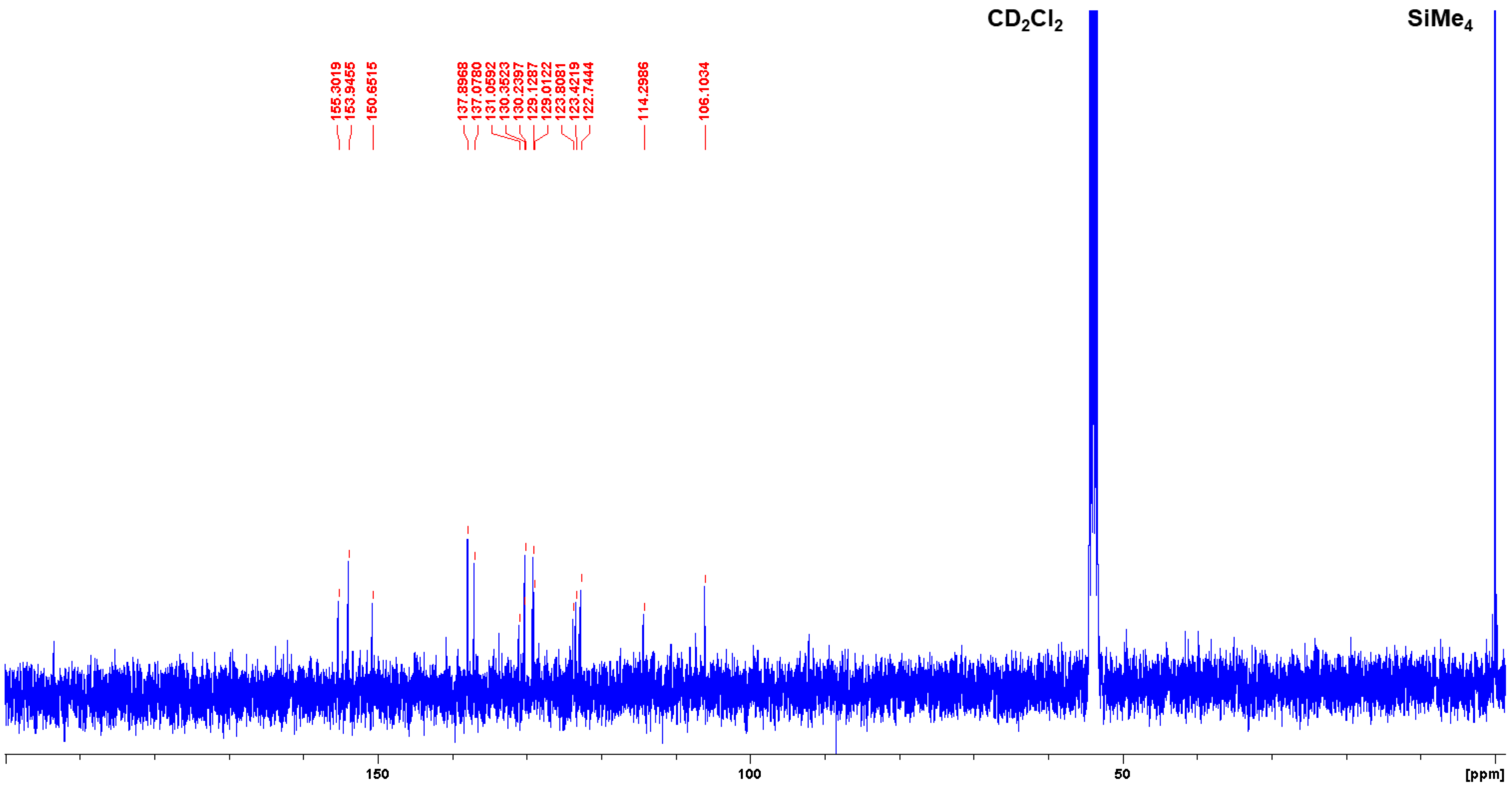

Figure S18. $^{13}C\{^1H\}$ NMR spectrum of **6a** in CD_2Cl_2.

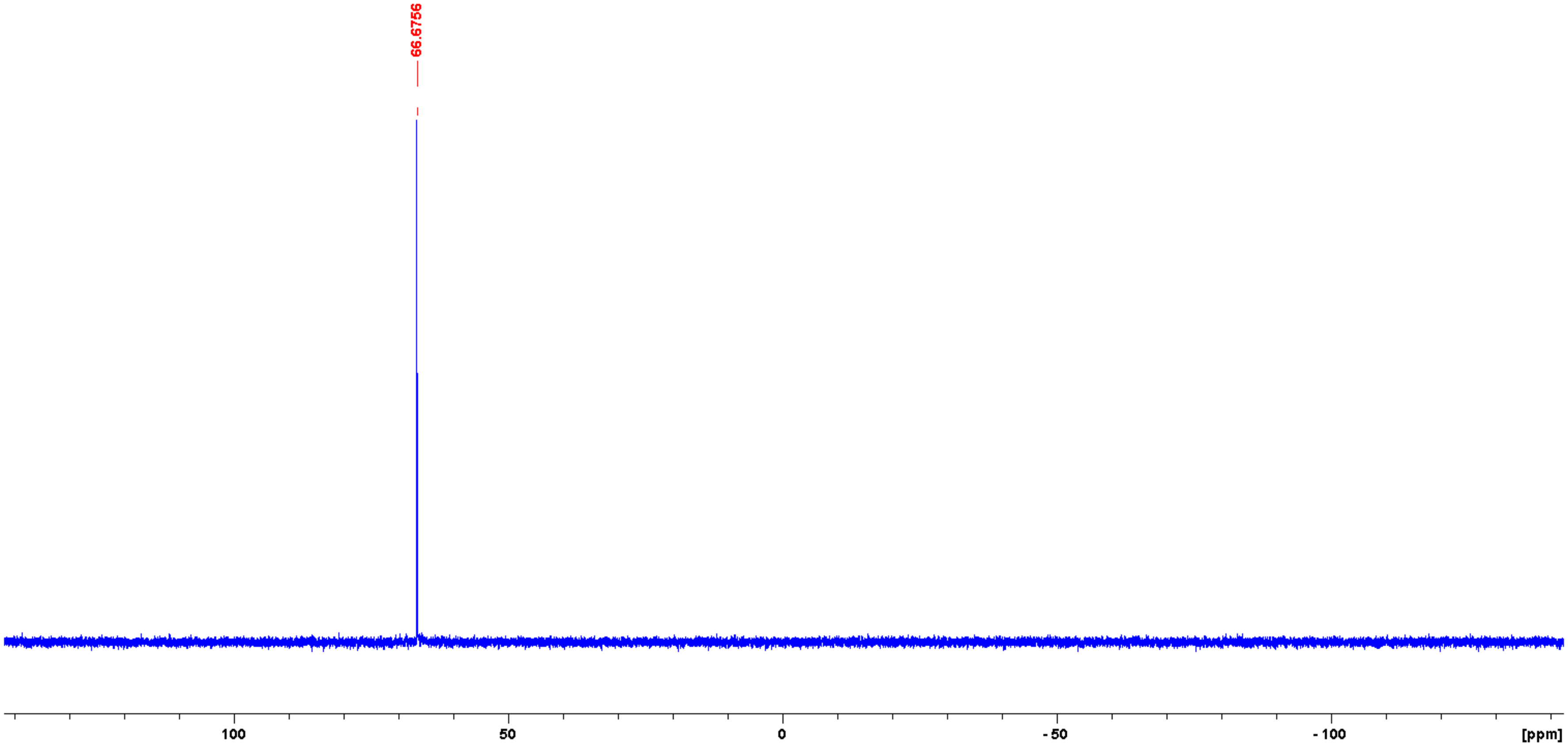

Figure S19. $^{31}P\{^1H\}$ NMR spectrum of **6a** in CD_2Cl_2.

Atomic coordinates and total energies:

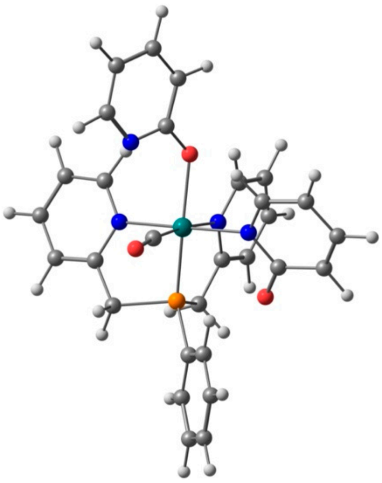

Figure S20. Optimized molecular structure of **4a[1]**.

PBE0:

final single point energy: -6486.191719155920 a.u.

final Gibbs free energy: -6485.76177128 a.u.

B2T-PLYP:

final single point energy: -6486.310311707651 a.u.

Table S1. Atomic coordinates for optimized structure of **4a[1]**.

Ru	-0.02773736464471	-0.00673784686152	-0.02021576328089
P	-0.49610970402804	-0.32214235073202	-2.15919168984598
N	2.06367414715394	-0.73437943278406	2.39908723802237
N	-0.11968931530034	2.07138009787470	-0.06060824920702
N	-0.16210750211666	-2.11613148417693	0.02584984487839
N	-2.20247879444173	0.02063239742565	0.12008587980439
O	2.89217633311975	-0.00097402851669	-0.62392060059277
O	-0.03796201814640	0.10986881531962	2.09404778767272
O	-1.45491412031034	2.03097612845957	-1.91807438928506
C	1.62744354795586	1.98338669499742	-4.74128857961091
C	3.05372940815865	-1.03289238602413	3.23914885775050
C	1.15668891730184	1.58382044255530	-5.98445018987244
C	3.00582269999925	-0.83023794459394	4.60337535452885
C	1.10644840797852	1.42171710831081	-3.58676353066982
C	1.78348109764484	-0.01084012868604	-0.32369095033027
C	0.16560341852026	0.61677877117072	-6.07257142904395
C	1.83734052545134	-0.27152878841472	5.12367037031212
C	0.11607312084144	0.44933778635959	-3.67114744385181
C	0.12519579349579	-2.05376209939462	-2.38075605455660
C	0.51948483115892	2.79507922836550	0.87373578892120
C	0.94089367077246	-0.19181683539707	2.88852575331884
C	0.46923526700478	4.16276888502735	0.94547070630882

C	-0.34970370867559	0.04562399504321	-4.91871793101813
C	0.80400752153445	0.04928082273533	4.27801800091766
C	-0.12465494980071	-2.81873872309202	-1.12437057983693
C	-0.31700515605683	-4.19313274780068	-1.12570283985973
C	-0.29835744918506	4.83608746845049	-0.01310183505931
C	-0.53835584126491	-4.85864017139647	0.06616069817970
C	-0.38335483186439	-2.76040537643581	1.17603274516121
C	-0.57174142132647	-4.12582712210922	1.24061056862068
C	-0.87268174162834	2.69822721305471	-1.02925740244502
C	-0.95292324211542	4.12301075373278	-0.97668105721587
C	-2.31287585365197	-0.53492717234366	-2.26824928904799
C	-2.96567499913510	-0.18400573780368	-0.96590329257251
C	-2.78539436304496	0.34409687959992	1.27854965570924
C	-4.34824998273461	-0.08001584946983	-0.89299676147198
C	-4.15283029584798	0.46928383835677	1.41533252579314
C	-4.95152503714517	0.25055261470283	0.30532454702512
H	2.39724829080454	2.74197876086517	-4.66914466611448
H	3.94195525304406	-1.46314399862347	2.78264304648856
H	1.56062568111933	2.02879094500662	-6.88583127593049
H	3.84410781389564	-1.09369507063491	5.23429677261702
H	1.45670970047863	1.74771351592478	-2.61525130572000
H	1.20463206018501	-1.95431944431540	-2.53805520984480
H	1.08953781245510	2.22230531158613	1.59248083182308
H	1.74360924908378	-0.08656064038878	6.18833652609375
H	-0.20661400942175	0.30538757626828	-7.04121201223853
H	1.01078638456367	4.68527289443333	1.72123555519771
H	-0.28620226820484	-4.72783387841062	-2.06597009430039
H	-0.28422915795138	-2.57185618271984	-3.24954357744571
H	-0.68565933389811	-5.93155970049227	0.07667441335470
H	-1.12153839775297	-0.71234881614667	-4.99648345507112
H	-0.37054152612810	5.91829118312605	0.00466304089906
H	-0.11478759515592	0.49117531740478	4.64259217763969
H	-0.41543557717663	-2.13676877532960	2.05732832611958
H	-0.74521549425227	-4.59527080630337	2.19933316849315
H	-1.55120060478135	4.60575304136941	-1.73830227602291
H	-2.55194336864746	-1.56821563911952	-2.53536850114903
H	-2.70437819672801	0.11344595595549	-3.05153905857350
H	-2.10196216868096	0.50068222683203	2.10372027938822
H	-4.93718011962128	-0.24709283489909	-1.78546763046321
H	-4.57357780423112	0.73817642720507	2.37475114950877
H	-6.02892908662398	0.34266695989721	0.36918648600093

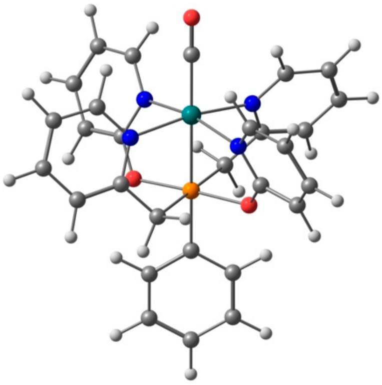

Figure S21. Optimized molecular structure of **4a²**.

PBE0:

final single point energy: -6486.139749933685 a.u.

final Gibbs free energy: -6485.70646114 a.u.

B2T-PLYP:

final single point energy: -6486.254919115781 a.u.

Table S2. Atomic coordinates for optimized structure of **4a²**.

Ru	-0.00029551628752	-0.00056381182322	-0.01516193433960
P	-0.00303539454365	0.00112071114623	-2.38940230901220
C	0.00146357476217	-0.00165477935627	1.88380255716843
O	0.00184658312277	-0.00202915778004	3.03851350699207
O	-1.73661600689301	-0.70015463127037	-2.43067358933372
N	-2.07468865784575	-0.01428334068210	-0.25666915360816
N	-0.20716647012891	-2.08310663047519	-0.19073126708820
N	0.20652541080879	2.08220991217971	-0.18833135727331
N	2.07349532347212	0.01326249165590	-0.26141224207047
C	-2.54539527130097	-0.46236818224408	-1.44663016481728
C	-3.92459674783431	-0.66412789660858	-1.63501354975354
H	-4.25301576912298	-1.03414674406366	-2.59643026718095
C	-4.80418515830377	-0.37963794793247	-0.62196843323256
H	-5.86737983001626	-0.53115912239608	-0.76573569156552
C	-4.31363814604352	0.11477273067917	0.58656615810550
H	-4.96752872442400	0.36422952771552	1.41041419102514
C	-2.95707795810178	0.27474812379470	0.71682194733690
H	-2.52682478698471	0.64529149778624	1.63700676379903
C	0.10023005433164	-2.67516140265148	-1.36685067645787
C	-0.22977960931446	-4.01539125085582	-1.58433169836253
H	0.02477242141114	-4.46041420529271	-2.53720624564984
C	-0.85779521030678	-4.75169902024261	-0.60460502047122
H	-1.10982696716758	-5.79185797983041	-0.77278744060191
C	-1.15604487224636	-4.13295719129751	0.60299830463940
H	-1.65005052616411	-4.65834273306804	1.40888949812577
C	-0.81417206609385	-2.80942358683538	0.76032743556730
H	-1.04151666120771	-2.28901492567866	1.68000631007428
C	-0.74331949554577	1.85798831340421	-2.39141997159903
H	-0.71558512747331	2.36178524460002	-3.35598304424378
H	-1.79076296306941	1.69310032877842	-2.13808881065318

C	-0.10364595566320	2.67597174295854	-1.36287358768553
C	0.22650326829690	4.01633050612471	-1.57943288942788
H	-0.03030680155892	4.46274727120786	-2.53104870334900
C	0.85745379887288	4.75100214144869	-0.60037518145847
H	1.10959771595654	5.79125006293345	-0.76783973418370
C	1.15851285418811	4.13051854508463	0.60564737895779
H	1.65488565684945	4.65458412013371	1.41094269839539
C	0.81635039748493	2.80696198264006	0.76211538017716
H	1.04581162421169	2.28521773648107	1.68051379666825
C	2.54150685658787	0.46296296792462	-1.45181742434897
C	3.92031439208968	0.66463563719463	-1.64315071797769
H	4.24658146763991	1.03596832767079	-2.60479336508746
C	4.80220155521247	0.37843952405207	-0.63258386056771
H	5.86509709227994	0.52988122659629	-0.77862971722848
C	4.31436776341166	-0.11758354247791	0.57639010843960
H	4.97013301035245	-0.36839659952224	1.39833432065721
C	2.95807597159398	-0.27739237186888	0.70960235043738
H	2.52983639201548	-0.64912347648645	1.63024905790839
C	-0.00521727712879	0.00268006684817	-4.27084430396039
C	-0.72669790439288	-0.94628052010932	-4.99151934792266
H	-1.29795385381951	-1.69702518164280	-4.46259409113418
C	-0.73484744574259	-0.94418252301126	-6.37933706010299
H	-1.31219734619456	-1.69141916420681	-6.91212856201341
C	-0.00851113315920	0.00511358510178	-7.08236026834777
H	-0.00977917904656	0.00605225021736	-8.16603143821630
C	0.71947077841804	0.95319694170801	-6.37939495145247
H	1.29556951589067	1.70135731471227	-6.91224449089080
C	0.71457205184725	0.95289167931910	-4.99156611780429
H	1.28702609426355	1.70274434404272	-4.46267255041739
C	0.73697001525277	-1.85556392443785	-2.39602457732196
H	1.78523633344681	-1.69142016782449	-2.14557043334964
H	0.70633167782022	-2.35797091875073	-3.36123076032281
O	1.73045485123480	0.70239164658274	-2.43359967258817

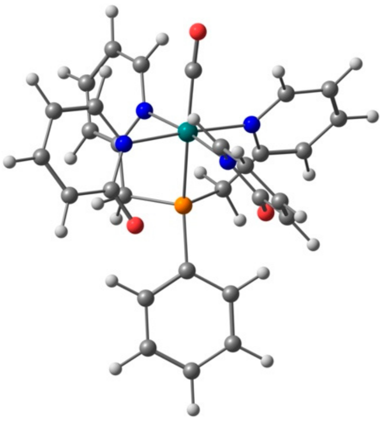

Figure S22. Optimized molecular structure of **4a^{3}.**

PBE0:

final single point energy: -6486.169901841126 a.u.

final Gibbs free energy: -6485.73860629 a.u.

B2T-PLYP:

final single point energy: -6486.285439713944 a.u.

Table S3. Atomic coordinates for optimized structure of **4a^{3}**.

Ru	-0.10435741886510	0.02314191180024	-0.05057292322127
P	-0.47426587043174	-0.02309523426012	-2.38004872379095
C	0.01421183171772	0.25396774907614	1.79125182107191
O	0.08404497615634	0.44049582017047	2.92440768878690
N	-2.22752234693958	0.05003750037013	-0.09069546310827
N	-0.21266795081481	-2.06342910694262	0.17288325652206
N	-0.00310132835470	2.10834812125085	-0.40497755886050
N	1.95917128054733	-0.08868979488429	-0.24351231818637
C	-2.88909427857296	-0.42094566096144	-1.16968350457040
C	-4.27842570179286	-0.42837329406576	-1.21002973401633
H	-4.77079043544110	-0.80983605407163	-2.09485154920144
C	-5.00946926876273	0.04148637035944	-0.13712249900910
H	-6.09206176297681	0.03337036460485	-0.16101393656350
C	-4.32378150943830	0.52828051084021	0.96372220367150
H	-4.83935959379593	0.91769027038015	1.83101441798035
C	-2.94609697192786	0.51575184347398	0.94173972996001
H	-2.38291162601683	0.89811288244918	1.78064496607312
C	0.25684036855744	-2.89110756231024	-0.83112429546888
C	0.26295800222643	-4.30193661740469	-0.57253549945334
H	0.67648655207066	-4.93032729680307	-1.35061770720790
C	-0.24315933020098	-4.82122811704763	0.58089863927799
H	-0.23615943086137	-5.89309518192993	0.74749468255587
C	-0.77658348011822	-3.95522973123799	1.54726732434629
H	-1.20258416167328	-4.31488840327211	2.47324977598159
C	-0.72752751429762	-2.61185677605190	1.29118234968816
H	-1.12184581287465	-1.91588045529770	2.02041208699125
C	-1.04894070293210	1.72637780834593	-2.58006785556517

H	-0.86352209768448	2.10469904521715	-3.58430850655923
H	-2.13422961684556	1.73115549586993	-2.44041450039091
C	-0.42618506811966	2.63060278698291	-1.57192445798124
C	-0.28741788437575	3.98461328237075	-1.84452673940959
H	-0.63914320236833	4.36313222933153	-2.79537161843247
C	0.30984819943348	4.82297237585620	-0.92415756989966
H	0.43074668431919	5.87829779975985	-1.13486370087226
C	0.76157737418012	4.27609744842557	0.26423067216511
H	1.25302907447839	4.87464094158200	1.01906821512950
C	0.58483915717772	2.92561166673298	0.47822328732516
H	0.94541824210527	2.46935340606209	1.38810690595980
C	2.56408154419412	0.51663486727788	-1.32531522834021
C	3.98697895143831	0.41277438647052	-1.42137919771433
H	4.45220756601551	0.90798968791386	-2.26363486576995
C	4.71439438699145	-0.29646334035795	-0.51087221664963
H	5.79173883183704	-0.37052219552503	-0.61301938257419
C	4.05788427529921	-0.93374047228798	0.55041338908434
H	4.58741105220121	-1.51798809367401	1.28958864791299
C	2.69819982241320	-0.79431856816822	0.63043999795803
H	2.14697704485926	-1.27417433844867	1.42912326845004
C	0.15098822904603	-0.45600175028125	-4.03123557578481
C	-0.14455825940962	-1.69499900366099	-4.59444700352297
H	-0.66704284375263	-2.44277294152181	-4.01617824628841
C	0.26510996495092	-1.99653483790930	-5.88385315527261
H	0.03109996651373	-2.96721871952857	-6.30453817798318
C	0.97814997784795	-1.06750987910251	-6.62638302457299
H	1.30150325611641	-1.30588893235634	-7.63273629342808
C	1.28273717951900	0.16524058036168	-6.06832731798547
H	1.85044776314082	0.89428434049695	-6.63424325078959
C	0.87347497579005	0.47043138675718	-4.77974563045421
H	1.14950009134889	1.41771730083846	-4.33996044806519
O	1.88663455122430	1.12678781846790	-2.18331338157443
O	0.63583846090496	-2.42092624048850	-1.92037019991800
C	-2.09564108769559	-0.90729190797108	-2.33350479225705
H	-1.84723285038267	-1.96224165043761	-2.19197796521016
H	-2.67272351689866	-0.81866120163596	-3.25507396096758

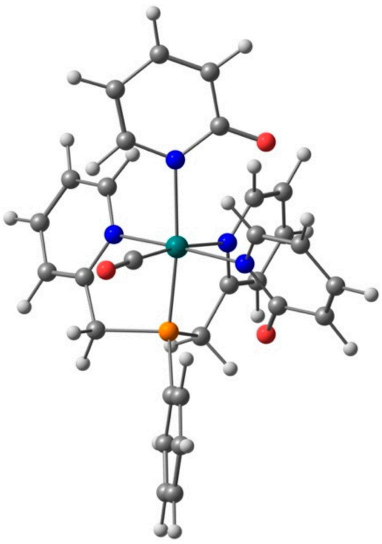

Figure S23. Optimized molecular structure of **4a[4]**.

PBE0:
final single point energy: -6486.183160077908 a.u.
final Gibbs free energy: -6485.75173398 a.u.
B2T-PLYP:
final single point energy: -6486.299848145378 a.u.

Table S4. Atomic coordinates for optimized structure of **4a[4]**.

Ru	-0.00259664499323	-0.05088962221398	-0.03864104523077
P	-0.00565755267723	-0.42931795723310	-2.23456221335483
N	0.08730097086876	2.01646276749678	-0.25398370694101
N	-0.32910674438226	-2.15466132224562	0.00896712436334
N	-2.18930163535003	-0.04331714551207	-0.42275990861071
O	2.96567924864886	-0.16273908971811	0.02433859576376
O	-0.92754637888334	1.85221378041390	-2.29653384502409
C	2.77936582535437	1.68063906562923	-4.30810648231591
C	2.57824632678882	1.26538764690103	-5.61716481672993
C	1.98196859338154	1.18351694735331	-3.28985561236279
C	1.81583240281681	-0.12707438559101	0.02845360236458
C	1.57936957962588	0.34709330786759	-5.90703812586251
C	0.98181360153172	0.26037780261312	-3.57638237905952
C	0.63055744870107	-2.17680719004519	-2.21474143906646
C	0.65848739025048	2.79399009521852	0.68000853192615
C	0.74317678224497	4.15765632798272	0.58738775741315
C	0.78637673257597	-0.15983451267520	-4.88860761432910
C	-0.02314114794257	-2.88918614780794	-1.08125847094079
C	-0.35483045167581	-4.23514013102254	-1.14919406518248
C	0.18716993942962	4.76895913225493	-0.54163923796550
C	-1.01263876875823	-4.83816678150241	-0.09280141091172
C	-0.98307237029205	-2.73725455509858	1.02022871274762
C	-1.33995232002862	-4.07006311505248	1.01163332250012
C	-0.43924383716662	2.57898132218531	-1.39185731410824
C	-0.39297953938641	3.99823863529593	-1.50924200185973

C	-1.73233510271846	-0.66531051837944	-2.76674525982520
C	-2.67170746381885	-0.38410787350729	-1.63203061377556
C	-3.05683224938860	0.23188821698685	0.55905906982077
C	-4.03732631613105	-0.47802445585457	-1.86096084100297
C	-4.42640347288269	0.15852650182188	0.39336618559330
C	-4.92972183099633	-0.21033179692543	-0.84063782493558
H	3.55375836371114	2.40241160871095	-4.07879162676530
H	3.19821169373150	1.66051861580612	-6.41283543611210
H	2.12405612523910	1.52752604238289	-2.27297071126909
H	1.70822052250463	-2.09492419760987	-2.03980428204948
H	1.06034826896942	2.27307539854957	1.53762069569309
H	1.41670711356683	0.02385376549585	-6.92821797327725
H	1.21479337545126	4.72506460640481	1.37692457389605
H	-0.09603541787512	-4.79552021189602	-2.03789451665027
H	0.48912097806370	-2.71563881518754	-3.15289463978940
H	-1.27524896460637	-5.88794359442531	-0.13666849989606
H	0.00956312049813	-0.87910282159166	-5.12367831932875
H	0.21994472720436	5.84733972189412	-0.65310200571498
H	-1.22737135389706	-2.09487781227915	1.85412056734956
H	-1.86933571954732	-4.48565755654479	1.85811344552039
H	-0.82563373005678	4.42874314184018	-2.40261262881770
H	-1.88794082914442	-1.68144089025177	-3.13768144758171
H	-1.94320970585581	0.02846465152176	-3.58057238065456
H	-2.61451738050761	0.58129927464627	1.48485190158462
H	-4.38582182920617	-0.75302807755389	-2.84820856223096
H	-5.07430568034684	0.40028064978010	1.22506085906076
H	-5.99724908379776	-0.27819090571691	-1.01203696314199
C	0.81042145023551	-0.86711441738312	2.77840057307348
C	-0.70483966349110	0.92325501755350	2.97537447088624
C	1.04205193378568	-0.95046049248931	4.12361264263981
H	1.32145340438244	-1.55119757861177	2.11073830369664
C	-0.48328376545735	0.86312620430725	4.39592214088304
C	0.36393280624729	-0.04356218404441	4.95475238443064
H	1.73329606913227	-1.68528036112510	4.51195922901631
H	-1.02683696094193	1.58319922380762	4.99422457137340
H	0.51504780722933	-0.06345749844655	6.02893702217914
O	-1.47901781787073	1.75631682972420	2.47124704872026
N	-0.02413850309670	0.01230086709583	2.18506571517886

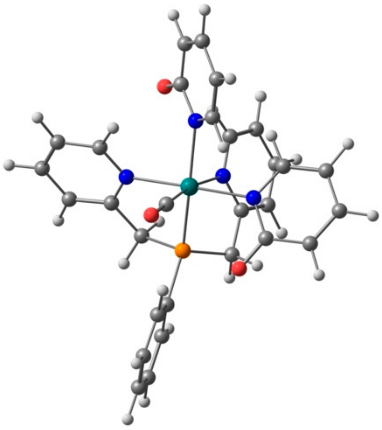

Figure S24. Optimized molecular structure of **4a^5.**

PBE0:

final single point energy: -6486.182564613791 a.u.

final Gibbs free energy: -6485.75104808 a.u.

B2T-PLYP:

final single point energy: -6486.298785766808 a.u.

Table S5. Atomic coordinates for optimized structure of **4a^5**.

Ru	-0.01037661585830	0.00835170705089	-0.06841897934506
P	-0.24411366782856	-0.06444284307486	-2.29802824001741
N	-0.26382245340255	2.08772814919330	0.05245196894303
N	0.10935950189953	-2.09504737022713	-0.28776084930539
N	-2.22769648993270	0.10801934369018	-0.35947910499807
O	2.95730275720406	0.15449567402791	-0.07053568412703
O	0.96678990496180	2.43047663665959	-1.84052997775934
C	3.04726585918919	0.49284043962440	-4.64538989978315
C	2.55574190779162	0.23859584388236	-5.91705936092108
C	2.20608421781701	0.43346830301802	-3.54473983177992
C	1.81021419950485	0.10565436813033	-0.06808180374041
C	1.21633724343494	-0.08187577845614	-6.09087158924603
C	0.86637757098182	0.10368429018433	-3.71248534069506
C	-0.89603755551288	-1.79293846414064	-2.47984072084701
C	-1.04881560675218	2.61276104184932	1.01101270770947
C	-1.29969541821537	3.95112952275198	1.15234573623262
C	0.37656406703991	-0.15657419689451	-4.99176972708563
C	-0.29059722562056	-2.67233430682794	-1.43580277181046
C	-0.14743722894398	-4.03745680033971	-1.64049217293487
C	-0.68510530215887	4.82674382167094	0.24608443738551
C	0.41765972372003	-4.82394631991817	-0.65464491370154
C	0.66613496291753	-2.85773183977872	0.65890040834230
C	0.83858880513044	-4.21827770465037	0.51690805543149
C	0.31510259095428	2.91368791318414	-0.89248022521824
C	0.10490814981867	4.32275444548524	-0.74510537913686
C	-1.73366160569558	0.93776134785769	-2.60904799331776
C	-2.69991597072723	0.65452561182767	-1.49889252721030

C	-3.09152537072988	-0.19655967965859	0.61579512008788
C	-4.05037611884117	0.92227804303712	-1.66270865750881
C	-4.44702680110649	0.05526147853694	0.51619183981274
C	-4.93648835524028	0.63430516857227	-0.64039545947755
H	4.09019271008304	0.75034570583883	-4.50696413127825
H	3.21495287900302	0.29369614606958	-6.77508018539045
H	2.57893866511112	0.67358835038214	-2.56079998972168
H	-0.73827437509201	-2.20770635684091	-3.47471643836117
H	-1.48492764720106	1.90071304305613	1.69876125883560
H	0.82551255399930	-0.27593380213427	-7.08241799980408
H	-1.94068082861397	4.30073200143231	1.94919218616926
H	-0.47849285125297	-4.46674259976376	-2.57700176733596
H	-1.97602365880530	-1.74138993549990	-2.31262828815831
H	0.53698889939890	-5.89005301610644	-0.80311048165611
H	-0.66614328966869	-0.41686369055468	-5.13771637840112
H	-0.83932367496072	5.89738285612959	0.32814248917079
H	0.99280140521720	-2.34293067638392	1.54866936204934
H	1.29837953288812	-4.78241450485379	1.31663986951966
H	0.58911804247145	4.96200706625135	-1.47160278616312
H	-2.19550883880796	0.74993819543936	-3.57966207453994
H	-1.40355394322428	1.97868804262996	-2.58849421942149
H	-2.65618703301673	-0.69545199412108	1.47596241130402
H	-4.39233102126706	1.35768004012824	-2.59261690788070
H	-5.09753165007360	-0.20972055468578	1.33874161837645
H	-5.99231647470060	0.84871282625894	-0.75323635704125
C	0.81106279063565	0.86161891747177	2.73329859904724
C	-0.65572131520682	-0.97483248914775	2.88590373019694
C	1.01157870029681	0.93361507680490	4.08499031260454
H	1.30379621993221	1.56946839619332	2.07863573088888
C	-0.47349970687556	-0.92185610667952	4.30748607084727
C	0.33848660373374	0.00452373608147	4.89144465689631
H	1.66872460731793	1.68668373948864	4.49646553267763
H	-1.01294660374856	-1.65811034295204	4.88960528155245
H	0.46198409553459	0.02151882578967	5.96915220439209
O	-1.38832671215216	-1.83248009810953	2.34607359790083
N	0.01507325724595	-0.03942012388069	2.12261137274633

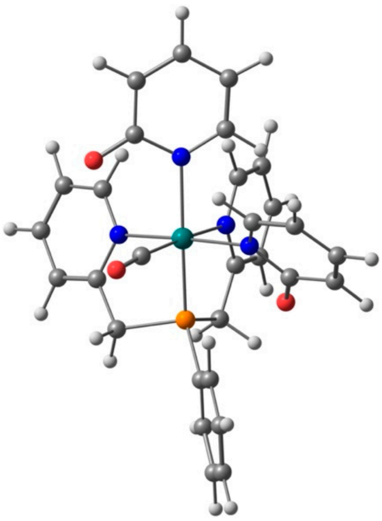

Figure S25. Optimized molecular structure of **4a[6].**

PBE0:

final single point energy: -6486.188266474338 a.u.

final Gibbs free energy: -6485.75679510 a.u.

B2T-PLYP:

final single point energy: -6486.303803226688 a.u.

Table S6. Atomic coordinates for optimized structure of **4a[6]**.

Ru	-0.00301665039932	-0.03869299179271	-0.06044288035532
P	-0.21417353658563	-0.17116721657111	-2.27413321574008
N	0.02092859274786	2.05764187735689	-0.00599159556884
N	-0.16640611489845	-2.14928270065182	-0.23991284990526
N	-2.21779121804771	-0.21964900633666	-0.20459342822515
O	2.96773071444956	-0.06495521971487	-0.24575412326717
O	-1.39710750995722	2.15624779839059	-1.79222619282833
C	2.16079045586790	2.47155766226514	-4.23681164894761
C	1.82981447772263	2.26668439212058	-5.56852724450584
C	1.52737976877740	1.74249277951151	-3.24252363746273
C	1.82699909547293	-0.06869370115005	-0.12609114410956
C	0.86438220985495	1.32873450754950	-5.90786526991903
C	0.56061391980534	0.80076993367997	-3.57866255155643
C	0.45615920533770	-1.86486085685421	-2.57163106534691
C	0.80397683219991	2.72002709383285	0.86594692429078
C	0.88000111925294	4.08496618950517	0.94592006689038
C	0.23304567859893	0.59452760723388	-4.91629399371789
C	0.03142554357763	-2.73835571748622	-1.43620242088849
C	-0.16748101573206	-4.10145745946978	-1.60387953595705
C	0.08800787504275	4.83359795737438	0.06529526573135
C	-0.57482565866672	-4.87351771409409	-0.53170619626560
C	-0.56871162789659	-2.89730771595953	0.79098554800335
C	-0.78548242158563	-4.25620586751282	0.68906062713710
C	-0.73164426431084	2.76148646831685	-0.92269537989434
C	-0.70008445698785	4.18858207782836	-0.84332544979316

C	-1.99018222443024	-0.33830551503269	-2.65907889403955
C	-2.80860323554049	-0.36516547341710	-1.40474474161145
C	-2.97888199792604	-0.32140443092366	0.89017559935856
C	-4.17873661196599	-0.57211391896204	-1.51073136795851
C	-4.34293552388816	-0.52345544820549	0.85661444831557
C	-4.96134588530657	-0.64190753377767	-0.37578888190811
H	2.90955414493497	3.20671634588723	-3.96848558531012
H	2.32249838450711	2.84068261021774	-6.34403412148203
H	1.77216174996769	1.91460822558312	-2.20186786848066
H	1.54566576790597	-1.75358454169432	-2.56479758268863
H	1.38793724466686	2.09956614017583	1.53272151813768
H	0.60239479797688	1.16895177599474	-6.94686309762782
H	1.53216801246696	4.54889541252683	1.67217938746805
H	0.00117804558053	-4.54234706037594	-2.57754878926250
H	0.17560559675029	-2.29906009721202	-3.53243554823769
H	-0.73053763935961	-5.93879205791051	-0.64982217719167
H	-0.51958981831860	-0.13692546764170	-5.18941455678994
H	0.10657446711139	5.91766532050729	0.09789224992927
H	-0.72565956200346	-2.37390056043180	1.72096172867900
H	-1.11087205993284	-4.81050156272215	1.55869681557477
H	-1.31541700462858	4.72745568195910	-1.55191207903492
H	-2.18487811822934	-1.24270000968876	-3.24093361014887
H	-2.28430532232526	0.52369644353403	-3.25737459190585
H	-2.46472507287816	-0.24345988920967	1.83621897256148
H	-4.61641585629797	-0.67773048615558	-2.49498643164489
H	-4.89647275529443	-0.58873462050786	1.78345208388077
H	-6.03047715757404	-0.79748338343954	-0.45200597942107
C	-0.72444442686025	0.90038348953342	2.78133517913635
C	0.89295902705185	-0.82297852203717	2.79285515025592
C	-0.72332009166989	1.04136750105827	4.14268738332327
H	-1.33503710772979	1.55624296067109	2.17136120001064
C	0.89454014523537	-0.71807961796254	4.22714734101301
C	0.11295009245335	0.18693132350885	4.88013903612911
H	-1.33911288703772	1.79472327667591	4.61339914130886
H	1.56584330950652	-1.38095866497114	4.75795759648165
H	0.14500221126537	0.25378844521929	5.96262417600044
O	1.63676494030922	-1.60632997789375	2.18027410865454
N	0.00584877286674	-0.00637244025144	2.10501274172722

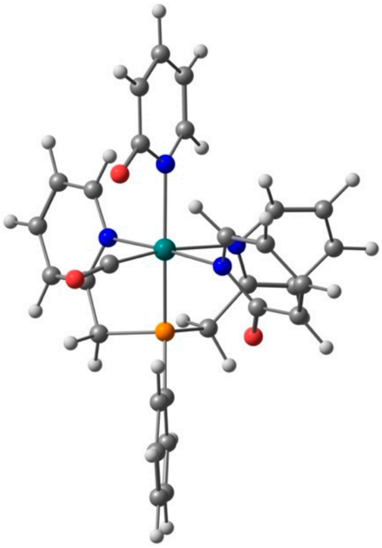

Figure S26. Optimized molecular structure of **4a**^{7}**.**

PBE0:

final single point energy: -6486.181286899863 a.u.

final Gibbs free energy: -6485.74935027 a.u.

B2T-PLYP:

final single point energy: -6486.295921648714 a.u.

Table S7. Atomic coordinates for optimized structure of **4a**^{7}.

Ru	-0.00167173230265	-0.07410620462441	-0.04948578450305
P	-0.31631756680167	-0.46846923783607	-2.22342185029743
N	-0.09558811459224	2.00165106055873	-0.26349167171677
N	-0.00789842488253	-2.24268257230820	0.02230845082365
N	-2.19147628980332	0.15868228806715	-0.14635353871124
O	2.94651821870965	-0.14871204370933	-0.49112916956034
O	-1.29228901133162	1.78590914015072	-2.21042593415093
C	1.93877967435031	1.70929273696068	-4.80218175834255
C	1.49767564527484	1.28910158394384	-6.04968586005892
C	1.36309527636868	1.19641232017721	-3.65121910397097
C	1.83350762608397	-0.10184921391408	-0.21960347863583
C	0.48302962306753	0.34796668698695	-6.14370311167873
C	0.34679940326028	0.25093935695374	-3.74209213994691
C	0.40636893143352	-2.16123588924800	-2.37339961664093
C	0.47710496834770	2.82289128500333	0.63605850452260
C	0.38378870968820	4.18983935033273	0.58671223536408
C	-0.08609361522501	-0.17673710031757	-4.99245726260242
C	0.13979934787215	-2.93364797328748	-1.12929520534991
C	0.07846988009766	-4.32076507850968	-1.15356823689837
C	-0.34370671632716	4.76096220906081	-0.46291434118599
C	-0.09582765549765	-5.02852273045096	0.01980333043439
C	-0.14210649210819	-2.93984557412551	1.15737099704829
C	-0.19224160517452	-4.31793108655381	1.20300551111786

C	-0.78934656320130	2.53166521638802	-1.33088722970989
C	-0.91427845988123	3.94986531771369	-1.40221917309614
C	-2.10522134165694	-0.85582311524372	-2.36572381252946
C	-2.85887552176193	-0.19058676889529	-1.26031915217500
C	-2.82455275527152	0.90998402531834	0.76070827761821
C	-4.20661339150568	0.10253768056964	-1.41436848066424
C	-4.15434798676310	1.26241002929778	0.65943255406842
C	-4.87002922495586	0.82487845577158	-0.44167434960199
H	2.72863267580674	2.44646002484241	-4.72401345992045
H	1.94388052303236	1.69688979135506	-6.94869291890106
H	1.69296824958686	1.54028829291548	-2.67837517038117
H	1.48686802259040	-2.01123905690461	-2.47475103357427
H	1.05908779820375	2.32950993661712	1.40273260773820
H	0.13418931486824	0.01874561168539	-7.11522087116651
H	0.87394793547971	4.78908842317102	1.34105073693202
H	0.18551562430870	-4.83060368894471	-2.10202756697277
H	0.07370499339730	-2.70683653239024	-3.25828069002822
H	-0.14135828035504	-6.11053083275084	0.01136424466209
H	-0.87341669097931	-0.91770601396923	-5.07726137175473
H	-0.44713157883006	5.83807760312052	-0.53893609998013
H	-0.20464648072055	-2.36367126229183	2.06610695848064
H	-0.30615338555282	-4.81192396271957	2.15841036613904
H	-1.47363707687393	4.34619644197887	-2.23940536864339
H	-2.21924128182785	-1.94213705987465	-2.30582536803703
H	-2.50853837786538	-0.53380470052675	-3.32555742231755
H	-2.22623590777088	1.25666777235808	1.59042682304836
H	-4.71258241568042	-0.20881723147533	-2.31872868453717
H	-4.60544373166406	1.87446229071428	1.42836387707628
H	-5.91851405279559	1.07044848111668	-0.55772505063401
C	-1.05996355904543	-0.45355761354386	2.84549911455123
C	1.14862412395759	0.35670937930496	2.81450997187580
C	-1.11779091715707	-0.53175852128347	4.21070205092159
H	-1.90820236013950	-0.79164177532798	2.26287211788593
C	1.11560529836564	0.30799977467611	4.25105382316808
C	0.01682151216838	-0.12202423012358	4.93005873936428
H	-2.00922122643609	-0.90207149582385	4.69737422148930
H	2.01646960324713	0.62208038685404	4.76206464240688
H	0.02139340666570	-0.15466492137401	6.01438922168489
O	2.16281460694629	0.71913269015660	2.19415514317489
N	-0.00320364644118	-0.01189412577295	2.13630849027948

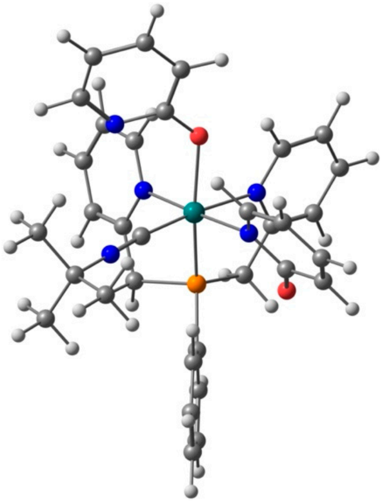

Figure S27. Optimized molecular structure of **5a[1]**.

PBE0:
final single point energy: -6623.485880982396 a.u.
final Gibbs free energy: -6622.94002378 a.u.
B2T-PLYP:
final single point energy: -6623.615457188704 a.u.

Table S8. Atomic coordinates for optimized structure of **5a[1]**.

Ru	-0.00681675557926	-0.11174778179668	0.01219446783453
P	-0.14476767232096	-0.43381925087722	-2.14743438052181
N	1.62371839903464	-1.03809783071178	2.82571647850250
N	-0.05260420449417	1.97345890108941	-0.04838411750868
N	-0.11142769212748	-2.21067351629110	0.05494079833114
N	-2.15944810548746	-0.10176180926904	-0.15354933216207
O	-0.26798933399350	0.04609051175766	2.12681577771847
O	-1.16083179908113	1.96968383580315	-2.05037510718320
C	2.36774596035771	1.90086500031484	-4.31979229866072
C	2.48417915924776	-1.28039499574003	3.81256622919675
C	2.11278652548697	1.52029960473470	-5.63044794011204
C	2.40091040776181	-0.73808960146614	5.07953564962315
C	1.65991788292741	1.32315329629628	-3.27804410317321
C	1.88448300941432	-0.06281325170640	-0.00288360154147
C	1.14933651608129	0.55788574609233	-5.89709044335542
C	1.33061321434232	0.12147754691093	5.33278621438464
C	0.69732713413245	0.35357377840741	-3.53992679485187
C	0.50483300361292	-2.16354707332378	-2.28820494921809
C	0.50648325073143	2.68428325785540	0.94353770487278
C	0.59128975664473	-0.20917969214777	3.05771376464915
C	0.51469087951230	4.05533040773495	0.99747282015357
C	0.44744990154851	-0.02864943270792	-4.85413253745404
C	0.42634275069994	0.38768849833892	4.33459845319074
C	0.09250774596655	-2.92407266618789	-1.07196323663705
C	-0.08148564667563	-4.30070702888158	-1.08795364379406
C	-0.10586478962512	4.74873242614943	-0.04864147700932
C	-0.45913796585847	-4.96086232636485	0.06759401169086

C	-0.48221060998274	-2.85103681878032	1.16894678222219
C	-0.66314937072091	-4.21854016900247	1.21867962686733
C	-0.65968659565535	2.62109924502839	-1.10407894867415
C	-0.67930974368045	4.04997205900507	-1.07313757763054
C	-1.92572676345724	-0.62532948729455	-2.54308260255325
C	-2.76158574660038	-0.31448316352224	-1.33636040890790
C	-2.90562268599796	0.18790544944380	0.91700232349179
C	-4.14391539678673	-0.25129846604745	-1.45119493551691
C	-4.28233882955929	0.27283233062201	0.86690390081407
C	-4.91550469723323	0.04634988583060	-0.34382190377472
H	3.11226027939874	2.65948989710056	-4.10915543630263
H	3.29970992769304	-1.95813085898536	3.56790214413069
H	2.66147623652973	1.97761715394809	-6.44509881089265
H	3.13720926163154	-0.97507751879827	5.83603600917441
H	1.83747462907341	1.63203527198339	-2.25542140999687
H	1.59477422622564	-2.05425276020540	-2.29570751133881
H	0.96105666518562	2.09871788381670	1.73130604652599
H	1.21329909385803	0.58119665139480	6.30839700114858
H	0.94403011178968	0.26260416921113	-6.91918509049225
H	0.98465223351755	4.56321550016775	1.82803969668361
H	0.08475228629507	-4.84170391433527	-2.01048729299525
H	0.22125779401309	-2.68771027437775	-3.20264963849303
H	-0.59543837874252	-6.03536316299643	0.06731748290909
H	-0.30164132370491	-0.78255705056343	-5.07084283110960
H	-0.12944057279494	5.83347282564048	-0.05017700541889
H	-0.41314974747871	1.05402876622154	4.48928296921475
H	-0.63654962654406	-2.22045869457582	2.03171565085734
H	-0.96293635188612	-4.68279271847037	2.14852510795259
H	-1.16388220512646	4.54808457111457	-1.90293964329871
H	-2.13223740410333	-1.64364764761415	-2.88440959175594
H	-2.19133345555797	0.06027246036202	-3.34736888873898
H	-2.34566403856517	0.35214143659402	1.82969785935724
H	-4.60081057711529	-0.42440267969510	-2.41703675192693
H	-4.83801939015638	0.51569514815629	1.76266602220632
H	-5.99391254564239	0.10614911456007	-0.42703156798979
N	3.04532950466922	0.02774397854272	-0.09691274536890
C	4.45684448631452	0.21586200153530	-0.15006954489064
C	5.07229257431591	-0.37863743647891	1.11257310047875
H	4.85013548240396	-1.44419636210423	1.18011386988391
H	6.15497388544877	-0.24490072503768	1.09028973203390
H	4.67296262046416	0.11242973339570	2.00018609945431
C	4.72832627489843	1.71600960381608	-0.22876324113030
H	5.80429587523286	1.89300113731951	-0.26619721559726
H	4.27193055291299	2.14050822744417	-1.12395194984856
H	4.31813608614739	2.22425684381797	0.64447093543220
C	4.98458451006795	-0.48999906266842	-1.39587945935064
H	4.77270060786988	-1.55907579508386	-1.34752999558712
H	4.51818638961655	-0.08076086156248	-2.29327114267392

H	6.06439450925888	-0.35176007188597	-1.46751919554915

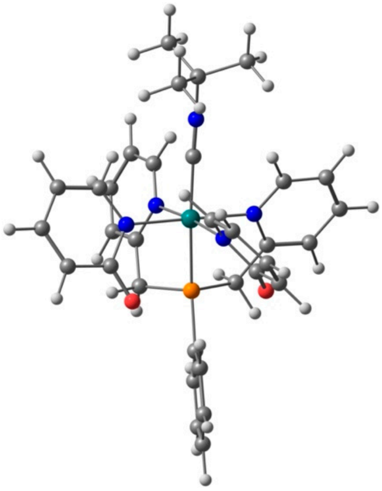

Figure S28. Optimized molecular structure of **5a³**.

PBE0:

final single point energy: -6623.466914284028 a.u.

final Gibbs free energy: -6622.91949136 a.u.

B2T-PLYP:

final single point energy: -6623.592996168088 a.u.

Table S9. Atomic coordinates for optimized structure of **5a³**.

Ru	0.04580701715467	0.02762901536721	0.02336912468331
P	0.26426005521373	0.16757381102720	-2.27643080677213
O	-2.21990057009921	-0.96711267180721	-2.01483308482947
O	1.59998358962458	-2.07996405411905	-1.70565483594250
N	2.15225095824487	0.09127676154845	-0.04639165036337
N	-0.07970491423420	2.12020370398298	-0.20522501345268
N	-2.06730323944070	0.00593095462161	0.05002656153511
N	0.13421119170165	-2.05127809185169	0.05766644974738
N	0.04937957831162	0.00503604082734	3.12426859036932
C	2.00483274459693	0.79490476292338	-2.38738406812317
H	2.50373146211297	0.40829776783435	-3.27551660587524
H	1.97968681073020	1.88488815225014	-2.48400483569825
C	2.79911463433124	0.42673778997305	-1.17939223541232
C	4.18661567929496	0.42628273325590	-1.24273478878516
H	4.66437425827291	0.70193045628242	-2.17391743187257
C	4.93642094272883	0.05563664058737	-0.14443806449244
H	6.01829864025432	0.04262924215146	-0.19170803072017
C	4.26571272250906	-0.31417650140863	1.00883830337718
H	4.79195301660869	-0.63381815681301	1.89828186121852
C	2.88755116413611	-0.28039320713222	1.01068167847795
H	2.33639913518314	-0.57740841307612	1.88990086837375
C	-0.68932387718235	1.72708630182508	-2.53778020278086
H	-0.49697557433138	2.23030121456832	-3.48642006411808
H	-1.73356012907797	1.40178999992914	-2.53681717983001
C	-0.43002304565099	2.65273184925464	-1.39789576784209

C	-0.52051484279471	4.02971454275953	-1.56670391219422
H	-0.79984893917523	4.41694411747590	-2.53789876133530
C	-0.26128418777944	4.88178613866318	-0.51125608587925
H	-0.33468558447934	5.95489756192593	-0.63698061599940
C	0.09954596992006	4.32843588440683	0.70679463847087
H	0.32158818446808	4.94331593714091	1.56865218783592
C	0.17838838678395	2.95643453955721	0.81132471135931
H	0.46385859636754	2.48847535234675	1.74261073081857
C	-2.79071355725996	-0.55143798922344	-0.98972373042287
C	-4.21798968789967	-0.61017022946576	-0.84699148324164
H	-4.76012110185942	-1.09177327721449	-1.65053689428472
C	-4.85592331720393	-0.06469666448566	0.22630629849272
H	-5.93717529610160	-0.11104613656270	0.30299312559761
C	-4.09675617596978	0.56432572090811	1.22383783801107
H	-4.54998924457675	1.03502619932229	2.08527444437094
C	-2.73428858924420	0.56216584527885	1.07909800662473
H	-2.12019310244078	1.03622747494747	1.83323070910234
C	0.94893206330527	-2.71111967488326	-0.84500773709766
C	0.99686313695466	-4.13928614774656	-0.75657821280223
H	1.65387507142767	-4.64417644700989	-1.45300350982980
C	0.23464371673617	-4.82833495521998	0.14239351438922
H	0.28138103271773	-5.91166388460216	0.17892731231151
C	-0.61391018681902	-4.12543220758995	1.00752127263348
H	-1.25375740215818	-4.62485080798960	1.72167813170612
C	-0.61806560809711	-2.75715759144103	0.91674282835281
H	-1.26476630648994	-2.17023838321940	1.55689121905270
C	0.05055205769695	-0.70145164776074	-3.85875370232684
C	0.39670105542337	-0.03548303630001	-5.03205980798950
H	0.76444151700481	0.98428894877058	-4.99540856857895
C	0.28369357282309	-0.66485164712493	-6.26332490160936
H	0.55584220994599	-0.13350505842473	-7.16769306047510
C	-0.17545965477187	-1.97131313838288	-6.32949048654853
H	-0.26460310472099	-2.46826043841008	-7.28838932298935
C	-0.52168964808781	-2.63909035921556	-5.16270458115906
H	-0.88292418186445	-3.65958327890537	-5.20863529600518
C	-0.41139613475359	-2.01037042220368	-3.93318493359033
H	-0.69786463740781	-2.52359716600206	-3.02910994497869
C	0.04020640739080	0.04816131575915	1.95463606670345
C	-0.07344833037014	-0.22632454439818	4.53054921740808
C	-0.42781802639687	1.09554954989093	5.20326966866120
H	0.35258551978208	1.83758793580307	5.02894232895508
H	-0.52869431498182	0.94295216077357	6.27852687620010
H	-1.37198119783187	1.48098393046363	4.81632540803831
C	1.26136317672966	-0.75612917407941	5.04381412891704
H	1.52004314794428	-1.68907292311865	4.54148913239704
H	1.19162675901616	-0.94503729978027	6.11583788405160
H	2.05670723145290	-0.03011618553157	4.86927886652353
C	-1.18073352277905	-1.25526107865961	4.73980947762026

H	-2.12545837373863	-0.88872080019368	4.33639280767174
H	-1.30431224260367	-1.44578178442484	5.80684991702995
H	-0.92966397922726	-2.19259790862592	4.24222274915871

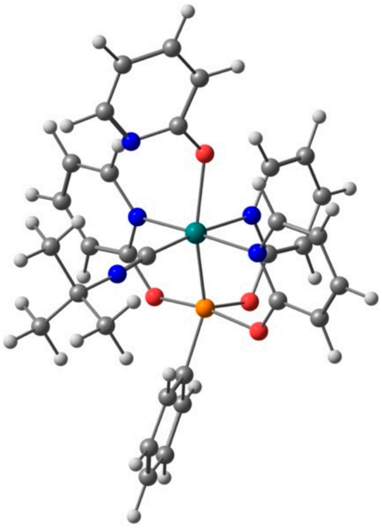

Figure S29. Optimized molecular structure of **5b[1].**

PBE0:

final single point energy: -6695.456175078394 a.u.

final Gibbs free energy: -6694.95599890 a.u.

B2T-PLYP:

final single point energy: -6695.619546962183 a.u.

Table S10. Atomic coordinates for optimized structure of **5b[1]**.

Ru	0.01763214349333	0.10498473790391	0.15734181108592
P	-0.24730357133079	0.26919094364059	-1.98943344342971
N	1.73144129179311	-0.68979298575274	3.00471093393193
N	-0.07077644328125	2.17488813715564	0.24681420936973
N	0.00601142963991	-1.95245636836645	-0.06828244096239
N	-2.14229878655077	0.06231897143534	-0.02771908887092
O	-0.31289343093788	0.02533340170426	2.26303806418001
O	-0.26519596542030	2.13548617062620	-2.03652127849560
C	2.56020746507797	1.02343585722712	-4.80662142002353
C	2.54881752751788	-0.89030059963703	4.03720742711900
C	2.00449081311256	0.51415730159669	-5.97162213226480
C	2.23826050099673	-0.60720342537345	5.35182507298100
C	1.85049170458866	0.96631727399498	-3.61738496479079
C	1.91994487097226	0.15011205807000	0.11420583502682
C	0.74287386061780	-0.06362280296712	-5.94122128740549
C	0.97200853769029	-0.07666588731405	5.60484169749166
C	0.59277992698418	0.37409620561579	-3.58439930016162
C	-0.00177151140468	2.88549064508133	1.38269406145968
C	0.51100849141142	-0.17867991384343	3.23654390158107
C	-0.05166165004333	4.25838764346512	1.40831835934310
C	0.04176985228034	-0.14846210560603	-4.74810408495267
C	0.10763011039232	0.13778568829505	4.56033541521769

C	-0.19014853462386	-2.41270361900737	-1.32012080646552
C	-0.23792883121871	-3.79162193301530	-1.57403192887217
C	-0.18179915906111	4.93569792537541	0.19697319330888
C	-0.08251663349814	-4.67554219267612	-0.53362913799574
C	0.16230298442263	-2.82643990650396	0.93765801650120
C	0.12448820016191	-4.18815611088964	0.75537790689258
C	-0.20074724429829	2.81900096373231	-0.92981130016423
C	-0.25726960874430	4.22029461980713	-0.97358619347517
C	-2.68921980586730	0.17165854830591	-1.24548184397982
C	-2.95316473391603	-0.08046241760525	1.03102421240336
C	-4.06613715445667	0.14575366160978	-1.44749243277663
C	-4.32344954788533	-0.11648568899757	0.91546647343099
C	-4.88678744276067	0.00002434225941	-0.35145130149514
H	3.54432261193701	1.47640827811156	-4.82505605098641
H	3.52252894588932	-1.30812304848717	3.79016342689392
H	2.55358334183061	0.56894775910220	-6.90399068341455
H	2.95085430005075	-0.79248302118240	6.14443403103288
H	2.27246381003480	1.38525877093157	-2.71184145952175
H	0.08665811801554	2.29506056203642	2.28399310077476
H	0.67028387076116	0.16714843305166	6.61791933269777
H	0.30453073702092	-0.45834900534560	-6.84997754401476
H	0.00928280118426	4.78294949236664	2.35157228434056
H	-0.39664367399615	-4.11974894627683	-2.59190697176225
H	-0.11879309226073	-5.74247290752561	-0.71979858857142
H	-0.93148772525974	-0.62066339635288	-4.72055193818476
H	-0.22524417439030	6.01819738247186	0.17350085239851
H	-0.88247647157844	0.54806323722396	4.71641039298482
H	0.34027464770720	-2.38240502555987	1.90619989182040
H	0.25831464090576	-4.84844168784380	1.60075543868180
H	-0.36148690251182	4.69862450020786	-1.93754135578821
H	-2.43785992048486	-0.15881338115908	1.98044925382240
H	-4.44983809670537	0.23946500750776	-2.45347773026117
H	-4.93567236498366	-0.23309711534618	1.79889676298895
H	-5.96185929981500	-0.02395347478384	-0.48138382951321
N	3.07678509352431	0.20067223619130	-0.02003310533804
C	4.49871972128486	0.17020311191403	-0.12665544187304
C	5.07032124615697	-0.32603035041089	1.19735216756245
H	4.70089319636949	-1.32748847521699	1.41956414952585
H	6.15925583634418	-0.35608330346755	1.13588091036002
H	4.77969897858286	0.33717051864766	2.01227607972370
C	4.98266695340213	1.58539434386166	-0.42832209030601
H	6.07000335627154	1.58898103301574	-0.51611478829869
H	4.55581855039119	1.94510775612931	-1.36562850033927
H	4.69346777347771	2.26759018922972	0.37181003019602
C	4.86641983688522	-0.77739389949684	-1.26511300298409
H	4.49869745041434	-1.78295664414232	-1.05798779323114
H	4.43229724963093	-0.43539225109344	-2.20549224475686
H	5.95144790923113	-0.81583942252778	-1.37244866194897

O	-1.89571195144386	0.31447132510732	-2.30920878029685
O	-0.32955172972819	-1.57129665023556	-2.30399634915601

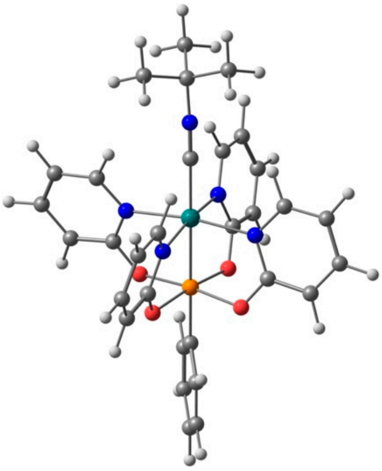

Figure S30. Optimized molecular structure of **5b³.**

PBE0:

final single point energy: -6695.449729791554 a.u.

final Gibbs free energy: -6694.95599890 a.u.

B2T-PLYP:

final single point energy: -6695.610018628110 a.u.

Table S11. Atomic coordinates for optimized structure of **5b³**.

Ru	7.66825868862418	3.80677450500988	3.96405647052781
P	9.24429743689518	3.79227125258897	5.62639095268228
O	8.56887920422945	2.23849704686397	6.40885567227506
O	10.44297906717978	2.75108843591173	4.64428324670889
N	6.67046086952581	2.39544663142573	5.11777616493715
N	8.79438375730235	2.27950972305552	3.10967557210924
N	5.54085981720760	3.81239945594992	1.65481578343171
C	7.36154069891206	1.84840841825620	6.14491009169035
C	6.78262612030041	0.84462667704991	6.94210017569681
H	7.37885568392824	0.43151139603646	7.74391859175993
C	5.50053395898038	0.42632297235577	6.69269283977373
H	5.05050265451335	-0.34574964305226	7.30546998403182
C	4.78592458843267	1.01267838808242	5.64796720348519
H	3.76985482746942	0.72378701328508	5.41755534463007
C	5.40967341850784	1.98028520689284	4.90083692714049
H	4.89945301537327	2.46179302324304	4.07843176462349
C	10.02206021775869	2.05776029801915	3.63424398366259
C	10.86380460056524	1.06955004271397	3.09214947911351
H	11.83724309690370	0.93475385331616	3.54291609576820
C	10.43533324462480	0.30882254496272	2.03489366808708
H	11.08013510988655	-0.45509571196575	1.61681620653179
C	9.15922660809528	0.52661760955502	1.51523186257271
H	8.77123291414753	-0.05341593250029	0.68939899901872
C	8.38963424245844	1.51115271670196	2.08248587665576

H	7.39474987599425	1.71773139078195	1.71397459742961
C	10.51538590874667	3.77806737027519	6.96885575437682
C	10.15270947451323	3.54639170589134	8.29169956647537
H	9.11523195944476	3.37415630622878	8.53785445858772
C	11.10558165564371	3.53472157971556	9.29960989070454
H	10.79826946031653	3.35280576181905	10.32304426350039
C	12.44205156067333	3.75351847933752	9.00196078624184
H	13.18758902323820	3.74372653924877	9.78858976708400
C	12.81379673383681	3.98495601959419	7.68630098117571
H	13.85444292806323	4.15726425337749	7.43661991532303
C	11.85857204622197	3.99765509250115	6.68068755303415
H	12.16245230294084	4.17866856203502	5.66014280157891
C	6.32583288431482	3.81392579615152	2.52566300493230
C	4.64127481675643	3.76193876452504	0.54576772425283
C	4.15150453960430	2.32473915598967	0.39606460290403
H	4.98959587931584	1.65104238962378	0.21302161506247
H	3.46115113490417	2.25898984931926	-0.44595612743218
H	3.63301753386708	1.99963006881112	1.29883193532759
C	5.40038075299818	4.20566644552411	-0.70138929084743
H	4.73582996243628	4.17262103947712	-1.56580595591003
H	5.76802778746657	5.22588119082772	-0.58369215490920
O	10.07586420534279	5.34277893694826	5.01202525201763
N	8.89339245265936	5.21185955787756	3.04290976485631
C	9.88937498947789	5.73901024946120	3.79202327887878
C	10.73870259914150	6.72650716837046	3.26159336914780
H	11.51312391085684	7.12277694506798	3.90367917664264
C	10.57311547547670	7.14939290861774	1.96756894158344
H	11.22696467563014	7.90827685186434	1.55442189258150
C	9.56024127392458	6.58355085256921	1.19339448075409
H	9.39485160721793	6.87578594193868	0.16566344265728
C	8.75891765866754	5.63128584022728	1.77197579728087
H	7.95926178299831	5.16621928642802	1.21303619149018
C	3.47522594706870	4.70297299667236	0.83060858454962
H	3.83069553111631	5.72636622048787	0.95728777478873
H	2.77310892821152	4.67874121536200	-0.00380815735789
H	2.95025137649570	4.40015743703878	1.73736562168451
H	6.24945314957567	3.54688567944007	-0.88801964840808
O	8.20808203957224	4.83187197727763	6.77931259887935
N	6.76607940427407	5.32750828368620	5.05600038840101
C	7.22770695845101	5.53733517634180	6.31043079591770
C	6.65147204157966	6.52592381324770	7.12867131610483
H	7.05348370684463	6.65240856155753	8.12430935757274
C	5.62281771244344	7.29659805739285	6.65124551588035
H	5.17848248098739	8.06080966770127	7.27785274263653
C	5.16595476650794	7.08805642960911	5.34975475563607
H	4.36360556552785	7.67455098081165	4.92413069036767
C	5.76466963968144	6.10378225701192	4.60410795349433
H	5.44481605714834	5.90309302217655	3.59125547615457

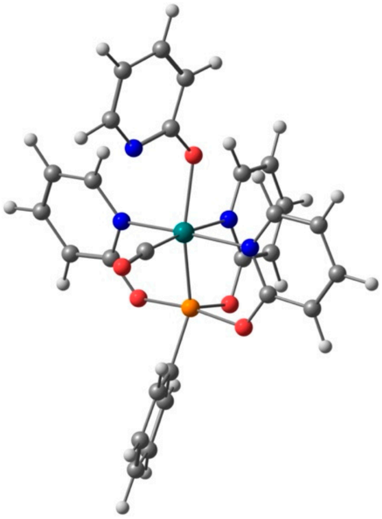

Figure S31. Optimized molecular structure of **4b¹.**

PBE0:

final single point energy: -6558.160689132269 a.u.

final Gibbs free energy: -6557.77707407 a.u.

B2T-PLYP:

final single point energy: -6558.322011793353 a.u.

Table S12. Atomic coordinates for optimized structure of **4b¹**.

Ru	-0.04677717339654	0.01632015042729	-0.06794781537517
P	-0.66653860079855	0.10770768756537	-2.17215514321226
O	-0.94835738872366	-1.74749709414429	-2.30806068196702
O	-2.33525787794148	0.26489471477728	-2.19940881489789
O	2.86785472385068	0.05925323210554	-0.73051675091074
O	-0.07697857038125	0.01751933884992	2.04631604443504
O	-0.53978572618595	1.93256426864635	-2.32725951005496
N	2.13059308540555	-0.48349029895016	2.34148545109092
N	-0.13819694271766	2.09193937371987	-0.07497092647392
N	-0.14470314728763	-2.04824885313307	-0.18203465490099
N	-2.22336124368331	-0.00966272280122	0.08689555409554
C	1.58939949775607	0.44651740099108	-5.51840736493274
C	3.15426481270778	-0.65114506238159	3.17774043812553
C	0.78347387353811	-0.09756043042700	-6.50753034575922
C	3.06946929423450	-0.51198889556098	4.54806654207071
C	1.12961520857769	0.52425899547086	-4.21284335563472
C	1.76883056278418	0.03149803509476	-0.40892749926231
C	-0.47904661296975	-0.57737184367166	-6.18712147740678
C	1.82369267246940	-0.17342722848704	5.07866702443376
C	-0.12977604224949	0.03141144396845	-3.89107628686793
C	0.02508841301842	2.85537577954372	1.01630761582142
C	0.93264671620857	-0.15347303653610	2.84098605512300
C	-0.03261697566435	4.22691289024389	0.97639208784337
C	-0.93243639746599	-0.52830460205489	-4.87864481665423
C	0.75346089950761	0.00660707521373	4.23692273136895

C	-0.62051379106383	-2.55009450338803	-1.33905606621259
C	-0.76261040576638	-3.93671822510532	-1.50457962661928
C	-0.27191802626893	4.84176879507478	-0.25134189579211
C	-0.41114533398041	-4.77855674760624	-0.47822711656739
C	0.20334205492043	-2.88145314391627	0.81053490239207
C	0.08690194526118	-4.24585895878596	0.71006258939081
C	-0.37864270497886	2.67470729991820	-1.26379811129004
C	-0.44716557164855	4.06961961752817	-1.37442946306387
C	-2.95417506856332	0.11052181373164	-1.02738328315069
C	-2.85556685952057	-0.16206777043310	1.26002394292298
C	-4.34525996160091	0.09037049986926	-1.01091959131963
C	-4.22672554081842	-0.19242083291299	1.36129220447432
C	-4.98225566342201	-0.06154637216567	0.20026176486719
H	2.57628664747828	0.81996705136612	-5.76284542856911
H	4.10106922432565	-0.91440771100902	2.71261636043861
H	1.13900504441112	-0.14792491894180	-7.52966596854770
H	3.93726039461964	-0.66061693497148	5.17648117132838
H	1.75228592406974	0.96858166741649	-3.44600926842624
H	0.19905665434752	2.30886446578015	1.93280737461733
H	1.69776920627474	-0.05077886152277	6.14896831366640
H	-1.11181620554938	-0.99992104946437	-6.95824302611993
H	0.10832547979070	4.79927262355143	1.88240041643352
H	-1.14699823328657	-4.30278339275034	-2.44630975370739
H	-0.51777164172909	-5.85023378464392	-0.59756897784724
H	-1.90711093241960	-0.92319954602799	-4.62447499444807
H	-0.32317558574231	5.92152558434239	-0.32435901580168
H	-0.22772504352549	0.27071425314546	4.61118132037163
H	0.59522006756467	-2.40480994127435	1.69712000774678
H	0.38318168475222	-4.87454918238018	1.53776748531858
H	-0.64017651252571	4.49867450006655	-2.34762571427913
H	-2.19768943427325	-0.24956646721115	2.11547142332927
H	-4.88234135941860	0.19170544399957	-1.94314518697842
H	-4.69249120374912	-0.31568291241838	2.32901710473384
H	-6.06439413155753	-0.08140667933095	0.24197455561144

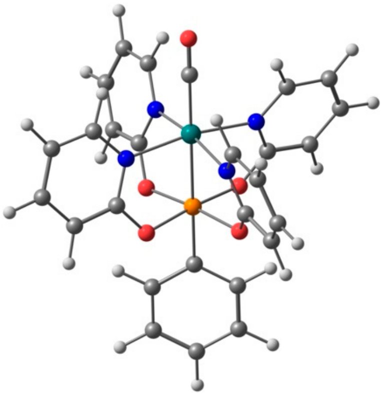

Figure S32. Optimized molecular structure of **4b³.**

PBE0:

final single point energy: -6558.156643837847 a.u.

final Gibbs free energy: -6557.77027133 a.u.

B2T-PLYP:

final single point energy: -6558.315616067810 a.u.

Table S13. Atomic coordinates for optimized structure of **4b³**.

Ru	-0.00148717715240	0.00118164661342	-0.05591876365613
P	-0.00119438663172	0.00061045824695	-2.38039112013570
C	-0.00135327940005	0.00104429952598	1.84226912045749
O	-0.00097598143180	0.00001923422801	2.99347970652635
N	-0.00233365233653	-2.07302986175485	-0.25399027426439
N	2.07252958957769	0.00092052549796	-0.25468090471864
N	-2.07543311557163	0.00164506302282	-0.25456339610934
N	-0.00085420770600	2.07525618940449	-0.25454309019378
C	0.28847754617067	-2.56941779251522	-1.47675879811576
C	0.35205890229835	-3.95685963579547	-1.69017937402882
H	0.59295422264386	-4.30325513594839	-2.68545343419931
C	0.10035606737105	-4.81920720797407	-0.65440961084762
H	0.14563291141885	-5.88985562586991	-0.81391759607052
C	-0.22297491788843	-4.30009778009199	0.59911375038343
H	-0.43843094951995	-4.93829299673823	1.44451278892023
C	-0.26134126773444	-2.93696383610765	0.74530339835345
H	-0.50599289867107	-2.49053876020516	1.69863676655958
C	2.56719292193962	0.30099145344790	-1.47578049189724
C	3.95384964731680	0.37581979402496	-1.68887154778294
H	4.29871054528220	0.62939434403318	-2.68159643144686
C	4.81789888470242	0.12228480390326	-0.65463725545790
H	5.88824535548908	0.17660375284180	-0.81333938906692
C	4.30080464192734	-0.21361950741693	0.59626406124160
H	4.94042116810156	-0.43087323625948	1.44012891225971
C	2.93779741178919	-0.26011279730305	0.74273988269936
H	2.49275940832804	-0.51253766169211	1.69471710427826
C	-2.57020351932102	-0.29910680253588	-1.47550218973631

C	-3.95696377156240	-0.37343990641760	-1.68840792962356
H	-4.30200084541870	-0.62759161018679	-2.68092611451727
C	-4.82084719920686	-0.11873573801976	-0.65434182558762
H	-5.89122584437722	-0.17267638627129	-0.81297159268199
C	-4.30357097299245	0.21786864124467	0.59631096234610
H	-4.94306198302062	0.43604554519680	1.44003186412110
C	-2.94054208435305	0.26382559662852	0.74268950034011
H	-2.49532757695617	0.51674002370697	1.69445821666284
C	-0.29160452376176	2.57123057223804	-1.47751734027316
C	-0.35551672802001	3.95863885304720	-1.69123324726645
H	-0.59634711204614	4.30474850323273	-2.68662356482370
C	-0.10424246056817	4.82129153103510	-0.65562413842748
H	-0.14979493835494	5.89189049096457	-0.81539230307993
C	0.21899289952282	4.30257700028371	0.59809802339852
H	0.43409961984441	4.94103570692884	1.44338664754076
C	0.25770642251849	2.93949189602879	0.74460596781468
H	0.50228376139300	2.49331590972560	1.69807861487300
C	-0.00172343335155	0.00038403884664	-4.22656277385646
C	1.04373896986461	-0.57886794256680	-4.93828906502558
H	1.86528887686919	-1.03486535819983	-4.40524213287380
C	1.04414086945108	-0.58045312870340	-6.32520389085658
H	1.86880658535203	-1.03871727139715	-6.85870417214297
C	-0.00233448635854	-0.00008846093034	-7.02569162803684
H	-0.00257681449400	-0.00027098984754	-8.10942146193077
C	-1.04850051428746	0.58051336738142	-6.32493033338088
H	-1.87340120437715	1.03859605124252	-6.85822294704214
C	-1.04749628441155	0.57939665967861	-4.93801960761790
H	-1.86880389285039	1.03555703825665	-4.40474738329641
O	-0.51826474859463	1.77338448877108	-2.47582309345744
O	1.76666507981033	0.52792466326484	-2.47267257682394
O	0.51553679079732	-1.77184630139565	-2.47525529765670
O	-1.76983510905116	-0.52709916034945	-2.47213893476877

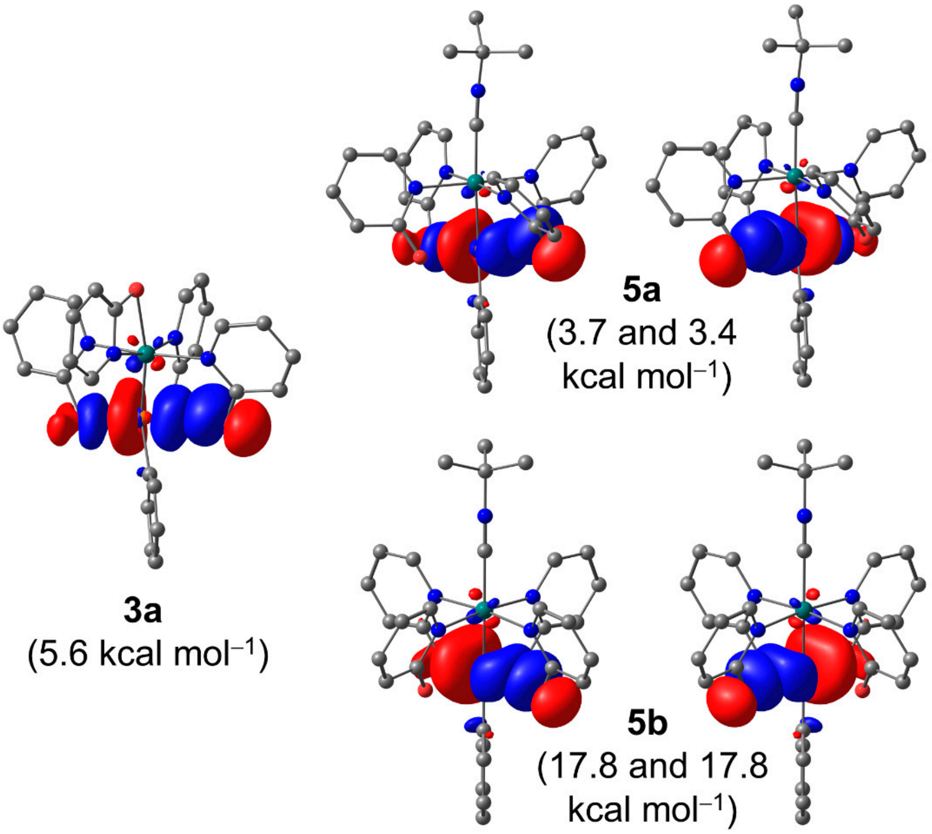

Figure S33. Graphical representation of main NBOs (isosurface 0.05 au) involved in σ-O→P donor-acceptor interactions in compound **3a**, compound **5a** and compound **5b** and energies of the donor-acceptor interaction as derived from second order perturbation theory analysis. Hydrogen atoms are omitted for clarity.

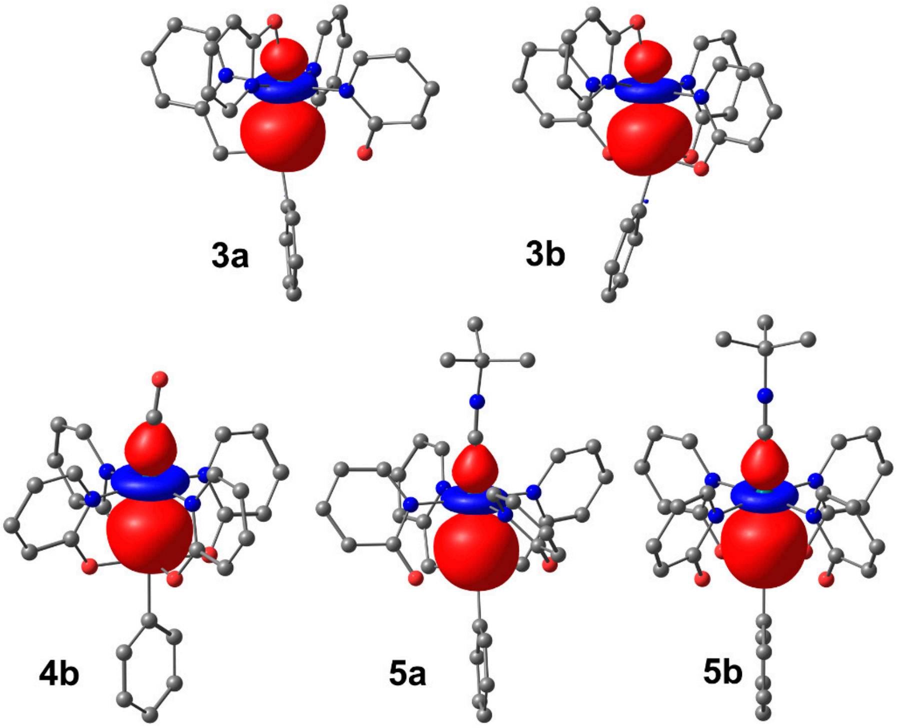

3a 3b 4b 5a 5b

Figure S34. Graphical representation of NLMOs (isosurface 0.05 au) representative of the σ-Ru−P bonds in compounds **3a**, **3b**, **4b**, **5a** and **5b**. Hydrogen atoms are omitted for clarity.

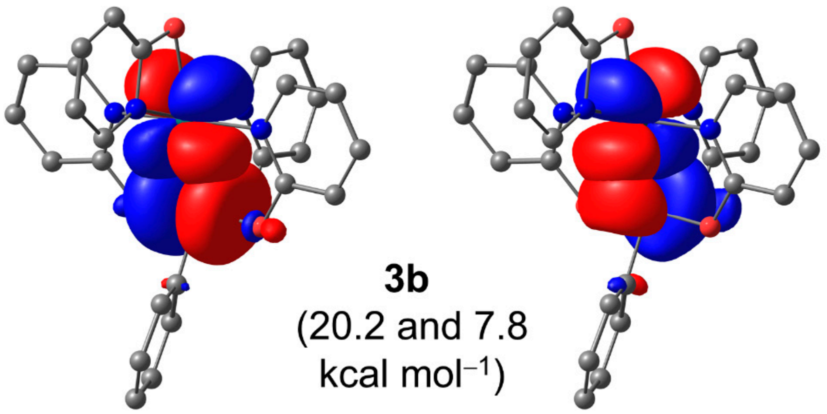

Figure S35. Graphical representation of NBOs (isosurface 0.05 au) involved in the predominant π-Ru→P donor-acceptor interactions in compound **3b** and energies of the donor-acceptor interactions as derived from second order perturbation theory analysis. Hydrogen atoms are omitted for clarity.

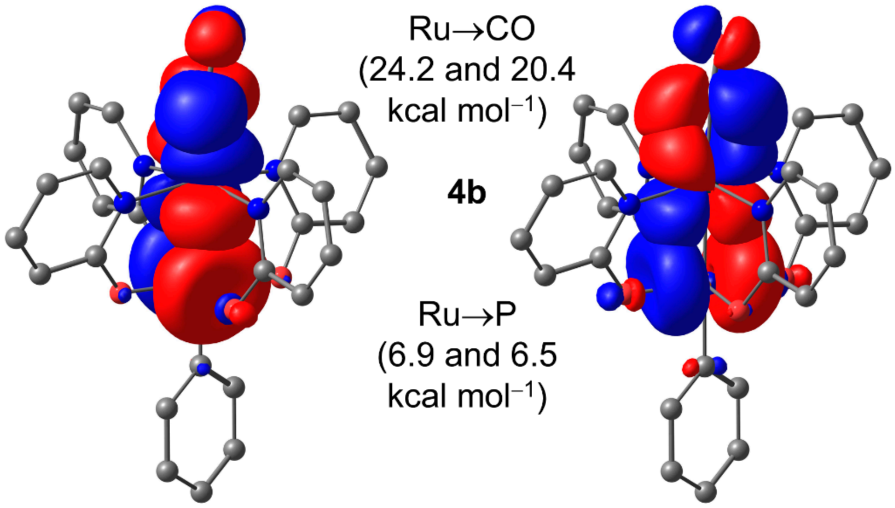

Figure S36. Graphical representation of NBOs (isosurface 0.05 au) involved in the predominant π-Ru→CO and π-Ru→P donor-acceptor interactions in compound **4b** and energies of the donor-acceptor interactions as derived from second order perturbation theory analysis. Hydrogen atoms are omitted for clarity.

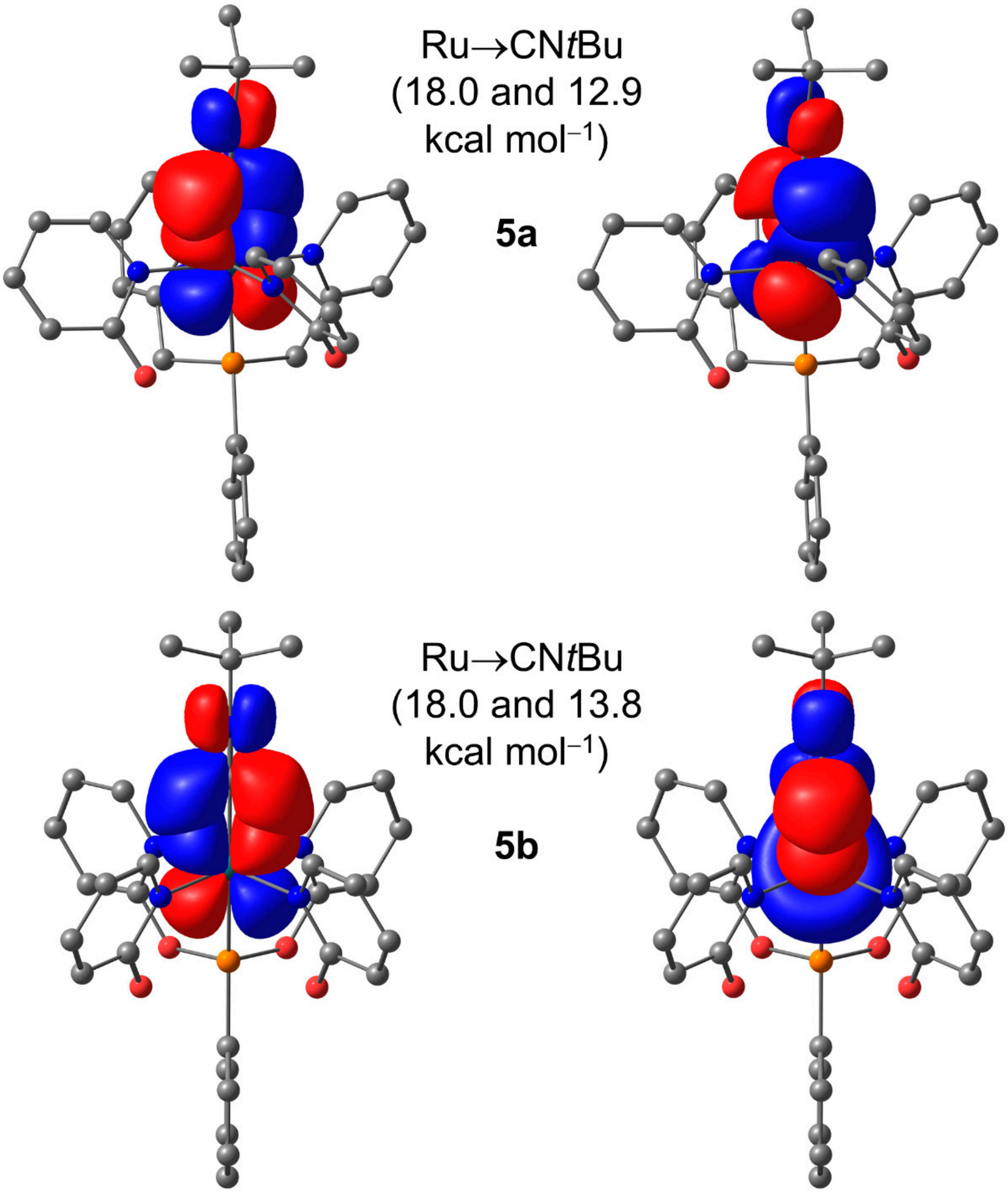

Figure S37. Graphical representation of NBOs (isosurface 0.05 au) involved in the predominant π-Ru→CN*t*Bu donor-acceptor interactions in compounds **5a** and **5b** and energies of the donor-acceptor interactions as derived from second order perturbation theory analysis. Hydrogen atoms are omitted for clarity.
